# Chloride Selective, Nonprotonophoric Ion Transport with Macrocyclic Halogen Bonding Anionophores

**DOI:** 10.1002/chem.202502033

**Published:** 2025-06-26

**Authors:** Martin Flerin, Fernanda Duarte, Matthew J. Langton

**Affiliations:** ^1^ Department of Chemistry University of Oxford Chemistry Research Laboratory, 12 Mansfield Road Oxford OX1 3TA UK

**Keywords:** anion transport, chloride, halogen bonding, membranes

## Abstract

Synthetic ion transporters hold promise as both chemical probes and potential therapeutics for diseases linked to malfunctioning protein ion transporters. However, their application in biological systems is limited, partly due to the cytotoxicity arising from unselective ion transport. Here, we demonstrate that highly active and selective anionophores can be accessed by combining halogen bonding anion recognition with macrocyclic anion encapsulation. Anion transport experiments in large unilamellar vesicles (LUVs) revealed over 300‐fold selectivity for chloride transport over proton/hydroxide ions, which is key for potential future therapeutic applications, where the dissipation of cellular pH gradients must be avoided. The mechanism underpinning selectivity is studied through Density Functional Theory (DFT) calculations and molecular dynamics (MD) simulations at the membrane interface, demonstrating that the cyclic structure imposes an energetic preference for chloride binding over hydroxide, as well as a greater desolvation of hydroxide, which further disfavors its transport. We anticipate that these results will accelerate the transition toward the use of artificial chloride transport in biology.

## Graphical abstract

Macrocyclic halogen bonding anionophores are reported, which enable very high chloride over proton and hydroxide transport selectivity. This ion selectivity profile is key for future potential therapeutic applications, and its origin is explored through DFT and MD simulations at the membrane.

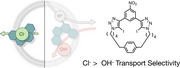

## 1 Introduction

Ion transport across lipid bilayer membranes is a tightly regulated process in living organisms, carried out by highly specific pumps and channels. Given the severe implications of genetic diseases that cause dysregulation of ion channels, known as channelopathies, much attention has been directed toward the development of synthetic analogues to natural transporters.^[^ ^1^, ^2^, ^3^, ^4^, ^5^, ^6^ ^]^ Among these are artificial ion channels, in which the self‐assembly of small molecular components is used to form pores in the membrane, and mobile carriers, which are small molecule ion receptors (ionophores) that shuttle ions across the membrane.^[^ ^1^, ^2^ ^]^

**Figure 1:**
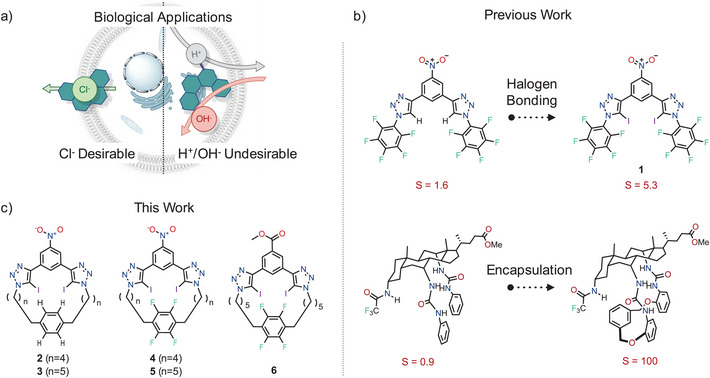
a) Anionophore selectivity for Cl^−^ over OH^−^ transport is crucial for many biological applications.^[^ ^1^, ^2^, ^5^, ^10^ ^]^ b) Previous work exploiting (top) halogen bonding^[^ ^16^ ^]^ and (bottom) encapsulation^[^ ^10^, ^23^ ^]^ to enhance Cl^−^ over H^+^/OH^−^ selectivity, *S*. c) The family of macrocyclic Cl^−^ transporters reported in this work that combine halogen bonding and anion encapsulation.

The structural design of mobile ion carriers is governed by their functional requirements: they must bind hydrophilic ions at the membrane‐aqueous interface while also transporting them across lipophilic membranes.^[^ ^7^ ^]^ This requires a delicate balance of directional noncovalent interactions for ion recognition, lipophilicity of the complex, and molecular size and geometry. Transporters that successfully integrate these characteristics could serve as biological probes for interrogating natural ion transport processes, as well as underpinning future therapies for channelopathies, such as those resulting from chloride channel misregulation, including cystic fibrosis.^[^ ^8^, ^9^ ^]^ However, to move beyond experiments in vesicle‐based models into cells, these transporters must also demonstrate a high degree of selectivity for the target ions. For example, chloride transporters typically also bind and transport other monovalent anions, including hydroxide.^[^ ^1^ ^]^ Moreover, their anion binding groups often comprise highly acidic N‐H hydrogen bond donors, which can become reversibly deprotonated and thus facilitate proton transport.^[^ ^10^ ^]^ These mechanisms of proton and hydroxide transport contribute to the dissipation of pH gradients, leading to cytotoxicity.^[^ ^3^ ^]^ Whilst this may be advantageous for inducing apoptosis—potentially aiding cancer therapy—this lack of selectivity limits the utility of chloride transport in other applications (Figure 1a).^[^ ^11^, ^12^ ^]^

One effective strategy to achieve chloride selectivity over proton/hydroxide ion transport has been to replace acidic hydrogen bond donors with Lewis acid halogen or chalcogen bonding groups (Figure 1b, top). Such halogen and chalcogen bonding interactions were first exploited by Matile and coworkers for mediating anion transport across membranes.^[^ ^13^, ^14^, ^15^ ^]^ Due to their higher lipophilicity and increased directionality compared to hydrogen bonds, halogen bonds have proven particularly advantageous for developing active ionophores and achieving some degree of chloride over hydroxide ion selectivity.^[^ ^16^, ^17^, ^18^, ^19^ ^]^ For example, we have reported iodotriazole and telluromethyltriazole halogen and chalcogen bonding anionophores,^[^ ^16^, ^17^, ^20^ ^]^ while Valkenier and coworkers reported an iodotriazole‐functionalized calix[6]arene derivative that exhibits Cl^−^>OH^−^/H^+^ selectivity.^[^ ^21^ ^]^ Another strategy to enhance such chloride selectivity is to increase the encapsulation of the anion binding site (Figure 1b, bottom), as exemplified by Davis’ macrocyclic cholaphane derivatives,^[^ ^10^, ^22^, ^23^ ^]^ and Gale's tripodal tris‐urea anionophores^[^ ^10^, ^24^, ^25^ ^]^ although the precise origins of this selectivity remain to be fully understood.

Size complementarity and ion solvation are crucial factors in the design of a selective transporter. For example, the oligodepsipeptide macrocyclic ionophore valinomycin exhibits remarkable selectivity for K^+^ over Na^+^, which arises from a high degree of size complementarity between K^+^ and the valinomycin binding site.^[^ ^26^ ^]^ Similarly, the size difference between chloride and hydroxide ions (ionic radii of 1.8 and 1.1 Å, respectively,^[^ ^27^, ^28^ ^]^) could, in principle, be exploited to enhance the selective binding of one over the other. On the other hand, as previously suggested by Wu et al.,^[^ ^10^ ^]^ differences in ion solvation also affect selectivity. Hydroxide has a significantly larger hydration free energy compared to chloride (‐105.0 vs. ‐74.6 kcal mol^−1^),^[^ ^27^, ^28^ ^]^ meaning that a more encapsulating binding site that promotes a greater degree of anion desolvation would disfavor OH^−^ binding in aqueous media in competition with Cl^−^. Increasing the degree of encapsulation of the bound anion, and hence the degree of anion desolvation, may therefore serve to maximize this discrimination, given that it has been shown that ion transport is typically also accompanied by the cotransport of water associated with the ion solvation shell.^[^ ^29^, ^30^, ^31^ ^]^

Here, we report the synthesis and characterization of a halogen‐bonding macrocyclic transporter family that exhibits over 300‐fold Cl^−^>OH^−^/H^+^ anion transport selectivity. Building upon our previously developed bidentate iodotriazole halogen bonding anionophore scaffold, which exhibited high activity but moderate anion selectivity (compound **1**),^[^ ^16^ ^]^ we developed a series of conformationally preorganized macrocycles, compounds **2–6**, employing convergent halogen bond donors for anion recognition (Figure 1c).^[^ ^16^ ^]^ The best‐performing macrocyclic transporter **2** exhibits remarkable selectivity for chloride over hydroxide in vesicle assays, representing almost two orders of magnitude improvement over the nonmacrocyclic analogue **1**. In vesicles treated to remove fatty acid contaminants, which can facilitate background proton transport, the intrinsic selectivity of the carrier is estimated to exceed 1000‐fold. Density functional theory (DFT) and molecular dynamics (MD) simulations reveal that the origin of this selectivity arises from a reduced energetic penalty and a lower desolvation cost upon binding of the chloride anion compared to hydroxide.

## 2 Results and Discussion

### 2.1 Synthesis of the macrocyclic transporters

To design a macrocycle that optimizes size complementarity and solvation discrimination, we replaced the two pentafluorophenyl substituents of **1** with a linker of varying lengths. To minimize the buildup of ring strain upon cyclisation,^[^ ^32^ ^]^ we surveyed available procedures in the literature for synthesizing a macrocyclic *meta*‐bis‐triazolo‐benzene motif, which revealed that the smallest reported macrocycle had a minimum of 15 atoms in the linker between the triazole N atoms.^[^ ^33^, ^34^, ^35^ ^]^ Accordingly, we took this to be the minimal length of the linear precursor used to form the macrocycle via a copper‐catalyzed azide alkyne cycloaddition (CuAAC) click reaction.^[^ ^36^ ^]^ We selected a 1,4‐bisalkyl‐benzene linker to provide steric shielding of the binding site while allowing for variation in length (compounds **2** and **3**). We also considered fluorination of the benzene ring (compounds **4**–**6**), as the unique solubility of perfluoro moieties has been shown to improve ion transport.^[^ ^29^ ^]^ Compound **6**, containing a less polar methyl ester electron‐withdrawing group, was also synthesized with the aim of improving lipophilic balance.^[^ ^37^ ^]^ A log*P* value of 5–6 has previously been shown to be optimum for anion transport,^[^ ^1^, ^37^, ^38^ ^]^ and the calculated values^[^ ^39^ ^]^ for macrocycles **2–6** ranged from 4.8 to 6.6 (Table S1).

**Scheme 1:**
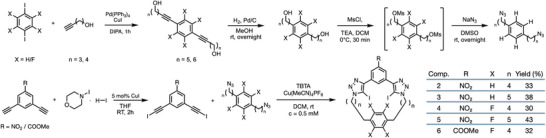
Key intermediates to macrocycles **2, 3, 4, 5,** and **6,** and cyclization conditions and yields.

Macrocycles **2–6** were synthesized using a convergent synthetic pathway via a symmetrical bis‐azide and bis‐iodoalkyne double CuAAC click macrocyclization step (Scheme 1).^[^ ^36^ ^]^ To synthesize the bis‐azide precursors, we furnished *para*‐bis‐iodobenzene derivatives with alkynes bearing terminal hydroxyl groups in a highly efficient Sonogashira reaction (for full synthetic details, see §2 of the Supporting Information). This was followed by reductions of the alkyne bond, installation of methylsulfonic groups on the terminal hydroxyls, and their displacement with azide moieties in the final step, yielding the bis‐azide. The symmetrical iodoalkynes were obtained from the corresponding alkynes and then coupled with the azides according to literature procedures.^[^ ^16^, ^36^ ^]^

In the final ring‐closing step, the formation of the [2+2] dimeric macrocycle side product was observed to compete with the formation of the desired monomeric [1+1] macrocycles. Analysis of the monomer:dimer ratio in the crude mixture for macrocycles **4** and **5**, as a representative pair differing only by chain lengths (n = 4 or 5, respectively), revealed a strong dependence on size and concentration (Figure S52). For **4**, the ratio of monomer:dimer shifted from ∼1:4 to ∼1:1 when the concentration of macrocycle precursors was decreased from 5 mM to 0.5 mM. For the larger macrocycle **5**, an increased ratio of 2:1 was obtained under the same dilute conditions. This indicated a significant strain‐induced kinetic barrier in the cyclization step, which is increased with a shorter linker, and suggested that the synthesis of macrocycles with even shorter linkers would likely not be feasible.

### 2.2 Computational Characterization of Macrocycle Preorganization

**Figure 2:**
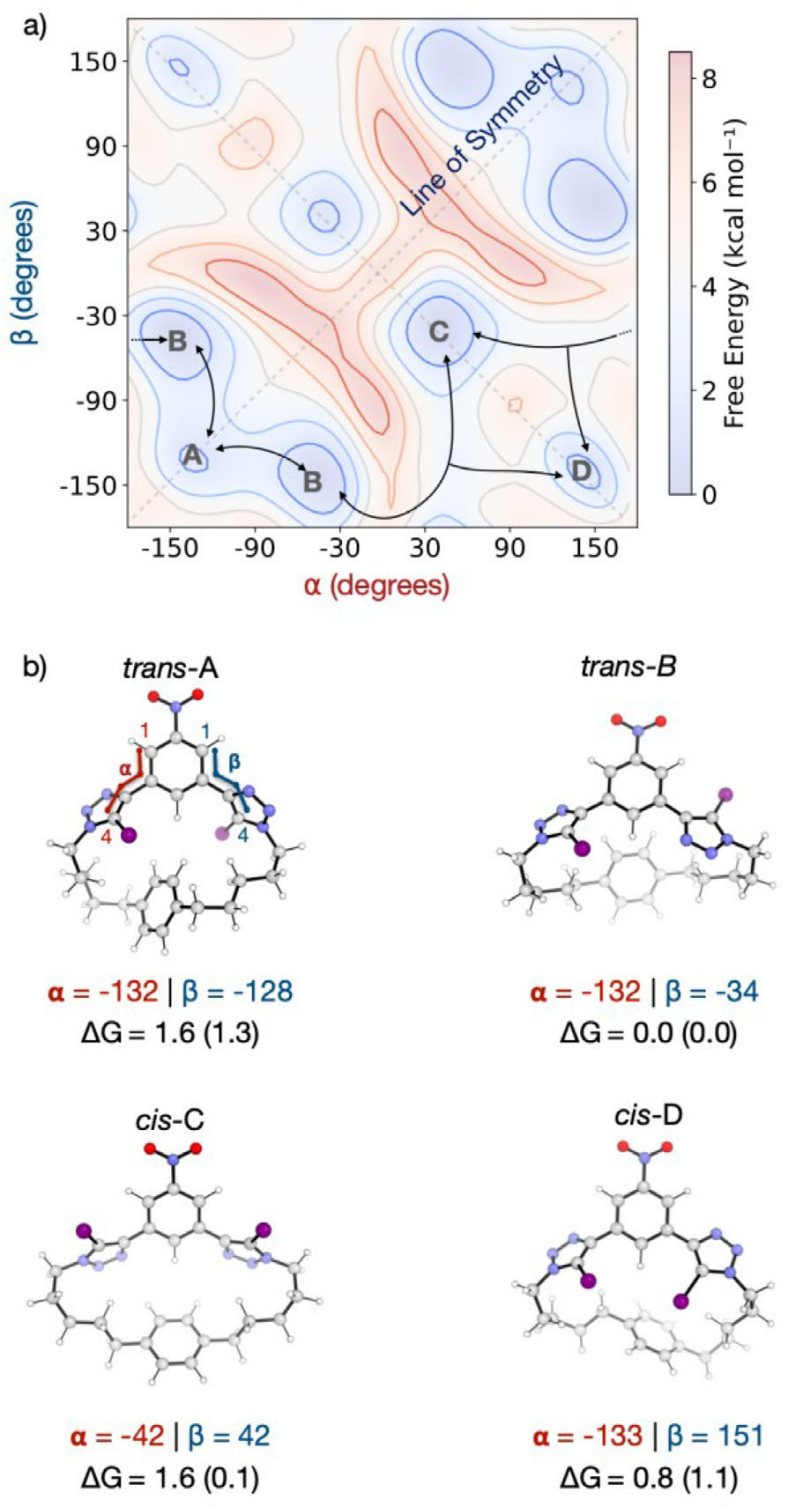
a) 2D energy profile as a function of torsion angles ⍺ and β for **2**, generated using 2D well‐tempered metadynamics MD (WTMetaD) simulations in explicit DMSO solvent at 298 K, using GAFF parameters for DMSO and the transporter, with iodine modeled using anisotropic charge following literature protocols^[^ ^42^, ^43^, ^44^, ^45^ ^]^ (§8 in the Supporting Information). b) Representative structures for each of the minimum identified in the 2D energy profile computed independently using CREST followed by DFT optimization at the SMD(DMSO)‐ωB97X‐D3/def2‐QZVP// SMD(DMSO)‐ωB97X‐D3/def2‐SVP level of theory^[^ ^46^, ^47^, ^48^, ^49^ ^]^ (highlighting angles ⍺ (red) and β (blue)). Energies are reported in kcal mol^−1^ relative to conformer B, with energies from WTMetaD presented in parenthesis.

Previous NMR studies on triazole‐containing macrocycles have shown that decreasing the ring size can increase preorganization by slowing down the interconversion of conformers.^[^ ^32^ ^]^ However, increasing ring strain may also impact the success of macrocyclization to form the desired macrocycle. To determine whether the smaller macrocycle **2** is sufficiently flexible to enable the two iodotriazole groups to adopt a conformation enabling convergent halogen bonding interactions for Cl^−^ anion binding, we computed a 2D free energy landscape as a function of the torsional angles of the iodotriazole‐nitrobenzene linkage using well‐tempered metadynamics (WTMetaD^[^ ^40^ ^]^) in explicit DMSO solvent at 300 K (Figure 2).^[^ ^40^ ^]^ The system was described with the General Amber Force Field (GAFF),^[^ ^41^ ^]^ using an extra particle (EP) point charge during the RESP charge fitting procedure to account for the anisotropy of the halogen atom. The iodine's Lennard‐Jones parameters were also modified to better reproduce QM binding distances (see §8 Supporting Information and Table S4 for details).

Four energy minima (A–D) were identified in the 2D free energy landscape, corresponding to *trans*‐ (A,B) or *cis*‐ (C,D) arrangements of the two iodotriazole moieties relative to the plane of the ring (Figure 2). Only the *cis*‐ arrangements allow bidentate Cl^−^ binding, with *cis*‐D being the most pre‐organized for binding due to its convergent halogen bonding groups. Representative conformers of each minimum were also identified via a dihedral‐constrained search using the conformer‐rotamer ensemble sampling tool (CREST), followed by DFT optimization and frequency calculations. Both MD simulations and DFT calculations indicate that all conformers are close in energy (within 2 kcal mol^−1^), with *trans*‐B being the global minimum. The conversion from *trans*‐B to *cis*‐C and *cis*‐D occurs via low energy barriers (ranging from 3 to 6 kcal mol^−1^), suggesting facile interconversion under experimental conditions. These results indicate that these macrocycles can readily adopt a convergent (*cis*‐) geometry necessary for anion binding despite the macrocycle's strain.

### 2.3 Anion Binding and Transport Experiments

Chloride anion binding affinities were determined by ^1^H NMR binding titrations with tetrabutylammonium (TBA) chloride in d_6_‐acetone containing 2.5% D_2_O, a competitive solvent mixture to represent anion binding to an anionophore at the hydrated lipid‐aqueous interface,^[^ ^16^ ^]^ by monitoring the binding‐induced changes in chemical shifts of protons in the benzene ring between the two triazoles. The data in each case could be fitted to a 1:1 stoichiometric binding isotherm using Bindfit.^[^ ^50^ ^]^

Transporters **2** and **4**, containing shorter pentyl spacers, exhibited Cl^−^ binding constants of 153 and 130 M^−1^, respectively (Table 1), while **3** and **5**, possessing the longer hexyl spacers, showed enhanced binding (*K* = 897 and 720 M^−1^, respectively). These results reveal that the shorter chains in the smaller macrocycle derivatives are detrimental to anion binding, presumably due to steric constraints. Furthermore, the fluoro‐ withdrawing methyl ester moiety in **6** significantly decreased the chloride binding affinity compared to **4**.

**Table 1:** Anion binding, transport, and selectivity data.

	*K* [M^−1^]^[^ ^a^ ^]^	EC_50 None_^[^ ^b^ ^]^ [nM]	EC_50 FCCP_^[^ ^c^ ^]^ [nM]	*S* ^[^ ^d^ ^]^
**2**	153 ± 6	>10^3^	34 ± 5	> 300
**3**	897 ± 78	>10^3^	68 ± 11	> 100
**4**	130 ± 25	>10^3^	50 ± 7	> 150
**5**	720 ± 34	*Inactive*		
**6**	36 ± 4			

^[a]^ 1:1 transporter‐chloride association constants as determined by titrating aliquots of Cl^−^ as the TBA salt in 2.5% D_2_O (v/v) in acetone‐*d*_6_ and fitting with Bindfit software.^[^ ^50^ ^]^

^[b]^ Effective concentration required to reach 50% of the maximum activity in the HPTS assay. Assay conditions: 200 nm POPC LUVs (31 µM lipid) containing 1 mM HPTS, 100 mM internal and external NaCl, buffered to pH 7 with 10 mM HEPES. A pulse of NaOH (5 mM) is added to the external solution to create a transmembrane pH gradient.

^[c]^ Same assay conditions, with the addition of a pulse of the protonophore FCCP to a final concentration of 0.25 µM.

^[d]^ Selectivity factor *S*, calculated as the ratio between EC_50 None_ / EC_50 FCCP_. Errors represent 95% confidence intervals.

**Figure 3:**
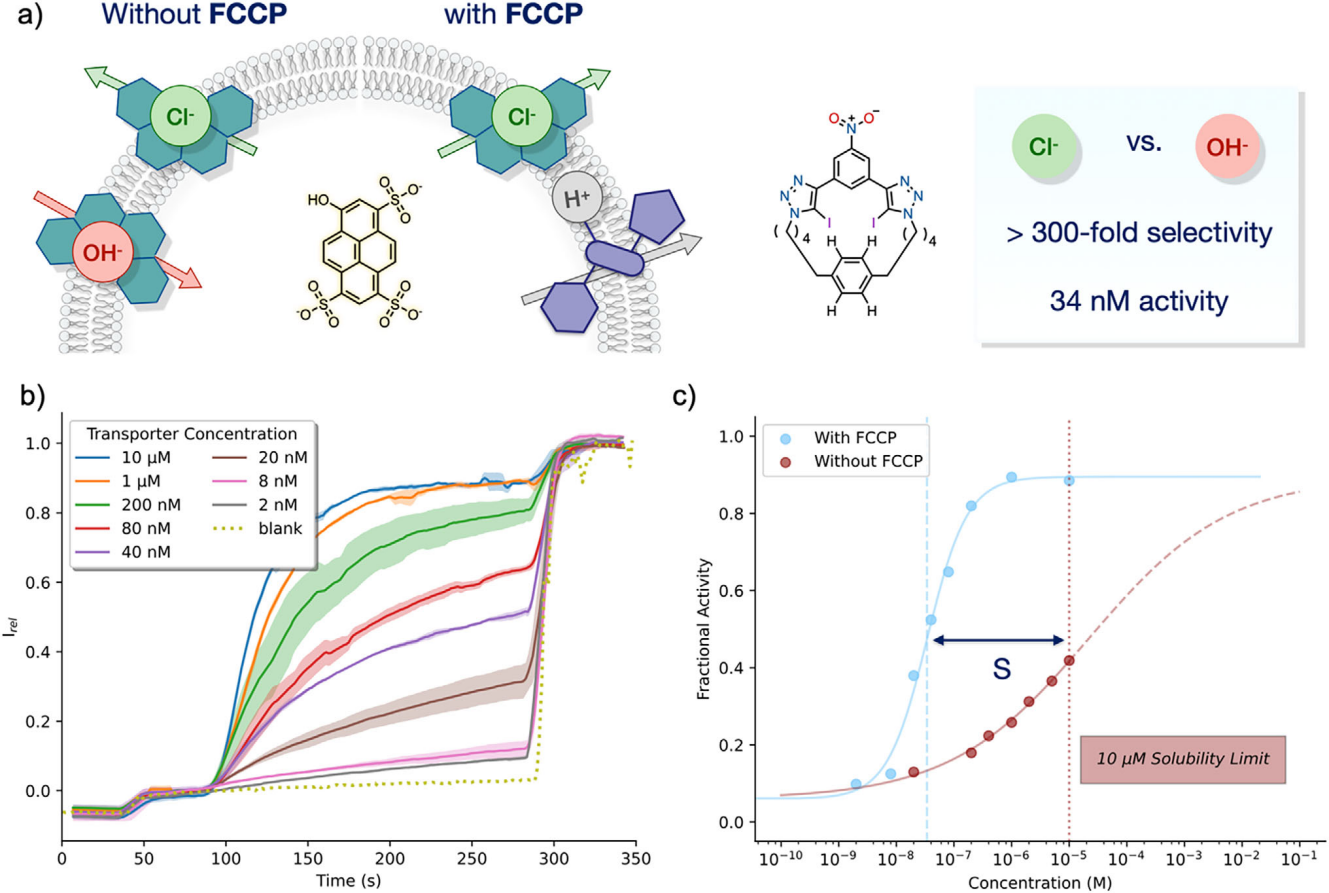
Transmembrane ion transport experiments. a) Illustration of the pH‐gradient transport assay procedure in the presence and absence of protonophore carbonyl cyanide‐p‐trifluoromethoxyphenylhydrazone (FCCP) to determine activity and selectivity, and summary of the results of best‐performing transporter **2**, showing the selectivity and activity in the presence of FCCP in POPC LUVs. b) Normalized transport data of **2** in the presence of FCCP. c) Hill plot for macrocycle **2** with and without FCCP.

**Figure 4:**
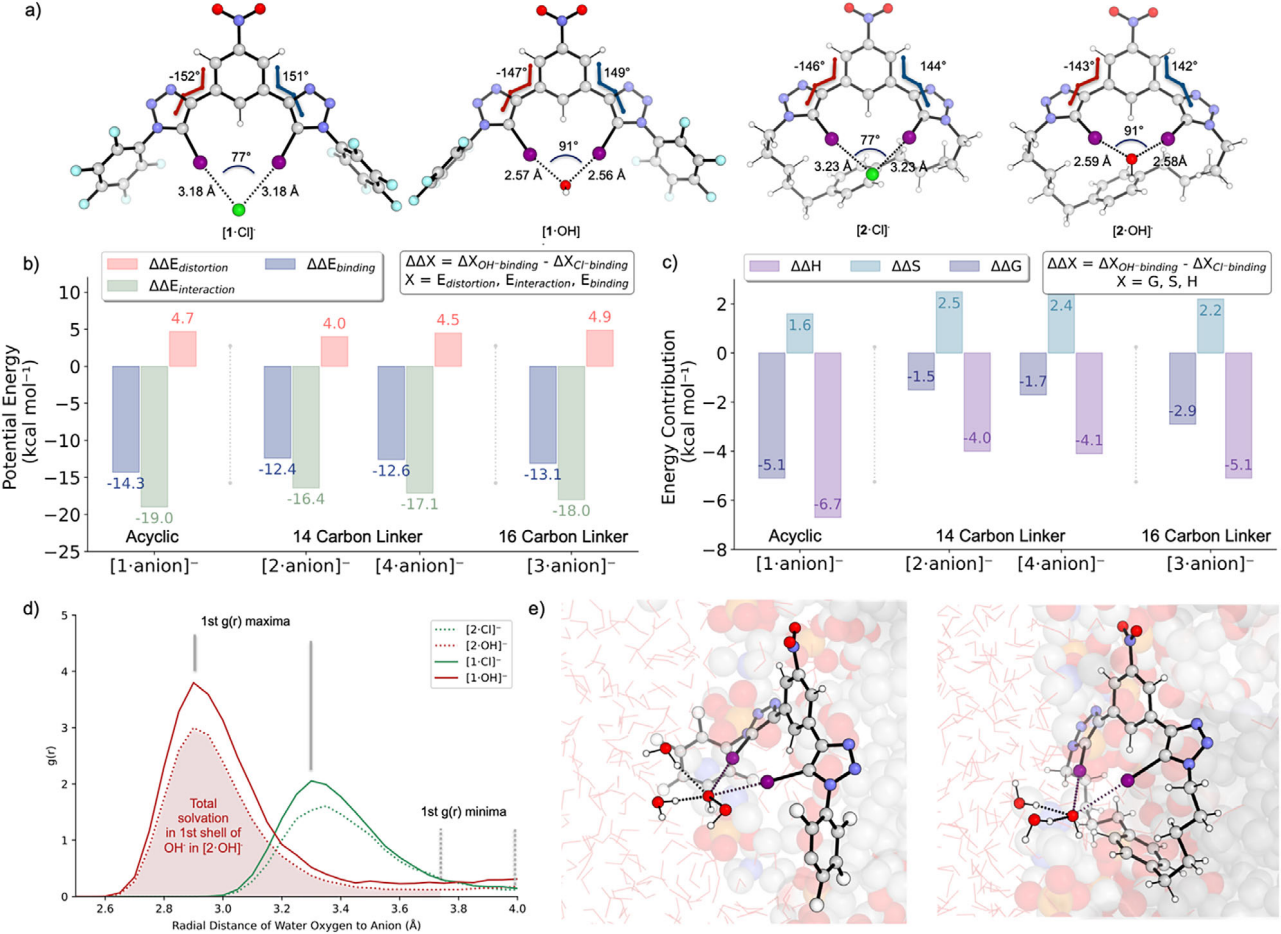
a) Geometries of **1** and **2** in complex with Cl^−^ and OH^−^, highlighting iodine‐ion distances (Å), I‐ion‐I angles (blue semicircle), and dihedral angles around the iodotriazole and nitrobenzene (red and blue lines). b) Distortion‐interaction analysis based on relative binding potential energy differences (ΔΔE_binding_), negative values indicating a preference for OH^−^. c) Partition of the relative binding free energy differences (ΔΔG_binding_) into enthalpic (ΔΔH_binding_) and entropic (ΔΔS_binding_) contributions, with negative values indicating a preference for OH^−^. d) Oxygen‐ion radial distribution functions (RDFs) for the [**1**/**2**:ion]^−^ complexes computed from 3×100 ns MD simulations at the membrane‐water interface. The first hydration shell is labelled with grey vertical lines. The area below the curve up to the first minimum provides an estimate of the number of water molecules coordinating to the ion. e) Representative equilibrium geometry of the [**1**·OH]^‐^ (left) and [**2**·OH]^‐^ (right) complexes at the membrane‐water interface, with first solvation shell waters shown as sticks and spheres and bulk water as transparent red sticks and the POPC membrane as spheres. Geometries and energies in a)‐c) were computed at the SMD(CHCl_3_)‐ɷB97X‐D3/TZVP//SMD(CHCl_3_)‐ɷB97X‐D3/SVP level of theory (details in Tables S3‐8). Representation: O (red), N (blue), C (grey), H (white), I (magenta). Relevant anion‐halogen bonds and anion‐hydrogen bonds are highlighted.

Chloride transport activities of the macrocyclic halogen bonding transporters **2–6** were determined using pH gradient dissipation assays in large unilamellar vesicles (LUVs). In these experiments, 1‐palmitoyl‐2‐oleoyl‐*sn*‐glycero‐3‐phosphocholine vesicles (POPC LUVs) were loaded with the pH‐responsive fluorophore 8‐hydroxypyrene‐1,3,6‐trisulfonate (HPTS) and buffered to pH 7.0 in NaCl solution. The ability of the transporter, added in an aliquot of DMSO at various concentrations, to dissipate a pH gradient generated by the addition of an NaOH base pulse was determined by recording the change in the HPTS emission with time. At the end of the experiment, the detergent Triton X‐100 was added to lyse the vesicles for calibration. For full details, see the ESI. Dose‐response curves were then fitted to the Hill equation to determine the concentration to achieve half‐maximal activity (EC_50_) (Figures 3c, S37–S42). Transport experiments were repeated in the presence of protonophore carbonyl cyanide‐p‐trifluoromethoxyphenylhydrazone (FCCP) to determine the relative rates of Cl^−^ and H^+^/OH^−^ transport by mediating rapid electrogenic H^+^ transport and, therefore, an overall coupled H^+^/Cl^−^ symport process. In the absence of FCCP, transport occurs either via Cl^−^/OH^−^ antiport or the functionally equivalent H^+^/Cl^−^ symport to maintain charge neutrality (Figure 4a, left). Proton transport is unlikely to operate in the case of these halogen bonding transporters due to a lack of any acidic protons or basic nitrogen atoms (protonated triazole p*K*_a_ <1), which would allow for reversible protonation states.^[^ ^51^ ^]^ In the case of a Cl^−^>OH^−^ selective transporter, the transport of OH^−^ ions is the rate‐determining step in the antiport transport mechanism. Upon the addition of protonophore FCCP, Cl^−^ transport becomes rate limiting, and thus the selectivity (*S*) of Cl^−^>OH^−^ of the transporter under the assay conditions can be determined from the ratio of activities (EC_50_) in the presence and absence of the protonophore.

Transporters **2**, **3,** and **4** proved to be highly active chloride transporters, with EC_50_ values for chloride transport (determined in the presence of FCCP) of 34, 68, and 50 nM, respectively. Interestingly, the enhanced chloride binding capability of **3** determined from titration experiments did not result in a higher chloride transport activity. We tentatively postulate that the larger size (1,4‐bis‐hexyl‐ vs. bis‐pentyl‐benzene linkers) may counter the enhanced binding by slowing down membrane translocation of macrocycles **3** and **5** compared to **2** and **4**. The best‐performing transporter was **2**, with an EC_50_ of 34 nM in the presence of FCCP. This represents a modest decrease in activity compared to the parent transporter **1** (3 nM with FCCP), but this is more than compensated by the observed selectivity. Macrocycles **2**–**4** exhibited low activity for OH^−^ transport (in the absence of FCCP), which prevented a quantitative measure of *S*, because low solubility at the necessary high concentrations meant that an entire dose‐response curve could not be obtained.

Therefore, we used the activity at 10 µM transporter (the solubility limit) to determine a lower bound of the selectivity, which revealed that the halogen bonding macrocycles exhibit almost two orders of magnitude higher selectivity compared to **1** (*S* of 5.3), with *S* > 300 for the most active transporter **2** (Table 1). Transporters **5** and **6** were inactive both in the presence and the absence of FCCP, despite macrocycle **5** being one of the stronger Cl^−^ receptors in solution. Compounds **5** and **6** are the most lipophilic of the family of macrocycles (clogP of 6.5 and 6.6, Table S1), which may contribute to the observed inactivity, in addition to the weak binding of chloride by **6**.

A selectivity factor for **2** of >300 in POPC lipid bilayer vesicles outperforms existing anionophores in the literature, including those based on hydrogen bonding macrocyclic cholapods^[^ ^10^ ^]^ (*S* = 100), tris‐thiourea tripods (*S* = 160),^[^ ^25^ ^]^ and halogen bonding catenane anionophores (*S* = 50). Valkenier's iodotriazole calixarene anionophore^[^ ^21^ ^]^ was reported to exhibit >100‐fold selectivity on the basis of initial rate analysis, but poor solubility required pre‐incorporation of the anionophores to the membrane during LUV preparation. Importantly, transporter **2** facilitates outstanding Cl^−^>OH^−^ selectivity while maintaining high activity and a sufficient solubility and lipophilicity profile to be delivered to pre‐formed membranes, a necessity for future application in cellular systems.

Recent studies by Gale and coworkers have demonstrated that LUVs treated with bovine serum albumin (BSA) increased the observed Cl^−^>OH^−^ selectivity because many anionophores can also facilitate H^+^ transport via catalyzing the flip‐flop of anionic fatty acids that are present in commercial lipid samples (palmitic and oleic acids) and that can be removed by sequestering with BSA.^[^ ^25^ ^]^ For example, with a neopentyl‐substituted tris‐thiourea tripodal anionophore, the observed Cl^−^>OH^−^ selectivity increased from 78 to 690 in LUVs treated with BSA to remove the majority of the fatty acids.^[^ ^10^, ^25^ ^]^ In this work, it is notable that transporter **2** maintains very high Cl^−^>OH^−^ selectivity in commercial POPC lipids containing fatty acid impurities, a property that is again critical for future biological applications in naturally occurring membranes, which feature a complex mixture of components. For means of comparison, we also determined the activity of **2** in POPC vesicles treated with BSA to remove fatty acids and observed that the activity of **2** in the absence of FCCP (i.e., for rate‐limiting hydroxide transport) decreased 4‐fold compared to that in native POPC vesicles, to the point that it is essentially inactive (Figures S43 and S44), with minimal effect on the chloride transport activity in the presence of FCCP. We therefore estimate the selectivity of **2** increases from >300 to >1000 in fatty acid‐free POPC vesicles, highlighting the exceptional intrinsic anion selectivity of the system.

To confirm that the observed pH gradient dissipation in these experiments occurs via the postulated Cl^−^ transport mechanism, we performed experiments in which the Cl^−^ ion was replaced with the bulky and hydrophilic gluconate anion, which cannot typically be transported (Figure S45). Inactivity in this assay revealed that the observed pH dissipation is not due to the possible cation‐dependent H^+^/Na^+^ antiport mechanism and confirms the integrity of the LUV membranes to nonspecific ion or HPTS leakage in the presence of the macrocycles. A mobile carrier mechanism was confirmed by performing transport experiments in 1,2‐dipalmitoyl‐*sn*‐glycero‐3‐phosphocholine (DPPC) lipid vesicles at both 25 and 45 °C, below and above the gel‐liquid phase transition temperature of 41 °C, respectively (Figure S46). Inactivity in the gel phase and restoration of transport in the liquid phase of the lipid are indicative of the expected carrier mechanism, in contrast to the phase‐independent behavior typically observed for membrane‐spanning channels.^[^ ^52^ ^]^

### 2.4 Binding and Transport Simulations

To rationalize the difference in ion selectivity between acyclic **1** and its cyclic variants **2**–**4**, we computed the binding energies of Cl⁻ / OH⁻ ions to **1**–**4** at the SMD(CHCl3)‐ωB97X‐D3/def2‐QZVP// SMD(CHCl3)‐ωB97X‐D3/def2‐SVP level of theory^[^ ^46^, ^47^, ^48^ ^]^. Chloroform was used to mimic the membrane dielectric in line with our previous work^[^ ^16^, ^17^ ^]^ (see the methods section and §7 Supporting Information for details).^[^ ^53^ ^]^ While such an implicit solvation model does not account for desolvation effects, it allows us to compare how binding preferences vary with transporter structure (Figure 4a for compounds **1**–**2**, Figures S56‐S57 for **3**–**4**).

As anticipated, iodine‐anion distances are larger (by an average of 0.6 Å) for the Cl^−^ complexes than in OH^−^ complexes, which is consistent with the higher charge density of the latter. The calculated binding energy differences (Figure 4b and 4c) show that all transporters preferentially bind OH^−^ over Cl‐; however, this preference decreases from the acyclic **1** (ΔE = ‐14.3 kcal mol^−1^) to the macrocycles **2** and **4** (ΔE = ‐12.4 and ‐12.6 kcal mol^−1^) and to a lesser extent for the larger macrocycle **3** (ΔE = ‐13.1 kcal mol^−1^). The binding energies can be further broken down into a distortion component, representing the energy required to adopt the bound conformation, and an interaction component, which reflects the ion‐transporter interactions.^[^ ^54^ ^]^ This analysis shows that interaction energy, rather than distortion, drives the changes in OH^−^ binding preference. Analysis of the free energy of binding shows a more marked decrease in OH^‐^ binding preference when comparing acyclic and cyclic transporters (Figure 4c). This arises from both a reduced enthalpic preference of OH^−^ binding, from **1** (‐6.7 kcal mol^−1^) to **2**, **4** (‐4.0 and ‐4.1 kcal mol^−1^) and **3** (‐5.1 kcal mol^−1^) and to a lesser extent entropic contributions, leading to an overall reduction of over 3 kcal mol^−1^ for OH⁻ binding preference in the cyclic transporters.

To evaluate the differences in ion solvation between acyclic transporter **1** and cyclic transporter **2**, we performed equilibrium (3×100 ns) MD simulations of the transporters in complex with Cl^−^ and OH^−^ at the membrane interface. Harmonic restraints (2000 kJ mol⁻¹ nm⁻^2^) were applied between the iodines and the anion's heavy atom at the DFT‐optimized distance. Membrane simulations followed established protocols (see §8 Supporting Information for details), using POPC bilayers with the Stockholm lipid parameters in the TIP3P‐FB model for water.^[^ ^55^, ^56^ ^]^

The oxygen ion RDFs, (Figure 4d and S59‐S60) represent the time‐averaged distance distribution between the oxygen of water and the Cl^−^ or OH⁻ oxygen atom. Integrating the area under the curve yields the number of water molecules in the first solvation shell.^[^ ^57^, ^58^ ^]^ This analysis shows that macrocycle **2** reduces ion solvation, which is reflected in a lower RDF maxima of both the Cl^−^ (g(r)Cl‐O max = 3.4 Å) and OH^−^ (g(r)O‐O max = 2.9 Å) and the consequent reduction in the number of waters in the first hydration shell. For Cl^−^ water coordination decreases from 2.1 in 1 to 1.7 in **2** (a 19% decrease), while for OH‐ it decreases from 2.6 to 1.9 (a 27% reduction). The increased desolvation of OH^−^ in **2**, coupled with the higher hydration enthalpy of hydroxide,^[^ ^27^, ^28^ ^]^ will therefore disfavor OH⁻ binding to **2** at the membrane interface (Figure 4e) to a greater extent than for chloride, thereby suppressing hydroxide anion transport.

## 3 Conclusions

We report a family of novel macrocyclic anion transporters exhibiting exceptional chloride over proton/hydroxide ion transport activity. This is achieved by combining halogen bonding anion binding with anion encapsulation provided by a macrocyclic ionophore structure. By merging these two concepts, we achieve a remarkable chloride‐over‐hydroxide selectivity of over 300‐fold while maintaining excellent overall activity and the ability to deliver such anionophores to pre‐formed lipid vesicles. In vesicles treated to remove fatty acid impurities, which provide an alternative proton transport pathway, the intrinsic selectivity is estimated to exceed 1000‐fold. The short and convergent synthetic procedure utilizing click chemistry renders these scaffolds an attractive access point for studying chloride transport in biological systems, as well as allowing for future optimization of physicochemical properties. Computational studies in implicit solvent revealed an increasing energetic cost for hydroxide over chloride in the macrocycle, resulting in a reduction in relative binding preference for OH^−^ observed in the acyclic structure. Furthermore, MD simulations in POPC membrane in explicit solvent enabled the evaluation of the binding‐induced desolvation of the anions at the membrane interface. This analysis revealed that anion encapsulation at the binding site reduces the solvation around both ions and consequently increases the dehydration penalty, with the effect being more pronounced for hydroxide. The reduction in hydroxide binding preference and enhanced desolvation effects induced by the halogen bonding macrocyclic structure together contribute to the overall high chloride over hydroxide selectivity. We anticipate that the novel halogen‐bonding macrocyclic scaffolds, along with the computational rationalizations of their high selectivity, may inform the development of future highly selective transporters for translational applications in medicine.

## Supporting information

The authors have cited additional references within the Supporting Information.

## Author Contributions

M.F., F.D. and M.J.L conceived the project. M. F. carried out the synthesis, analytical and computational studies. M.J.L. and F.D. supervised and directed the research. The manuscript was written by all the authors.

## Conflict of Interest

The authors declare no conflict of interest.

## Supporting information



Supporting Information

## Data Availability

The data that support the findings of this study are available in the supplementary information of this article.
