# Supplementary material for: Chloride Selective, Nonprotonophoric Ion Transport with Macrocyclic Halogen Bonding Anionophores
Source: Chemistry. 2025 Jun 26;31(43):e202502033. doi: 10.1002/chem.202502033 (PMC12319357; doi:10.1002/chem.202502033)
Supplement: Supplementary file 1 — Supporting Information [file CHEM-31-e202502033-s001.pdf]

Chloride Selective, Non-protonophoric Ion Transport with Macrocyclic Halogen Bonding Anionophores

Martin Flerin, Fernanda Duarte and Matthew J. Langton

Table of Contents

1. Materials and Methods 2
2. Synthesis and Characterisation 3
3. Anion Transport Experiments 29
   Vesicle Preparation 29
   Transport Assays with HPTS 29
   HPTS Assay Data for all Transporters 30
   Sodium Gluconate Assay Data for all Transporters 33
   Membrane Fluidity Studies 34
4. NMR Titration Experiments 34
5. Predicted cLogP Values of Transporters 40
6. Product Ratios in Macrocyclisation of 4 and 5 40
7. DFT Calculations 41
   Energy details of Calculations 43
   Minimum Energy Structures of Transporters and Their Complexes 49
8. Molecular Dynamics 51
   Metadynamics in DMSO 52
   Membrane Simulations 55
   Water Simulations 55
   RDFs of Water Around Ions In the Membrane and in Solution 56

1. Materials and Methods

All reagents and solvents were purchased from commercial sources and used without further purification. Lipids were purchased from Avanti polar lipids and used without further purification. Where necessary, solvents were dried by passing through an MBraun MPSP-800 column and degassed with nitrogen. Triethylamine was distilled from and stored over potassium hydroxide. Normal phase silica gel flash column chromatography was performed either manually using Merck® silica gel 60 under a positive pressure of nitrogen or on a Buchi Pure C-815 Flash automated column chromatography system using FlashPure EcoFlex silica cartridges. Where mixtures of solvents were used, ratios are reported by volume. NMR spectra were recorded on a Bruker AVIIIHD 400 Nanobay and Bruker NEO 600 spectrometers. Chemical shifts are reported as δ values in ppm. Mass spectra were carried out on a Waters RDa bench-top TOF used with an Acquity LC system for reverse-phased chromatography. Fluorescence spectroscopic data were recorded using an Agilent Cary Eclipse fluorescence spectrophotometer, equipped with a Peltier temperature controller and stirrer. Experiments were conducted at 25 °C unless otherwise stated. Vesicles were prepared as described below using an Avestin “LiposoFast” extruder apparatus, equipped with polycarbonate membranes with 200 nm pores. GPC purification of vesicles was carried out using GE Healthcare PD-10 desalting columns prepacked with Sephadex G 25 medium.

Abbreviations:

DCM: Dichloromethane CuAAC: Copper(I)-catalyzed azide-alkyne cycloaddition; DCM: Dichloromethane; DMF: N,N- Dimethylformamide; DMSO: Dimethylsulfoxide; DPPC: 1,2-dipalmitoyl-sn-glycero-3- phosphocholine; EC50: Effective concentration; EDTA: Ethylenediaminetetraacetic acid; EtOAc: Ethyl acetate; FCCP: Carbonyl cyanide-p-trifluoromethoxyphenylhydrazone; HEPES: N-(2- hydroxyethyl)piperazine-N’-(2-ethanesulfonic acid); HPTS: 8-hydroxy-1,3,6- pyrenetrisulfonate; KOH: Potassium hydroxide; LUVs: large unilamellar vesicles; MeCN: Acetonitrile; MeOH: Methanol; NaCl: Sodium chloride; POPC: 1-palmitoyl-2-oleoyl-sn-glycero-3-phosphocholine; rt: Room temperature; TBA salt: Tetrabutylammonium salt; TBTA: Tris((1-benzyl-4-triazolyl)methyl)amine; THF: Tetrahydrofuran; XB: Halogen Bonding; DFT: Density Functional Theory; MD: Molecular Dynamics; MM: Molecular Mechanics.

2. Synthesis and Characterisation

Warning! Low molecular weight organic azides used in this study are potentially explosive and should be used on a small scale. Appropriate protective measures should always be taken when handling these compounds.

Tris[(1-benzyl-1H-1,2,3-triazol-4-yl)methyl]amine (TBTA)[1] and diethynylnitrobenzene[2] were prepared according to literature procedures.

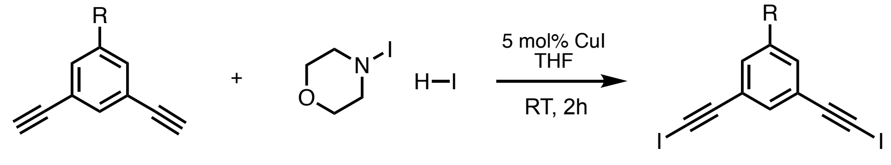

R = NO_2 / COOMe

General Procedure 1. Di-iodoethynyl-arenes were prepared in accordance with literature precedent.[3,4] The requisite di-ethynyl-arene (0.29 mmol, 1 eq.) was dissolved in dry THF (10 mL) at room temperature. CuI (55 mg, 0.29 mmol, 1 eq.) was added, and the mixture was left to stir for 5 minutes. Following this, N-iodomorpholine (325 mg, 0.957 mmol, 3.3 eq.) was added portionwise, and the reaction was left to stir at room temperature overnight. The reaction mixture was diluted with DCM (30 mL) and washed with saturated $Na_2S_2O_3$ (cca. 20 mL), until the reddish colour had mostly disappeared from the organic phase. The aqueous layer was back-extracted with DCM (1 x 20 mL) and the combined organics were dried over $MgSO_4$ and concentrated to afford the respective di-iodoethynyl-arene.

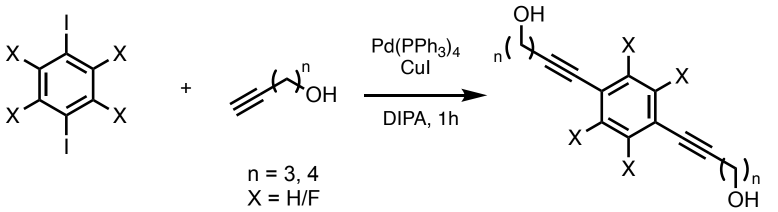

General Procedure 2. Bis-1,4-alkynol arenes were prepared from the corresponding p-substituted diiodoarene by a double Sonogashira reaction adapted from literature precedent.[5] The diiodoarene (1 mmol, 1 eq.) was dissolved in DIPA (10 mL). Pd(PPh3)4 (42 mg, 5 mol%) and CuI (11 mg, 5 mol%) were added and the solution was degassed. The alkynol (2.2 eq.) was then added and the reaction mixture was heated to 90° C for approximately 45 minutes, after which significant decomposition was observed if the reaction was left at this temperature for a longer time. The reaction mixture was diluted with DCM (20 mL) and filtered through a pad of Celite, washed with more DCM (3 x 30 mL) and adsorbed onto silica. Flash column chromatography (3:2, EtOAc:Pentane) afforded the respective products.

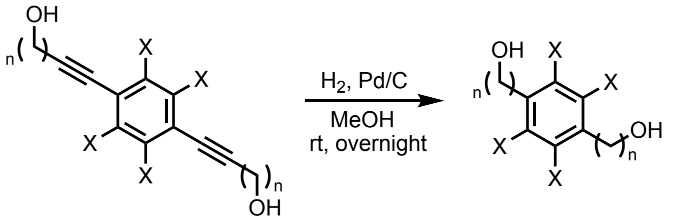

n = 3, 4
X = H/F

n = 5, 6
X = H/F

General Procedure 3. Bis-1,4-alkanol arenes were prepared from the corresponding p-substituted bis-1,4-alkynols prepared using General Procedure 2 by standard catalytic hydrogenation. The bis-1,4-alkynol (1 mmol, 1 eq.) was dissolved in MeOH (20 mL), and the 10% Pd/C (50 mg, 5 mol%) was added as a suspension in deionised water (0.5 mL). The vessel was filled with hydrogen with one balloon and a second balloon was used to establish a hydrogen atmosphere overnight at room temperature, following which the reaction mixture was filtered through a pad of silica, dried over $MgSO_4$, and concentrated to afford the product.

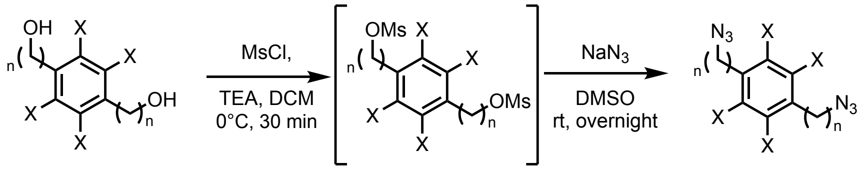

n = 5, 6
X = H/F

General Procedure 4. Bis-1,4-(azidoalkyl)arenes were prepared from the corresponding p-substituted bis- 1,4-alkanols by standard tosylation and S_N2 displacement by an azide. The bis-1,4-alkanol (1 mmol, 1 eq.) and MsCl (0.47 mL, 2.4 mmol, 2.4 eq.) were dissolved in DCM (20 mL), at 0°C, and NEt_3 (2 mL) was added dropwise. The reaction was allowed to warm to room temperature and left until monitoring by TLC indicated full completion. It was then diluted with DCM (50 mL) and washed with water (50 mL). The aqueous phase was washed with DCM (2 x 50 mL), and the combined organics were dried over $MgSO_4$ and concentrated to afford the crude intermediate, which was immediately redissolved in DMSO (5 mL), to which NaN_3 (155 mg, 2.2 mmol, 2.2 eq.) was added portion-wise and the mixture was left to react overnight. The reaction was quenched with brine (20 mL), and back-extracted with Et_2O (5x 25 mL). The combined organic layers were once again washed with brine (100 mL), dried over $MgSO_4$ and concentrated to yield the bis-azide.

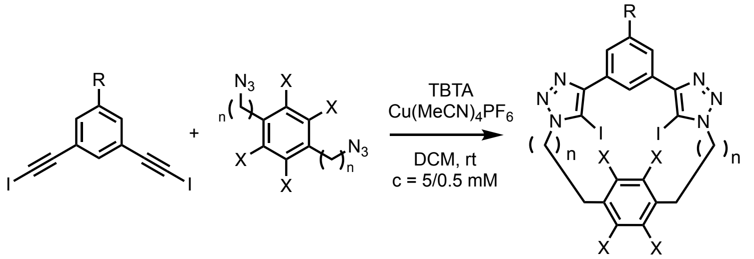

n = 5, 6
X = H/F

n = 4, 5
X=H/F

General Procedure 5. Macrocycles with alkyl linkers were prepared using standard iodoalkyne azide click reaction conditions at varying concentrations (5/0.5 mM).[3,4,6] $Cu(MeCN)_4PF_6$ (9 mg, 50 mol%) and TBTA (13 mg, 50 mol%) were dissolved in dry DCM (100 or 10 mL) and stirred for 30 minutes in order to precomplex. Di-iodoethynyl arene (0.05/0.005 mmol, 1 eq.) and bis-azide (0.05/0.005 mmol, 1 eq.) were added, and the reaction was left to stir in the dark for 48 hours or until complete conversion. The reaction mixture was washed with EDTA/NH_4OH solution, dried over $MgSO_4$, adsorbed onto silica and purified with flash column chromatography in 2% MeOH in DCM to give a mixture of [1+1] and [2+2] macrocycles. This mixture was adsorbed onto silica

once more and subjected to reverse-phase flash column chromatography in 60:40 to 100:0 MeCN:Water mixtures to give the desired [1+1] macrocycle as the first elution band, which was then further purified by recrystalisation from a hot 1:4 mixture of EtOAc:heptane to afford the pure [1+1] macrocycle.

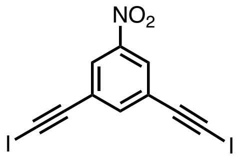

Known compound 1,3-bis(iodoethynyl)-5-nitrobenzene **7** was prepared according to General Procedure 1 and literature precedent[3,4] as a pale yellow solid (108 mg, 0.26 mmol, 88%).

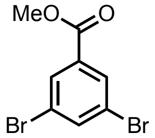

Literature compound[7] methyl 3,5-dibromobenzoate **8** was prepared from 3,5-dibromobenzoic acid (279 mg, 1 mmol, 1 eq.) suspended in dry DCM under a nitrogen atmosphere at 0 °C. A catalytic amount of DMF was added to the reaction mixture first, followed by oxalyl chloride (0.686 mL, 8 mmol, 8 eq.) being added dropwise. All volatiles were removed, and MeOH was immediately added to the dry acid chloride intermediate. After 30 minutes, the reaction mixture was concentrated to afford the yellow crystalline product (280 mg, 0.96 mmol, 96%)

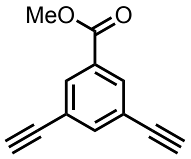

Known compound methyl 3,5-diethynylbenzoate **9** was synthesised according to an adapted literature procedure.[8] Compound **8** (0.292 g, 1 mmol, 1 eq.) was dissolved in a mixture of toluene and diisopropylamine (10 mL : 3 mL) and degassed. $Pd(PPh_3)Cl_2$ (0.35 g, 5 mol%), CuI (10 mg, 5 mol%) and TMS-acetylene (0.55 mL, 4 eq.) were added, and the reaction mixture was heated to 80 °C overnight. The crude mixture was filtered through celite, which was washed with DCM (3 x 30 mL) and the combined organic phase adsorbed onto silica. Column chromatography using 10% DCM in hexane afforded the TMS-protected product, which was immediately dissolved in methanol (5 mL). To this solution, KOH in 3 mL of a MeOH:water mixture (2:1) was added dropwise. After 30 minutes, the mixture was diluted with DCM (50 mL), which was washed with water to remove inorganic salts, dried, and concentrated to a brown solid (342 mg, 2 mmol, 70%).

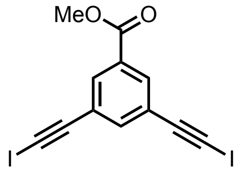

Methyl 3,5-bis(iodoethynyl)benzoate **10** was prepared according to General Procedure 1 as an off-white solid (55%). ^{1}H NMR (400 MHz, CDCl3) δ 8.03 (d, J = 1.6 Hz, 2H), 7.64 (t, J = 1.6 Hz, ^{1}H), 3.91 (s, 3H). ^{13}C NMR (101 MHz, CDCl3) δ 165.6, 139.8, 133.6, 130.8, 124.3, 92.3, 52.7, 9.5. $[C_{12}H_{7}I_{2}O_{2}]^{+}$ calculated 436.8530 ; found 436.8525.

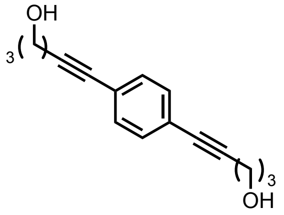

5,5'-(1,4-phenylene)bis(pent-4-yn-1-ol) **11** was prepared according to General Procedure 2 as a white solid (80%). ^{1}H NMR (400 MHz, CDCl3) δ 7.29 (s, 4H), 3.81 (t, J = 6.1 Hz, 4H), 2.52 (t, J = 6.9 Hz, 4H), 1.89 – 1.82 (m, 4H). ^{13}C NMR (101 MHz, CDCl3) δ 131.5, 123.2, 91.1, 81.0, 61.9, 31.5, 16.2. $[C_{16}H_{19}O_{2}]^{+}$ calculated 243.1380, found 243.1384

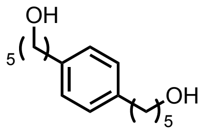

5,5'-(1,4-phenylene)bis(pentan-ol) **12** was prepared according to General Procedure 3 as a white solid (80%). ^{1}H NMR (400 MHz, CDCl3) δ 7.08 (s, 4H), 3.63 (t, J = 6.6 Hz, 4H), 2.58 (t, J = 7.7 Hz, 4H), 1.71 – 1.54 (m, 8H), 1.43 – 1.36 (m, 4H). ^{13}C NMR (101 MHz, CDCl3) δ 140.0, 128.4, 63.1, 35.6, 32.8, 31.4, 25.5. $[C_{16}H_{27}O_{2}]^{+}$ calculated 251.2006, found 251.2018

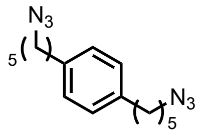

5,5'-(1,4-phenylene)bis(5-azidopentyl) **13** was prepared according to General Procedure 4 as a thick orange oil (80%). ^{1}H NMR (400 MHz, CDCl3) δ 7.09 (s, 4H), 3.26 (t, J = 6.9 Hz, 4H), 2.59 (t, J = 7.5 Hz, 4H), 1.68 – 1.60 (m, 8H), 1.45 – 1.38 (m, 4H). ^{13}C NMR (101 MHz, CDCl3) δ 139.8, 128.5, 51.6, 35.5, 31.2, 28.9, 26.5. $[C_{16}H_{24}N_{6}K]^{+}$ calculated 339.1694, found 339.1670.

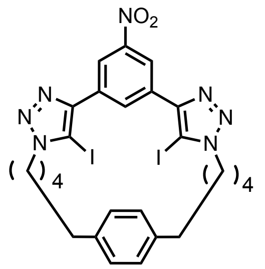

Macrocycle **2** was prepared according to General Procedure 5 as an off-white solid (33%). ^{1}H NMR (400 MHz, CDCl3) δ 8.71 (d, J = 1.6 Hz, 2H), 8.41 (t, J = 1.6 Hz, ^{1}H), 6.75 (s, 4H), 4.66 – 4.59 (m, 4H), 2.34 – 2.26 (m, 4H), 2.17 – 2.07 (m, 4H), 1.47 (p, J = 6.9 Hz, 4H), 1.00 (p, J = 7.9 Hz, 4H).

^{13}C NMR (101 MHz, CDCl3) δ 148.6, 139.4, 134.5, 132.8, 128.3, 122.5, 51.9, 35.1, 30.5, 28.3, 26.3. $[C_{26}H_{28}I_2N_7O_2]^+$ calculated 724.0389; found 724.0388

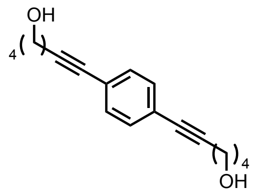

5,5'-(1,4-phenylene)bis(hex-5-yn-1-ol) **14** was prepared according to General Procedure 2 as a white solid (75%). ^1H NMR (400 MHz, CDCl3) δ 7.26 (s, 4H), 3.67 (q, J = 5.6 Hz, 4H), 2.42 (t, J = 6.6 Hz, 4H), 1.80 – 1.62 (m, 8H). ^{13}C NMR (101 MHz, CDCl3) δ 131.5, 123.2, 91.5, 80.9, 62.6, 32.0, 25.1, 19.4. $[C_{18}H_{23}O_2]^+$ calculated 271.1693, found 271.1700

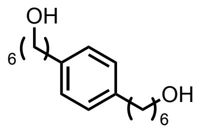

5,5'-(1,4-phenylene)bis(hexan-1-ol) **15** was prepared according to General Procedure 3 as a white solid (95%). ^1H NMR (400 MHz, CDCl3) δ 7.08 (s, 4H), 3.63 (t, J = 6.6 Hz, 4H), 2.61 – 2.53 (m, 4H), 1.66 – 1.51 (m, 8H), 1.41 – 1.33 (m, 8H). ^{13}C NMR (101 MHz, CDCl3) δ 139.9, 128.3, 63.0, 35.5, 32.7, 31.5, 25.6. $[C_{18}H_{31}O_2]^+$ calculated 279.2319, found 279.2352

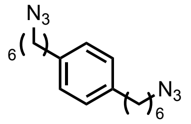

5,5'-(1,4-phenylene)bis(azidohexanyl) **16** was prepared according to General Procedure 4 as light yellow oil (65%). ^1H NMR (400 MHz, CDCl3) δ 7.06 (d, J = 1.8 Hz, 4H), 4.19 (td, J = 6.5, 2.5 Hz, 4H), 2.56 (td, J = 8.0, 1.9 Hz, 4H), 1.79 – 1.67 (m, 4H), 1.67 – 1.54 (m, 4H), 1.45 – 1.30 (m, 8H). ^{13}C NMR (101 MHz, CDCl3) δ 139.8, 128.3, 51.5, 35.4, 31.4, 28.8, 28.8, 26.6. $[C_{18}H_{29}N_6]^+$ calculated 351.2268, found 351.2264

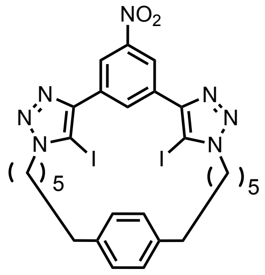

Macrocycle **3** was prepared according to General Procedure 5 as an off-white solid (42%). ^1H NMR (400 MHz, CDCl3) δ 8.79 (d, J = 1.5 Hz, 2H), 8.68 (t, J = 1.5 Hz, ^1H), 6.99 (s, 4H), 4.50 (t, J = 6.4 Hz, 4H), 2.51 – 2.43 (m, 4H), 1.95 (p, J = 6.9 Hz, 4H), 1.59 – 1.48 (m, 4H), 1.39 – 1.31 (m, 4H), 1.30 – 1.22 (m, 4H). ^{13}C NMR (101 MHz, CDCl3) δ 149.3, 140.2, 132.5, 131.9, 128.9, 122.5, 52.7, 50.6, 34.9, 31.3, 29.3, 27.5, 25.0. $[C_{28}H_{32}I_2N_7O_2]^+$ calculated 752.0702; found 752.0699

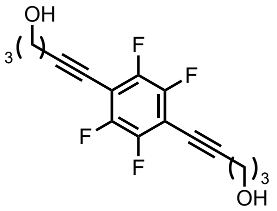

5,5'-(perfluoro-1,4-phenylene)bis(pentyn-1-ol) **17** was prepared according to General Procedure 2 as an off- white solid (63%). ^{1}H NMR (400 MHz, CDCl3) δ 3.87 – 3.79 (m, 4H), 2.66 (t, J = 6.9 Hz, 4H), 1.90 (d, J = 12.9 Hz, 4H). ^{13}C NMR (151 MHz, CDCl3) δ 148.0 – 146.1 (m), 104.6, 104.3, 66.9 – 66.7 (m), 61.5, 31.0, 16.6. $[C_{16}H_{15}F_{4}O_{2}]^{+}$ calculated 315.1003, found 315.1015

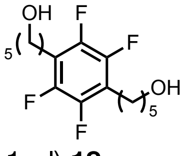

5,5'-(perfluoro-1,4-phenylene)bis(pentan-1-ol) **18** was prepared according to General Procedure 3 as a white solid (95%). ^{1}H NMR (600 MHz, CDCl3) δ 3.64 (q, J = 6.1 Hz, 4H), 2.70 (t, J = 7.6 Hz, 4H), 1.66 – 1.55 (m, 8H), 1.41 (m, 6H, CH_2 + OH). ^{13}C NMR (151 MHz, CDCl3) δ 145.8 - 143.8 (m), 118.2 – 117.8 (m), 62.9, 32.5, 29.2, 25.5, 22.7. $[C_{16}H_{23}F_{4}O_{2}]^{+}$ calculated 323.1629, found 323.1640

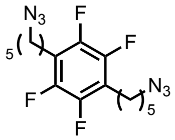

1,4-bis(5-azidopentyl)-2,3,5,6-tetrafluorobenzene **19** was prepared according to General Procedure 4 as a lightly coloured oil (80%). ^{1}H NMR (600 MHz, CDCl3) δ 3.27 (t, J = 6.9 Hz, 4H), 2.71 (t, J = 7.6 Hz, 4H), 1.68 – 1.59 (m, 8H), 1.47 – 1.39 (m, 4H). ^{13}C NMR (151 MHz, CDCl3) δ 145.8 – 143.4 (m), 117.9 – 117.7 (m), 51.4, 28.9, 28.7, 26.4, 22.6. $[C_{16}H_{21}F_{4}N_{6}\text{-Na}]^{+}$ calculated 395.1578, found 395.1590.

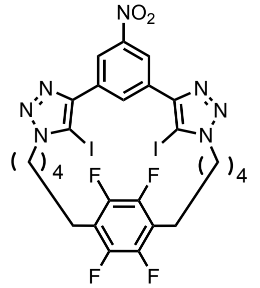

Macrocycle **4** was prepared according to General Procedure 5 as an off-white solid (30%). ^{1}H NMR (600 MHz, CDCl3) δ 8.72 (d, J = 1.6 Hz, 2H), 8.33 (t, J = 1.6 Hz, 1H), 4.65 – 4.60 (m, 4H), 2.42 (t, J = 7.5 Hz, 4H), 2.10 (p, J = 6.6 Hz, 4H), 1.48 – 1.40 (m, 4H), 1.15 – 1.07 (m, 4H). ^{13}C NMR (151 MHz, CDCl3) δ 148.9, 148.8, 145.5 – 143.3 (m), 134.9, 132.8, 122.4, 117.5, 78.4, 51.5, 28.6, 28.4, 25.9, 22.1. $[C_{26}H_{24}F_{4}I_{2}N_{7}O_{2}]^{+}$ calculated 796.0012, found 795.9975

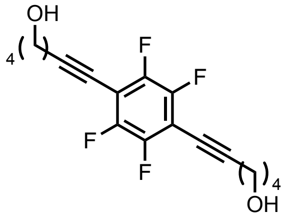

5,5’-(perfluoro-1,4-phenylene)bis(hex-5-yn-1-ol) **20** was prepared according to General Procedure 2 as a very light pink-white solid (50%). ^{1}H NMR (600 MHz, CDCl3) δ 3.64 (q, J = 6.1 Hz, 4H), 2.70 (t, J = 7.6 Hz, 4H), 1.61 (dt, J = 13.6, 7.0 Hz, 8H), 1.48 – 1.36 (m, 8H). ^{13}C NMR (151 MHz, CDCl3) δ 147.8 – 145.9 (m), 104.6, 104.6 – 104.2 (m), 68.4 – 64.7 (m), 62.3, 31.7, 24.5, 19.7. $[C_{18}H_{19}F_4O_2]^+$ calculated 343.1316, found 343.1337

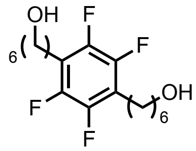

5,5’-(perfluoro-1,4-phenylene)bis(hexan-1-ol) **21** was prepared according to General Procedure 3 as a white solid (90%). ^{1}H NMR (600 MHz, CDCl3) δ 3.64 (t, J = 6.6 Hz, 4H), 2.69 (t, J = 7.7 Hz, 4H), 1.63 – 1.54 (m, 8H), 1.45 – 1.33 (m, 8H). ^{13}C NMR (151 MHz, CDCl3) δ 145.6 – 143.9 (m), 118.3 – 117.9 (m), 63.1, 32.8, 29.4, 29.1, 25.5, 22.7. $[C_{18}H_{27}F_4O_2]^+$ calculated 351.1942, found 351.1917

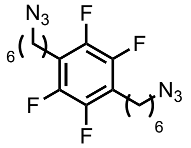

1,4-bis(6-azido)-2,3,5,6-tetrafluorobenzene **22** was prepared according to General Procedure 4 as light yellow oil (87%). ^{1}H NMR (600MHz,CDCl3) δ 3.26 (t, J = 7.0 Hz, 4H), 2.70 (t, J = 7.7 Hz, 4H), 1.60 (p, J= 7.7 Hz, 8H), 1.45 – 1.33 (m, 8H). ^{13}C NMR (151 MHz, CDCl3) δ 146.0 – 144.0 (m), 118.7 – 117.2 (m), 51.5, 29.2, 28.9, 28.8, 26.5, 22.7. $[C_{18}H_{24}F_4N_6Na]^+$ calculated 423.1891, found 423.1884

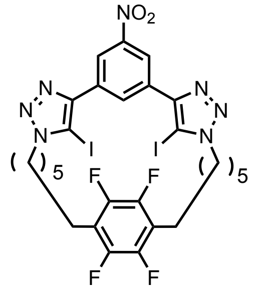

Macrocycle **5** was prepared according to General Procedure 5 as an off-white solid (38%). ^{1}H NMR (600 MHz, CDCl3) δ 8.78 (d, J = 1.6 Hz, 2H), 8.63 (t, J = 1.6 Hz, ^{1}H), 4.59 – 4.54 (m, 4H), 2.57 (t, J = 7.3 Hz, 4H), 1.98 – 1.91 (m, 4H), 1.54 – 1.49 (m, 3H), 1.32 – 1.27 (m, 8H). ^{13}C NMR (151 MHz, CDCl3) δ 149.2, 148.8, 145.7 – 143.7 (m), 133.3, 132.7, 122.8, 117.9, 77.6, 50.9, 29.7, 28.7, 27.5, 24.9, 21.6. $[C_{28}H_{28}F_4I_2N_7O_2]^+$ calculated 824.0325, found 824.0324

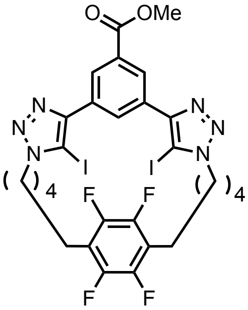

Macrocycle **6** was prepared according to General Procedure 5 as an off-white solid (32%). ^{1}H NMR (400 MHz, CDCl3) δ 8.50 (d, *J* = 1.8 Hz, 2H), 8.09 (t, *J* = 1.7 Hz, 1H), 4.64 – 4.57 (m, 4H), 3.98 (s, 3H), 2.42 (t, *J* = 7.5 Hz, 4H), 2.08 (p, *J* = 6.6 Hz, 4H), 1.43 (p, *J* = 7.1 Hz, 4H), 1.15 (p, *J* = 7.6 Hz, 4H). ^{13}C NMR (126 MHz, CDCl3) 166.6, 133.6, 131.5, 131.1, 129.1, 78.1, 52.5, 51.4, 28.6, 28.5, 25.9, 22.1. $[C_{28}H_{27}F_4I_2N_6O_2]^+$ calculated 809.0216; found 809.0220.

^{1}H and ^{13}C NMR Spectra of Novel Compounds

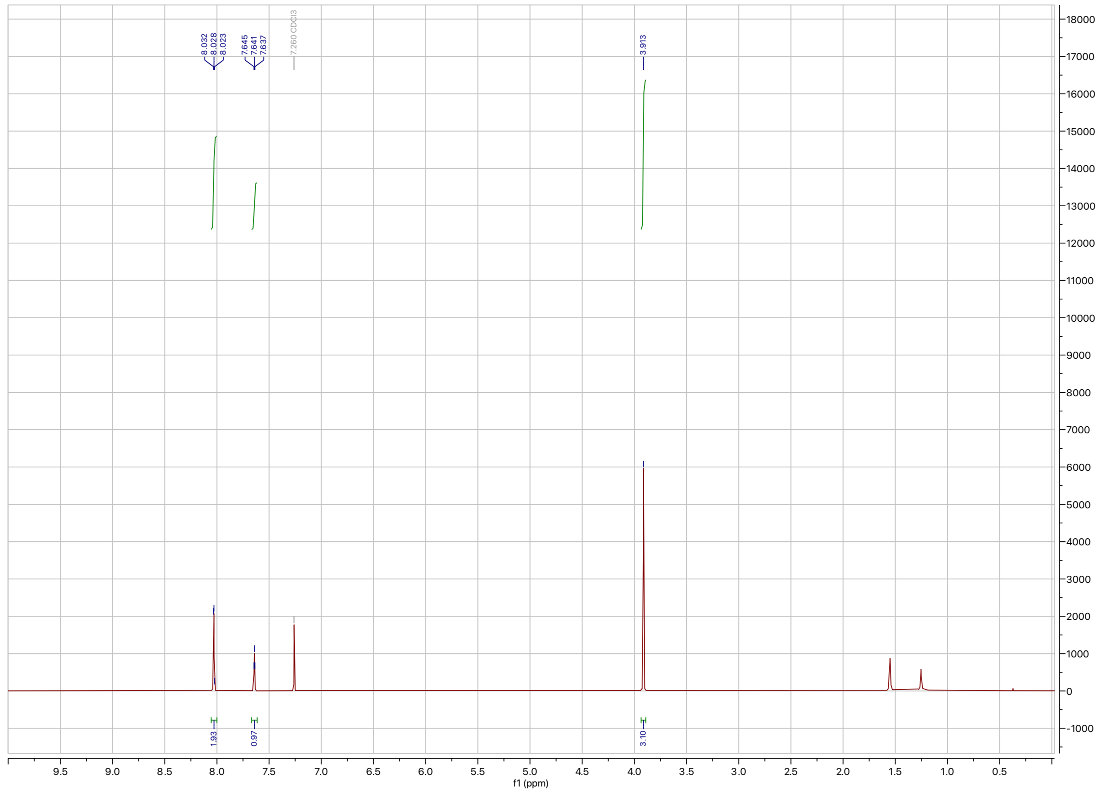

Figure S1. ^{1}H NMR spectrum of compound **10**. (CDCl₃, 298K)

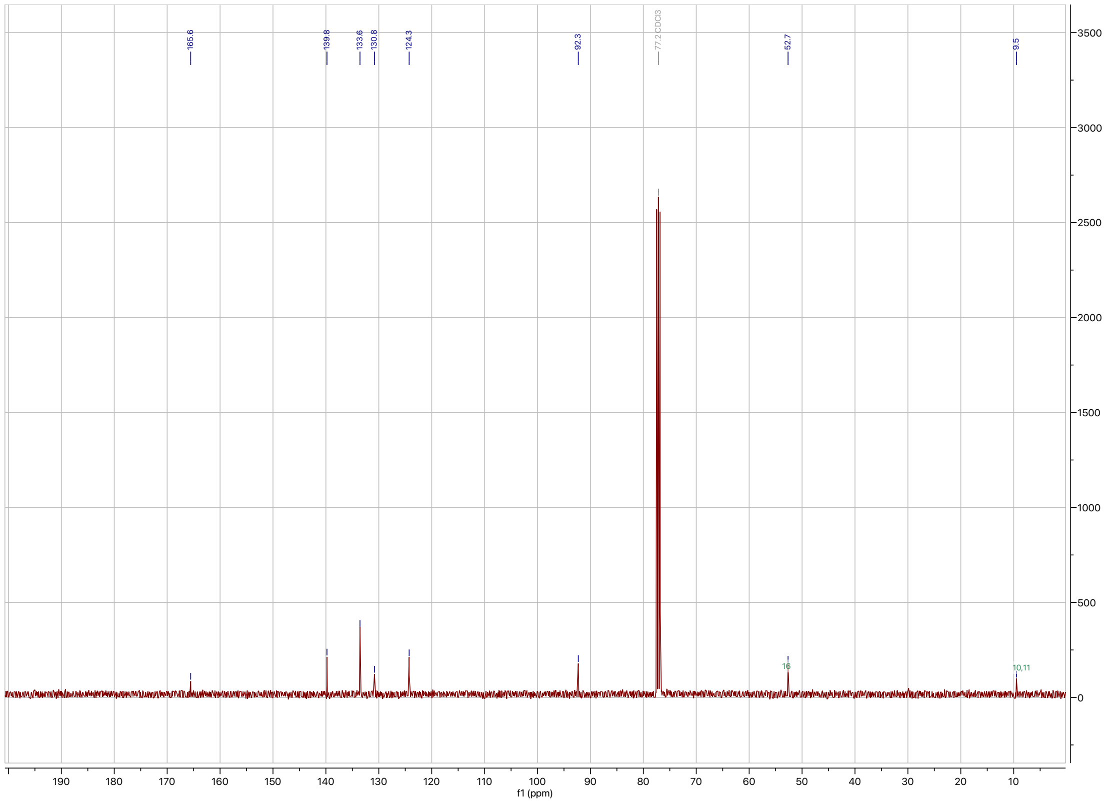

Figure S2. ^{13}C NMR spectrum of compound **10.** ($CDCl_3$, 298K)

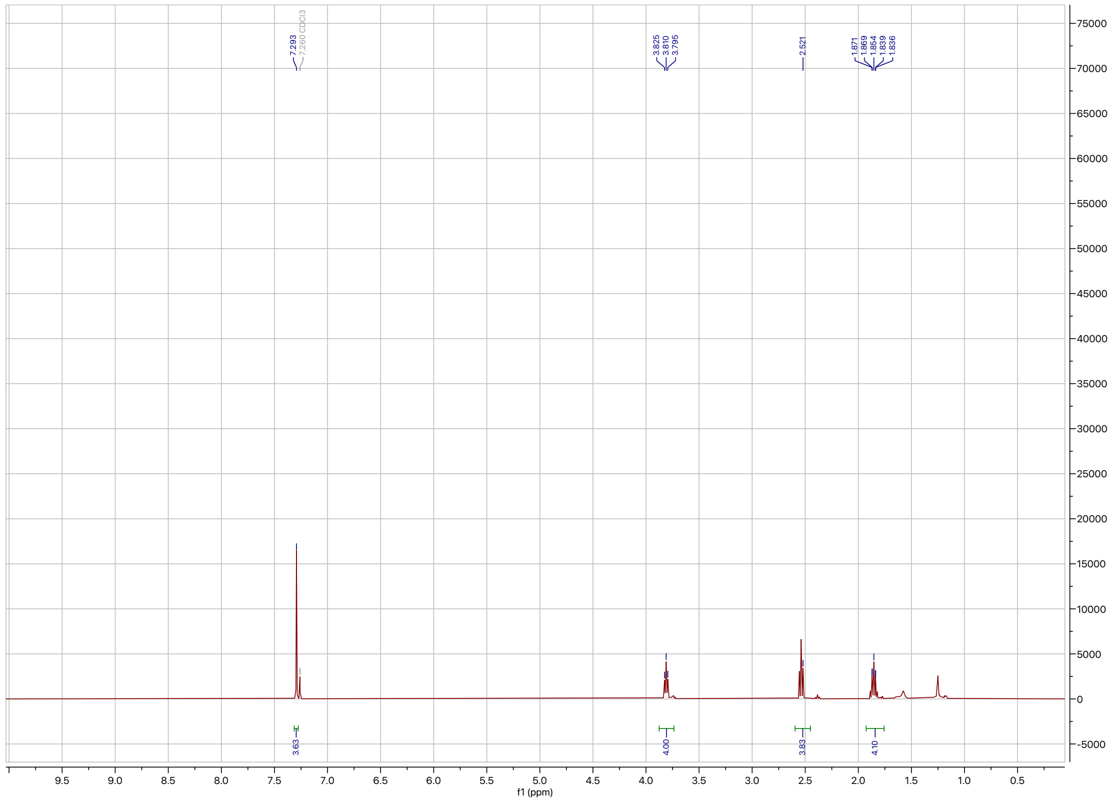

Figure S3. ^{1}H NMR spectrum of compound **11.** ($CDCl_3$, 298K)

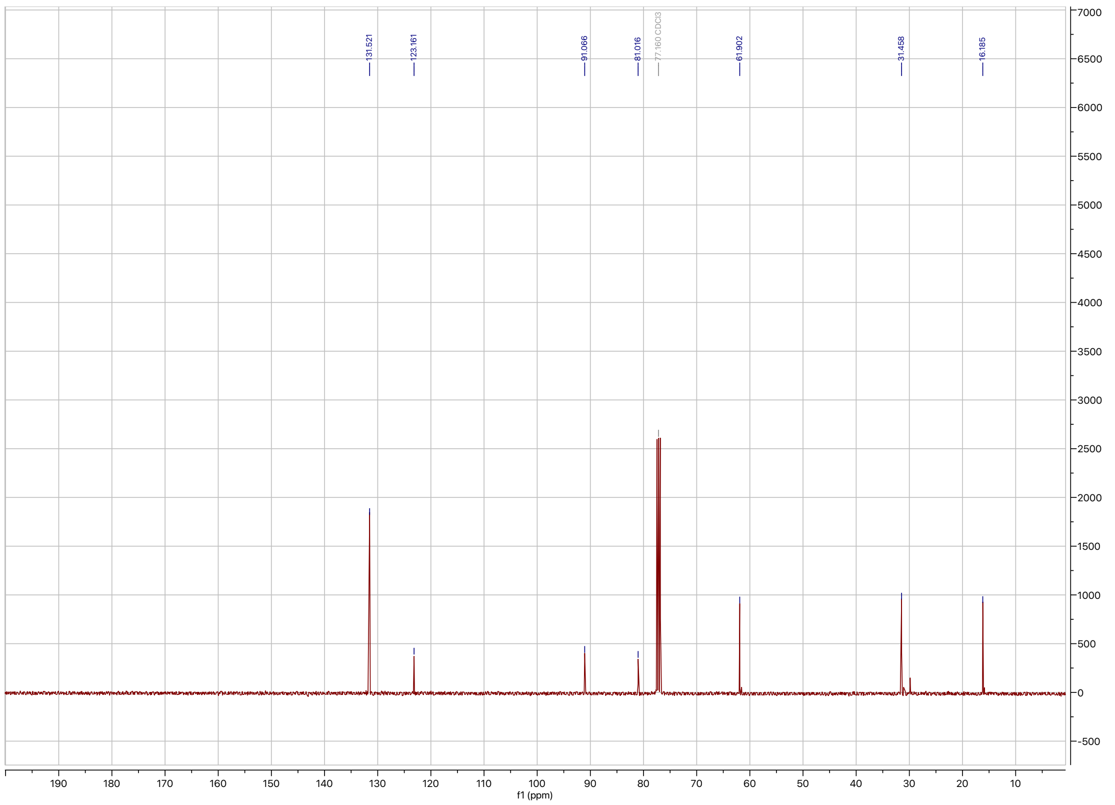

Figure S4. ^{13}C NMR spectrum of compound **11.** ($CDCl_3$, 298K)

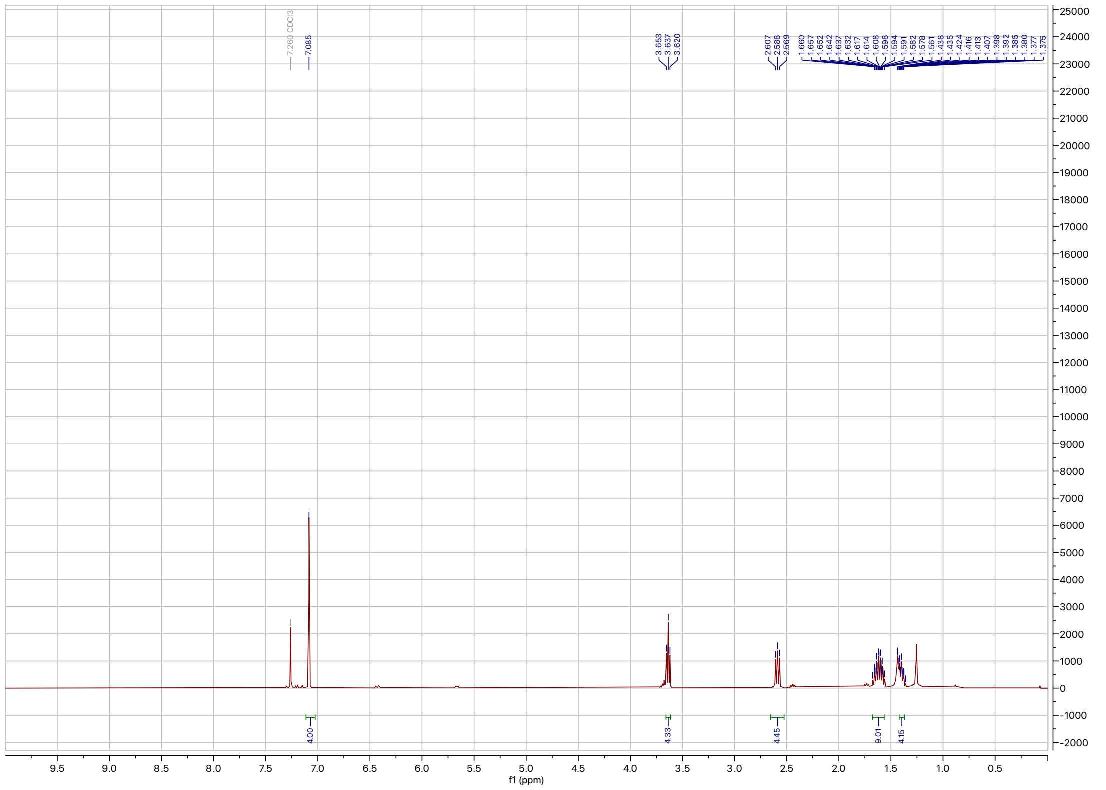

Figure S5. ^{1}H NMR spectrum of compound **12.** ($CDCl_3$, 298K)

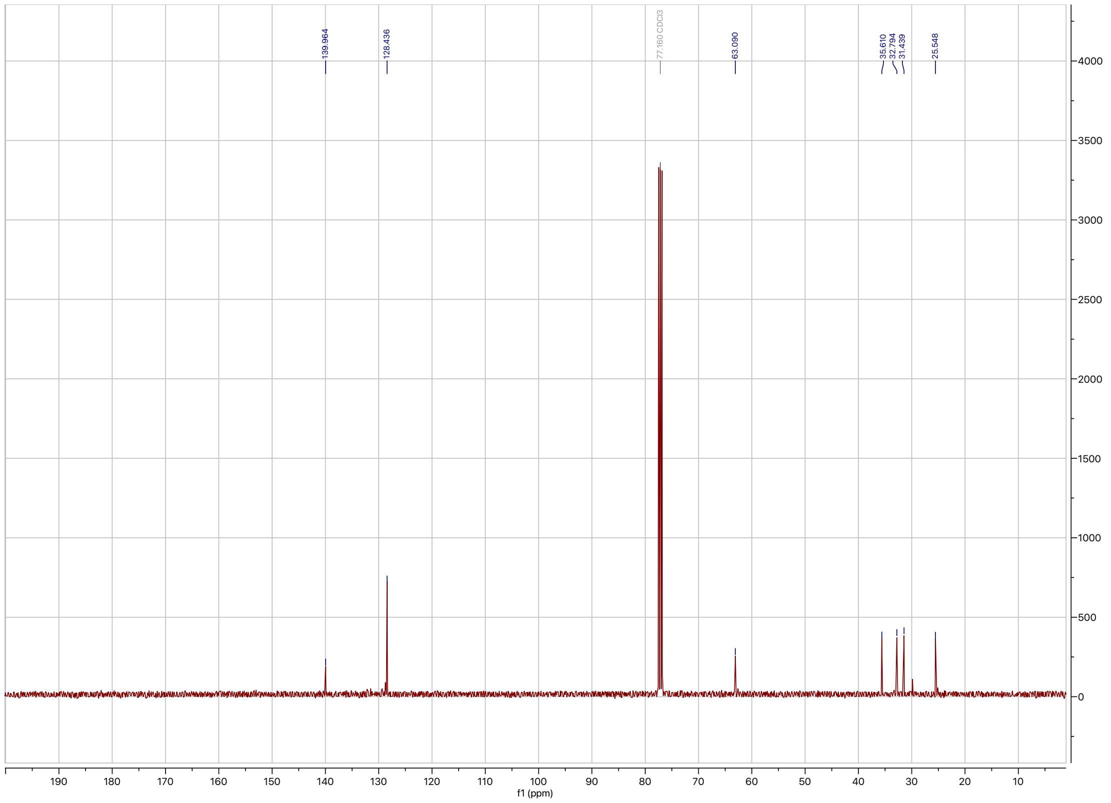

Figure S6. ^{13}C NMR spectrum of compound **12.** ($CDCl_3$, 298K)

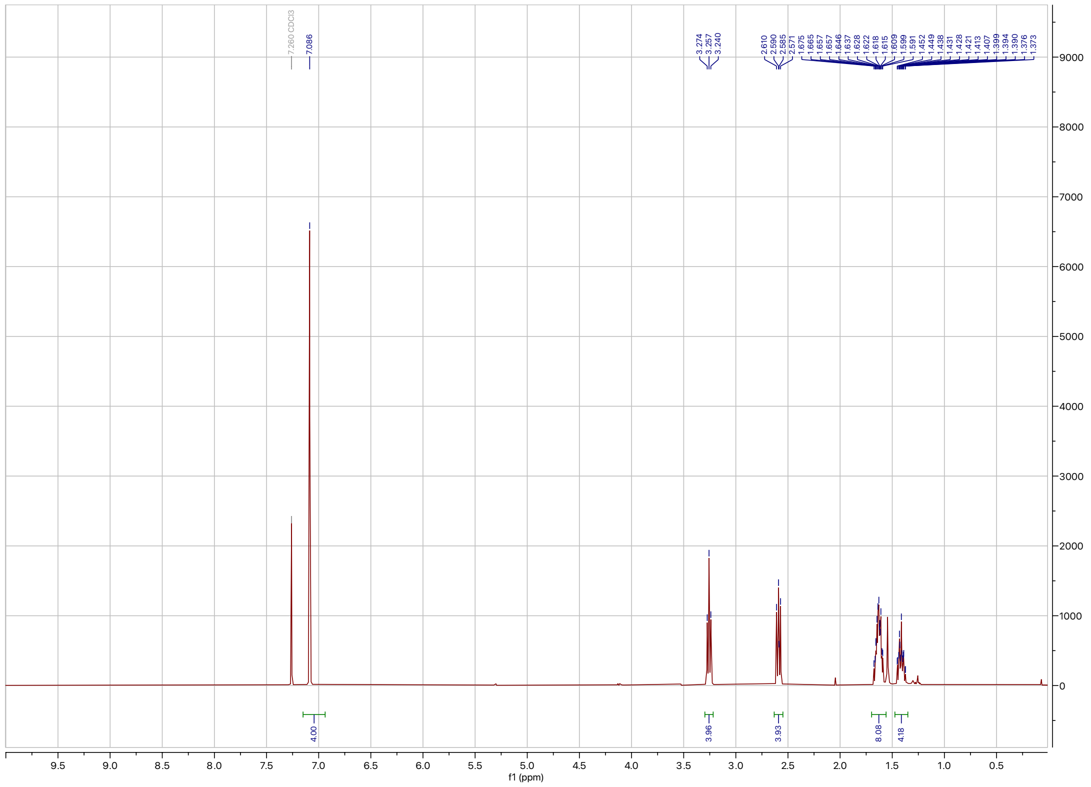

Figure S7. ^{1}H NMR spectrum of compound **13.** ($CDCl_3$, 298K)

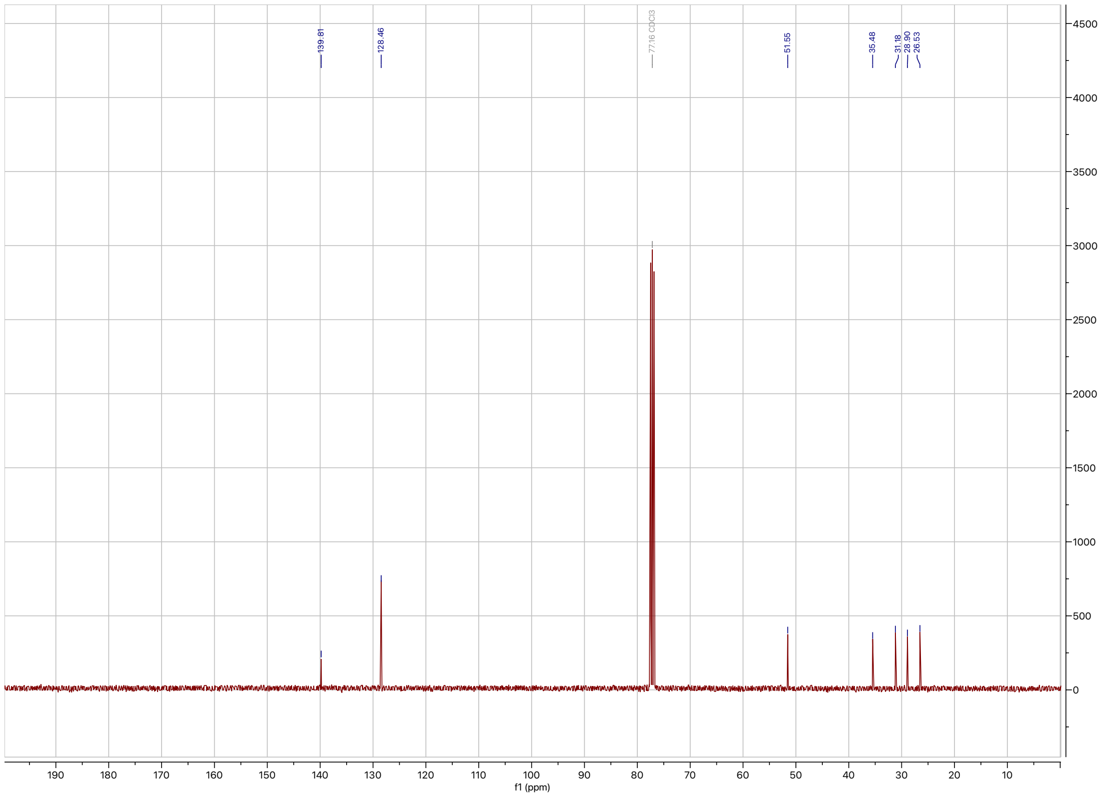

Figure S8. ^{13}C NMR spectrum of compound **13.** ($CDCl_3$, 298K)

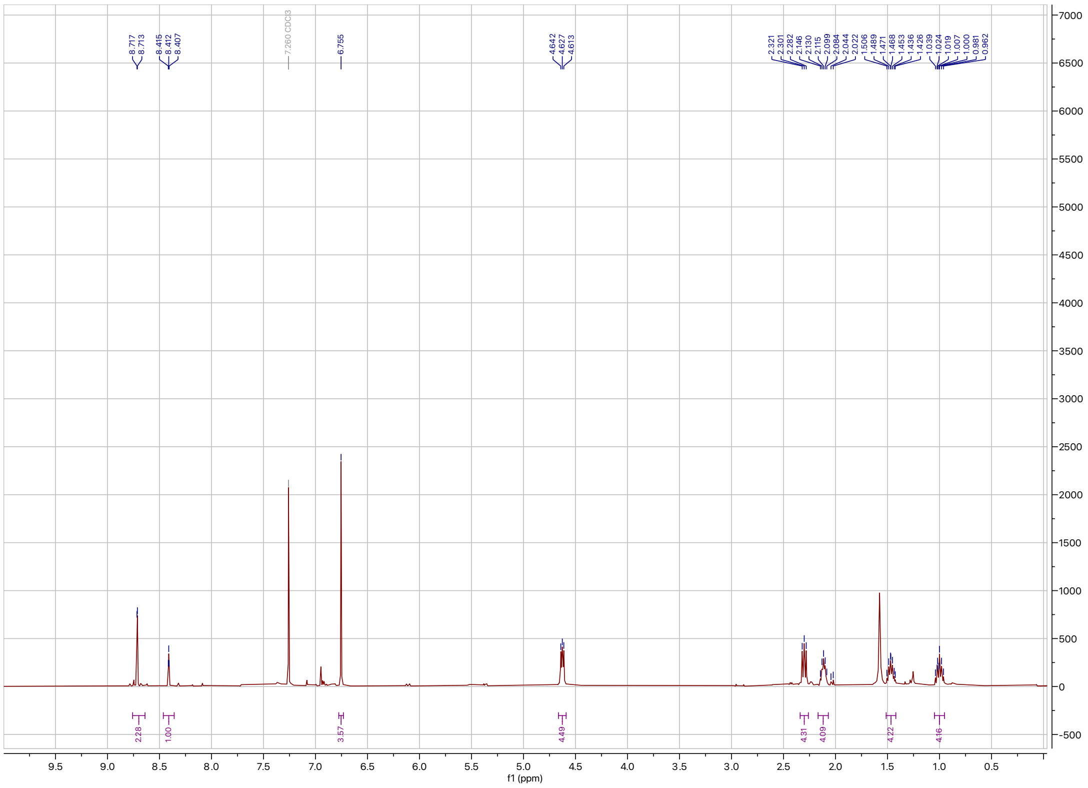

Figure S9. ^{1}H NMR spectrum of compound **2.** ($CDCl_3$, 298K)

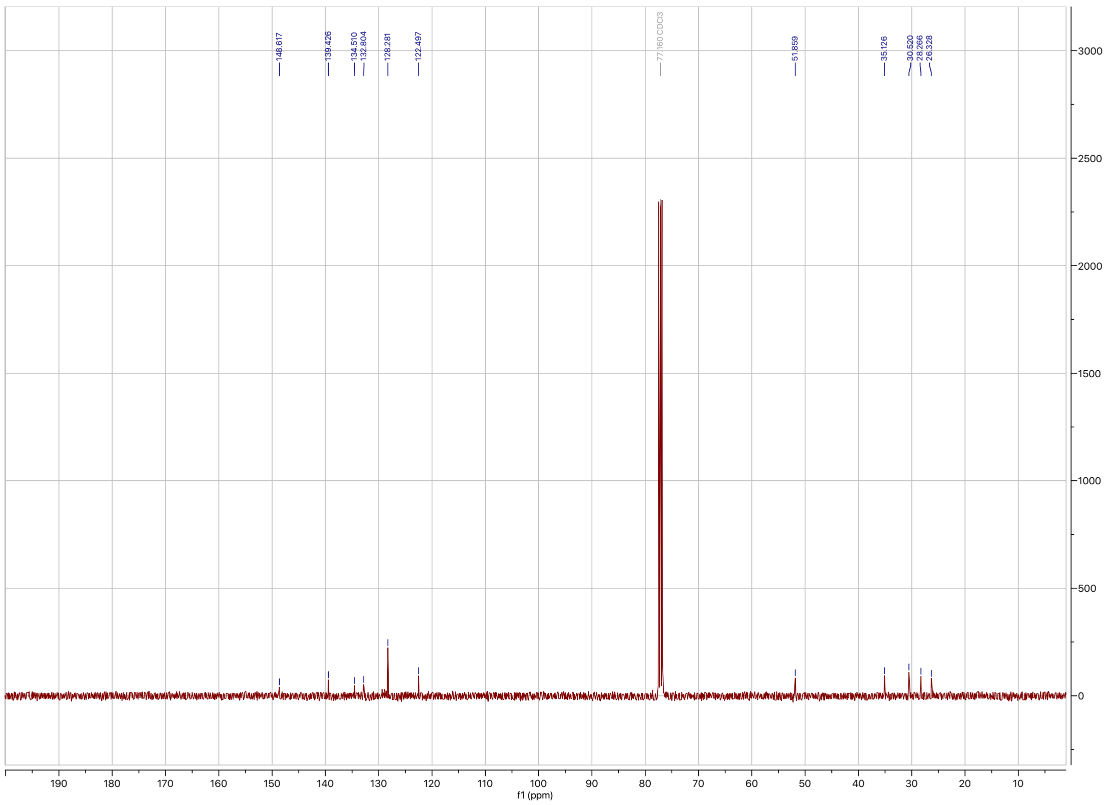

Figure S10. ^{13}C NMR spectrum of compound **2.** ($CDCl_3$, 298K)

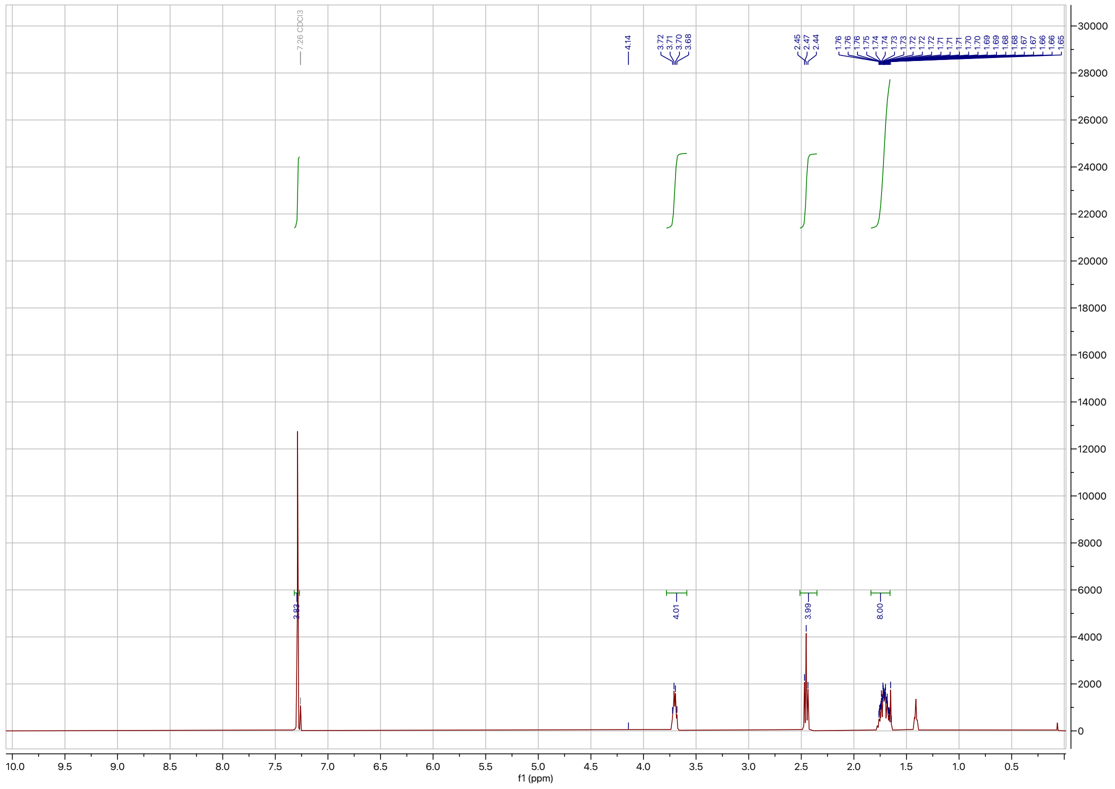

Figure S11. ^{1}H NMR spectrum of compound **14.** ($CDCl_3$, 298K)

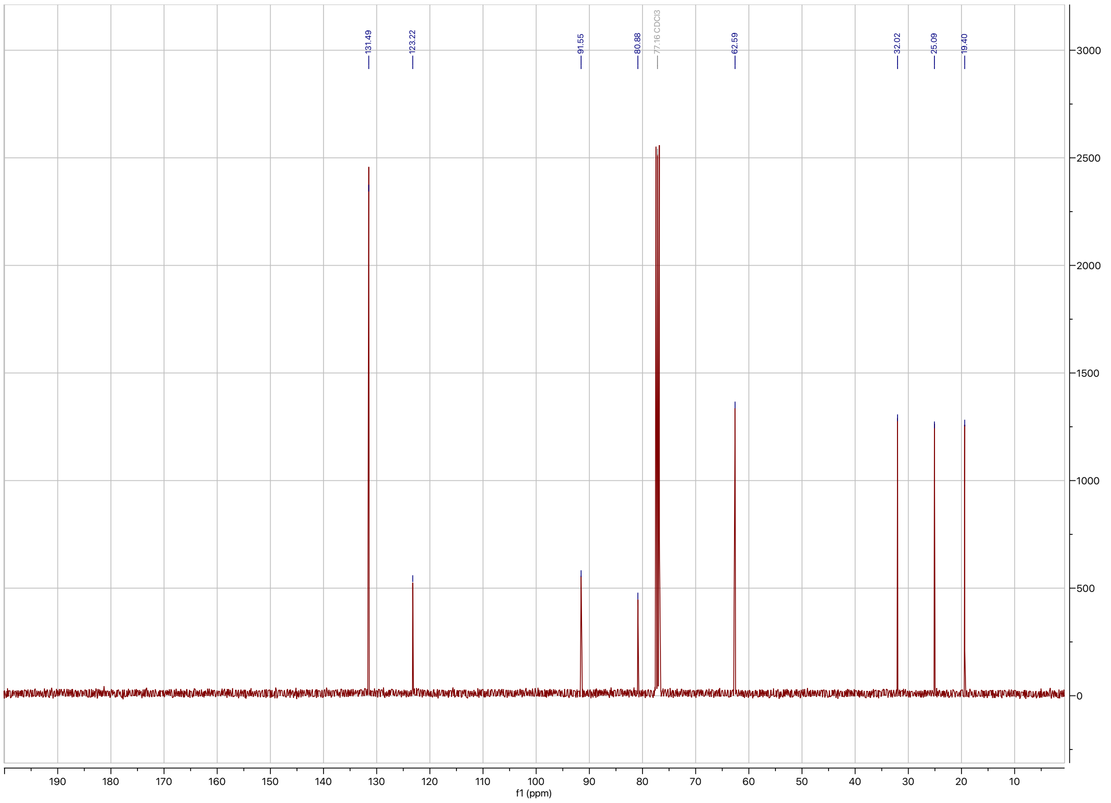

Figure S12. ^{13}C NMR spectrum of compound **14.** ($CDCl_3$, 298K)

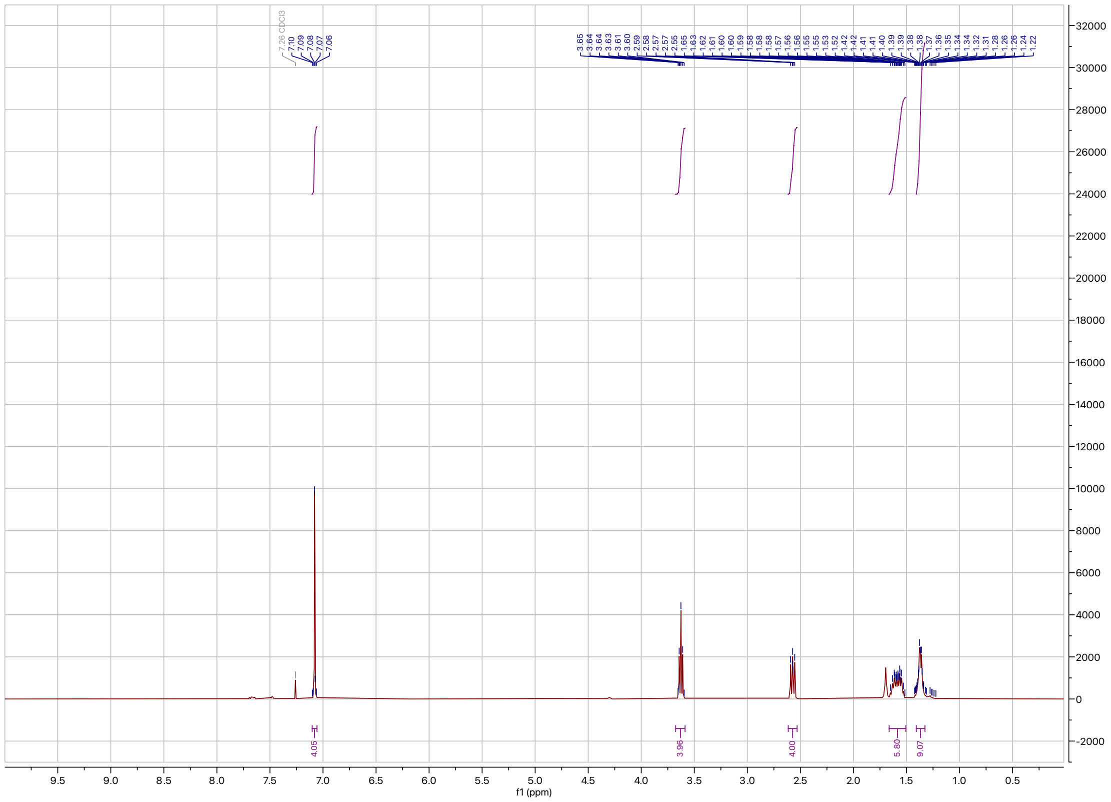

Figure S13. ^{1}H NMR spectrum of compound **15.** ($CDCl_3$, 298K)

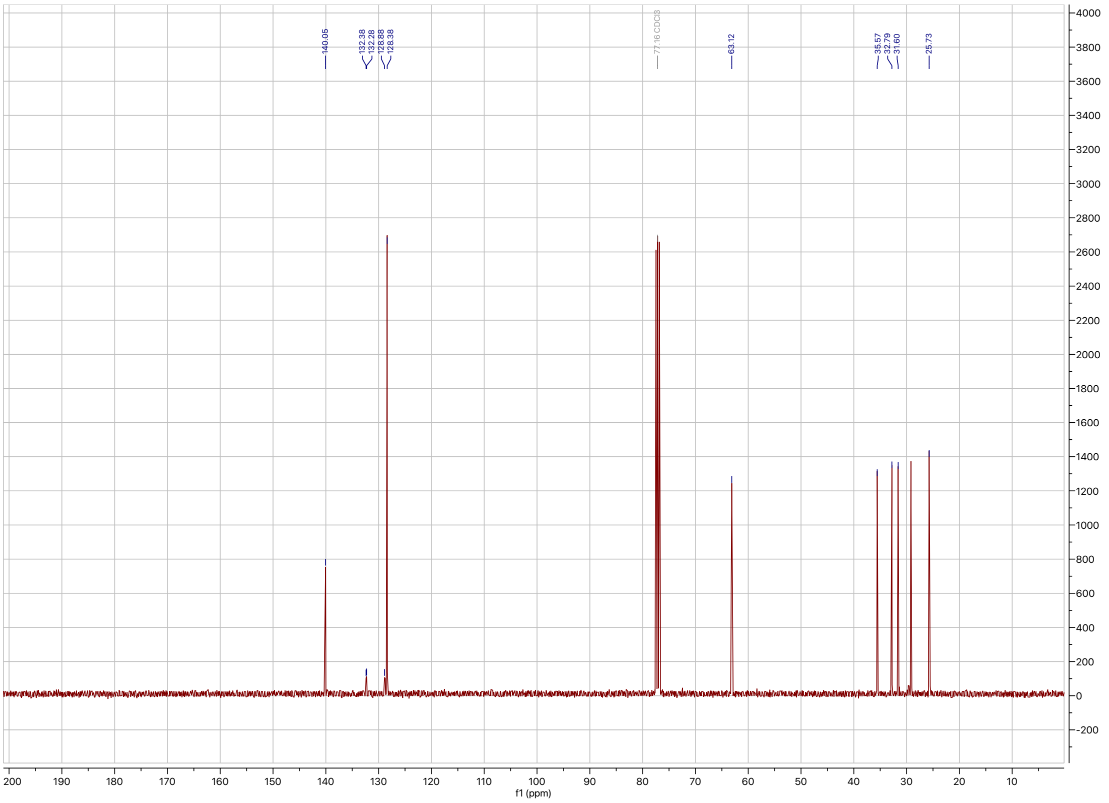

Figure S14. ^{13}C NMR spectrum of compound **15.** ($CDCl_3$, 298K)

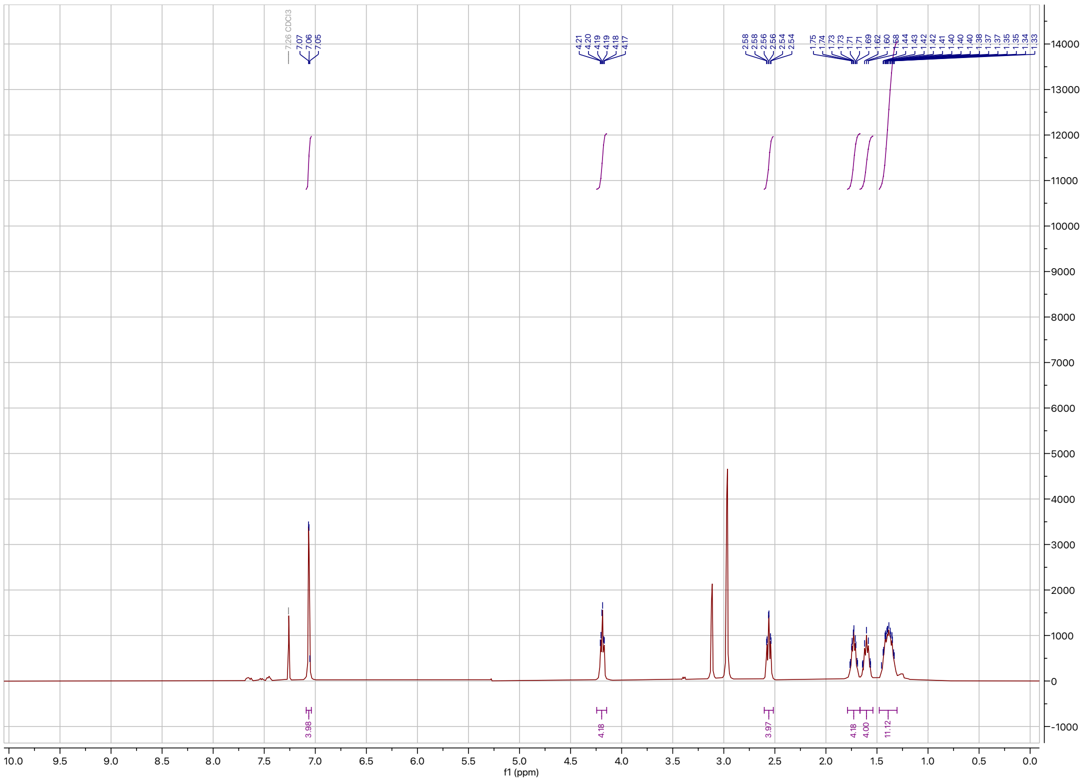

Figure S15. ^{1}H NMR spectrum of compound **16.** ($CDCl_3$, 298K)

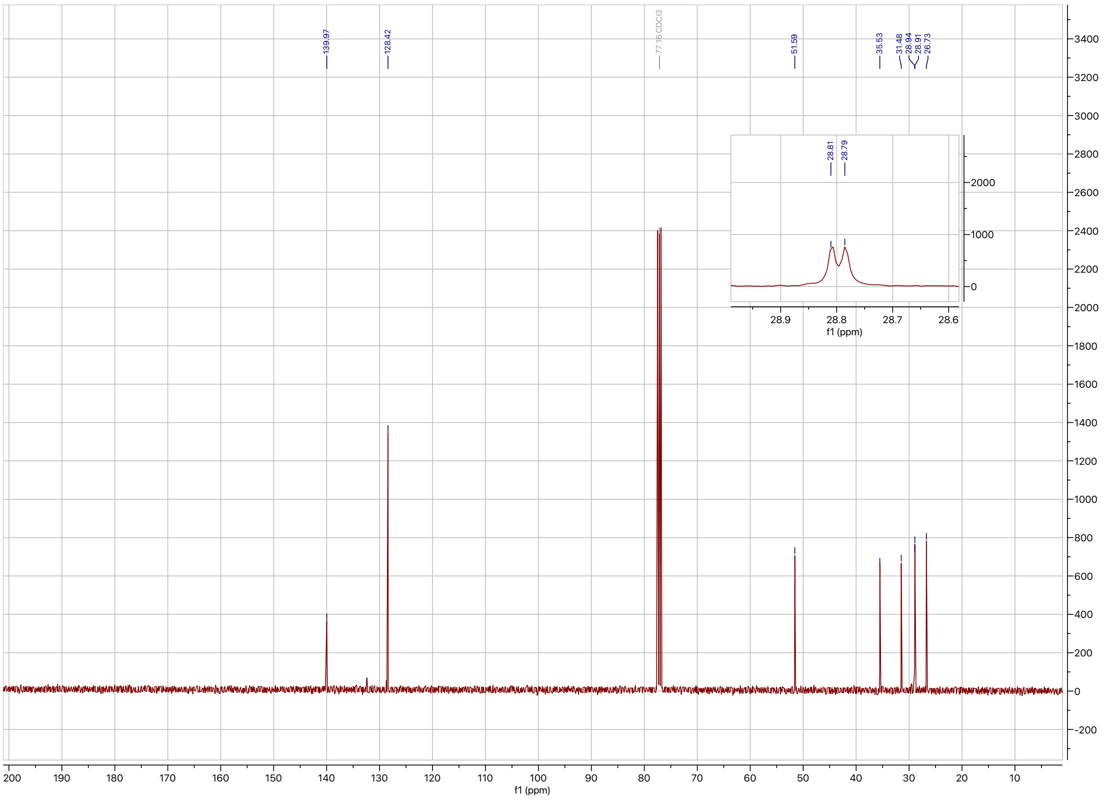

Figure S16. ^{13}C NMR spectrum of compound **16.** ($CDCl_3$, 298K)

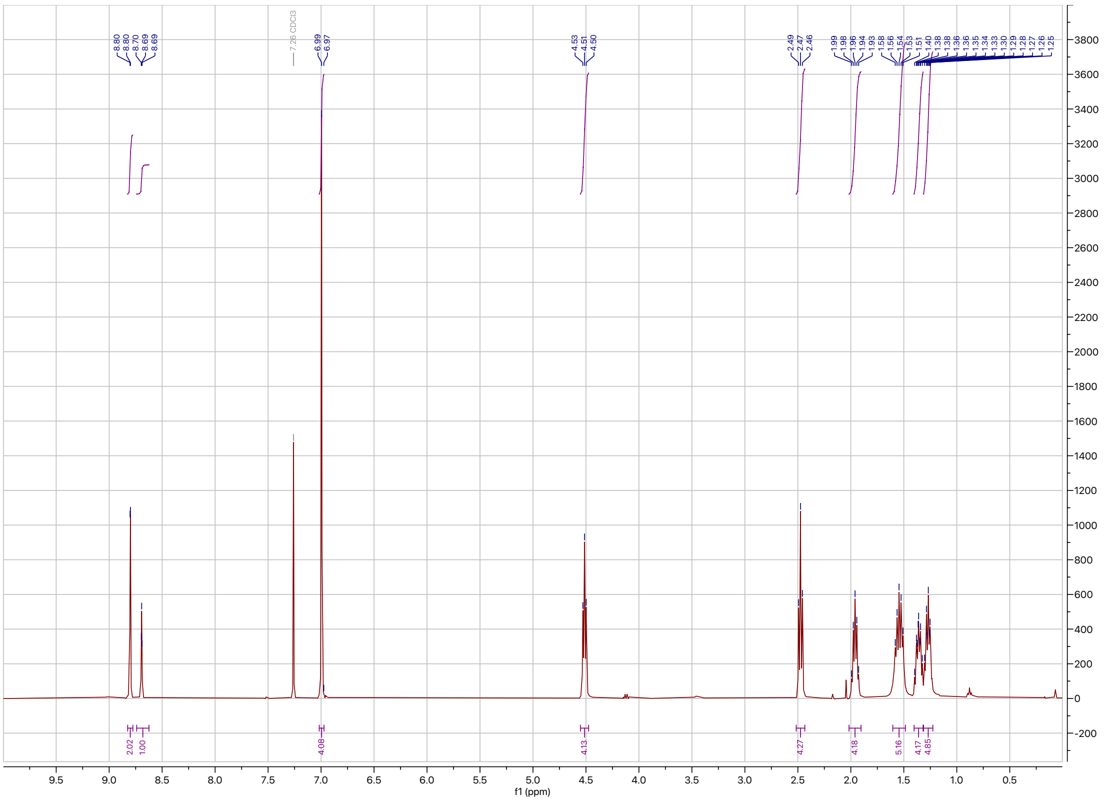

Figure S17. ^{1}H NMR spectrum of compound **3.** ($CDCl_3$, 298K)

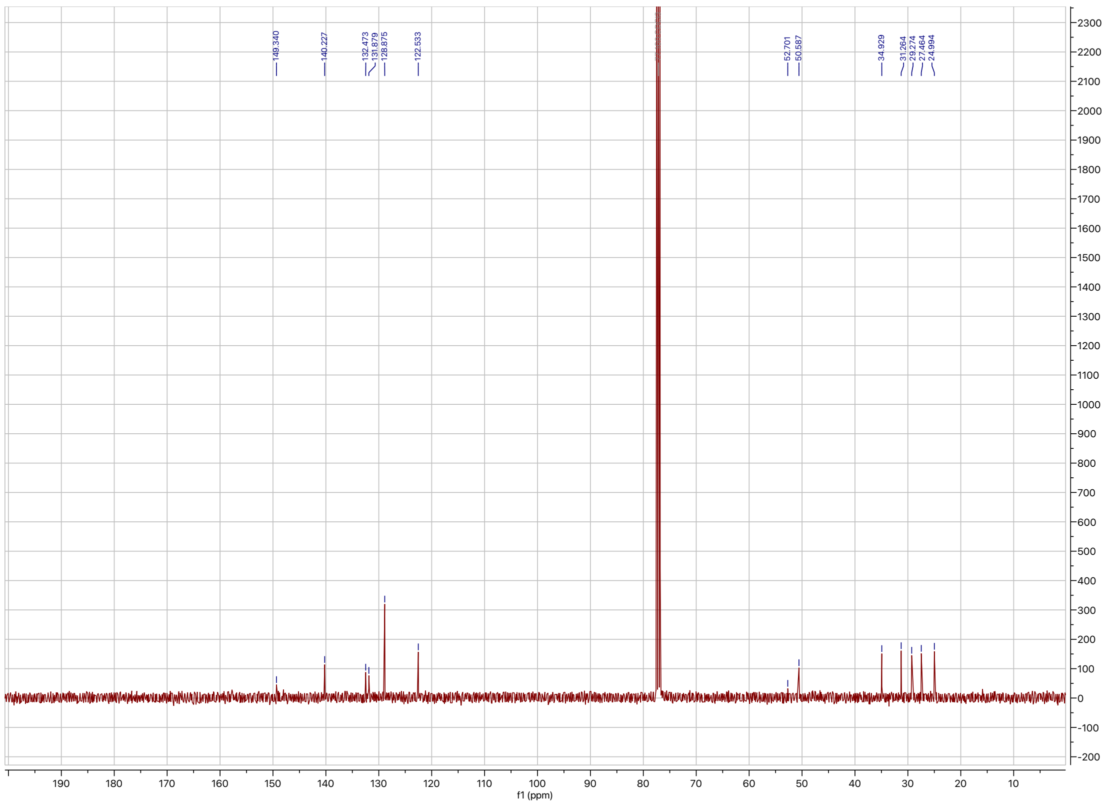

Figure S18. ^{13}C NMR spectrum of compound **3.** ($CDCl_3$, 298K)

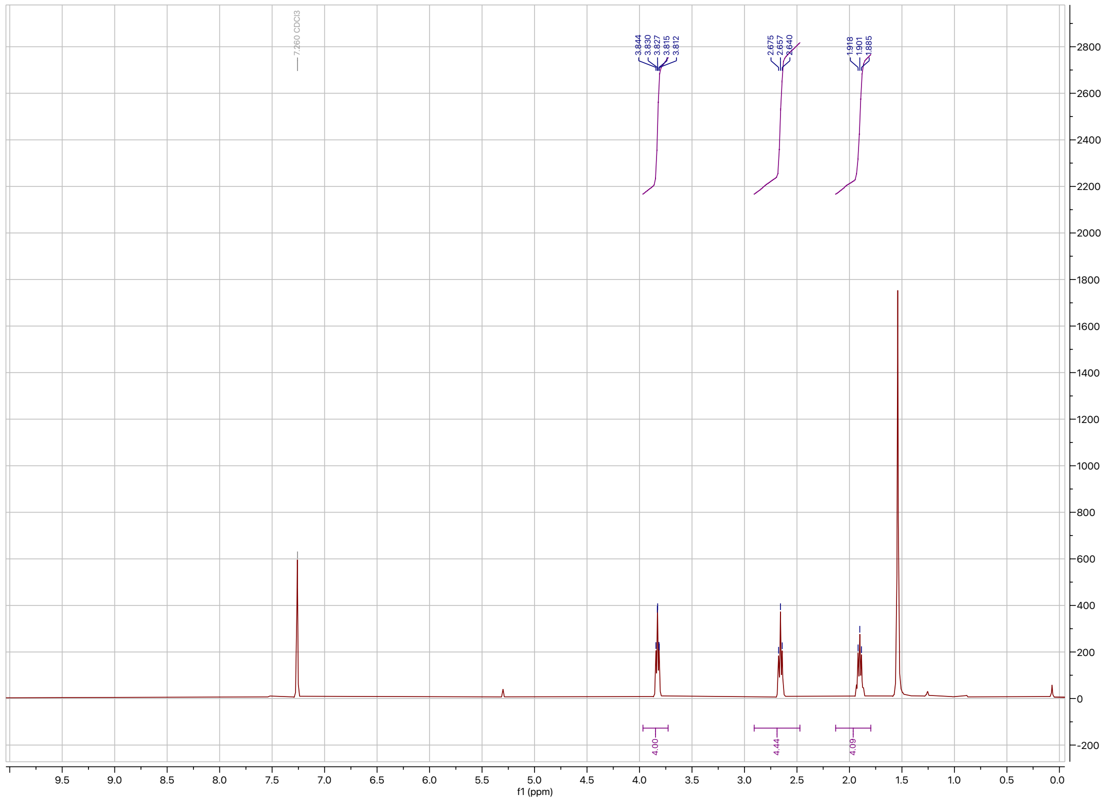

Figure S19. ^{1}H NMR spectrum of compound **17.** ($CDCl_3$, 298K)

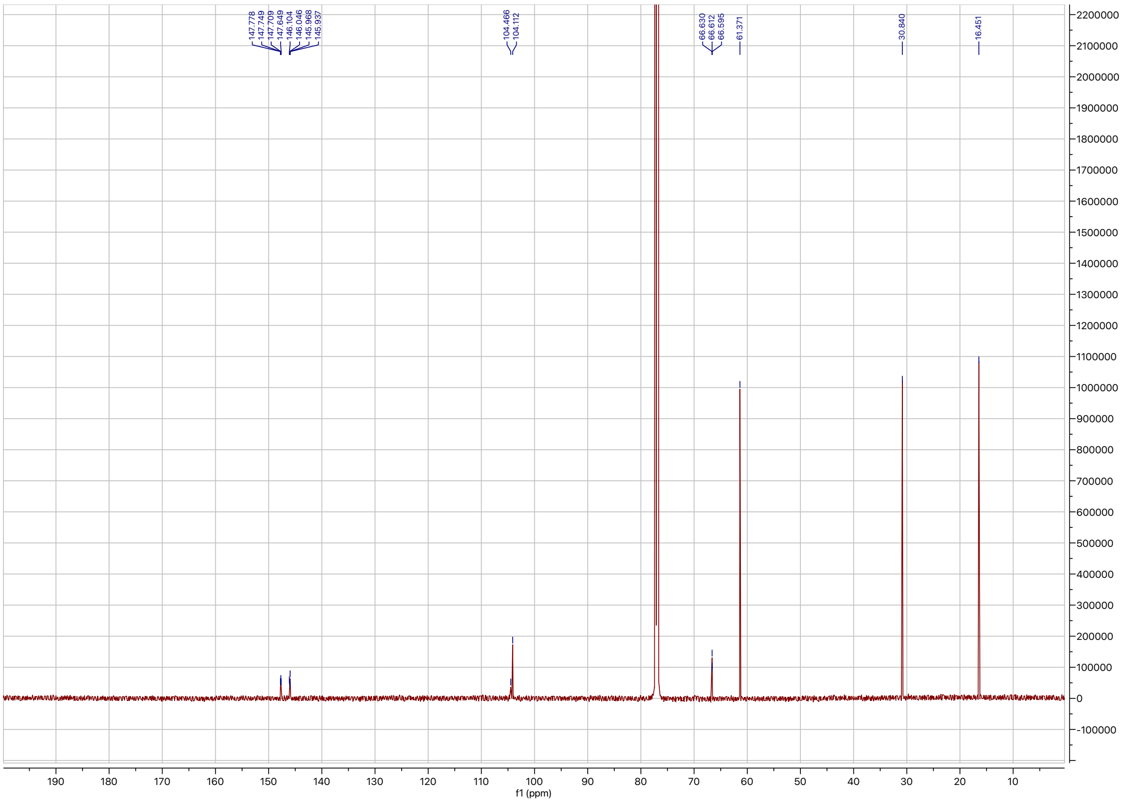

Figure S20. ¹³C NMR spectrum of compound **17.** ($CDCl_3$, 298K)

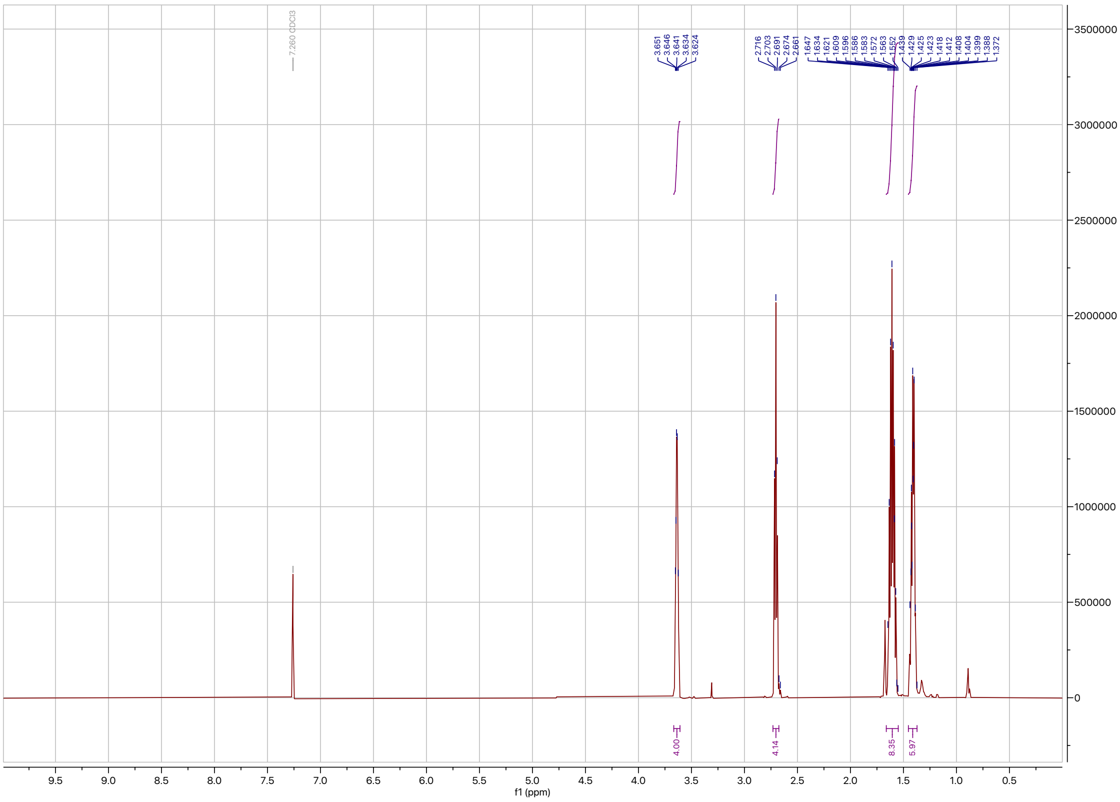

Figure S21. ¹H NMR spectrum of compound **18.** ($CDCl_3$, 298K)

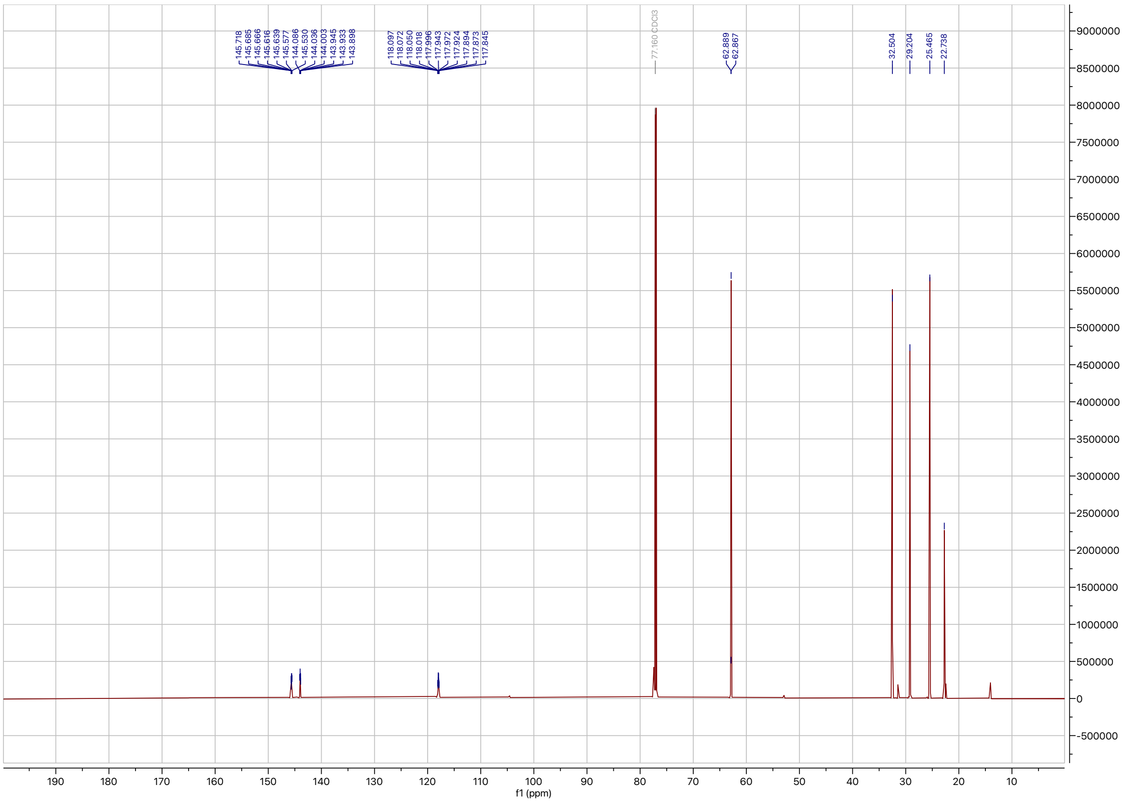

Figure S22. ^{13}C NMR spectrum of compound **18.** ($CDCl_3$, 298K)

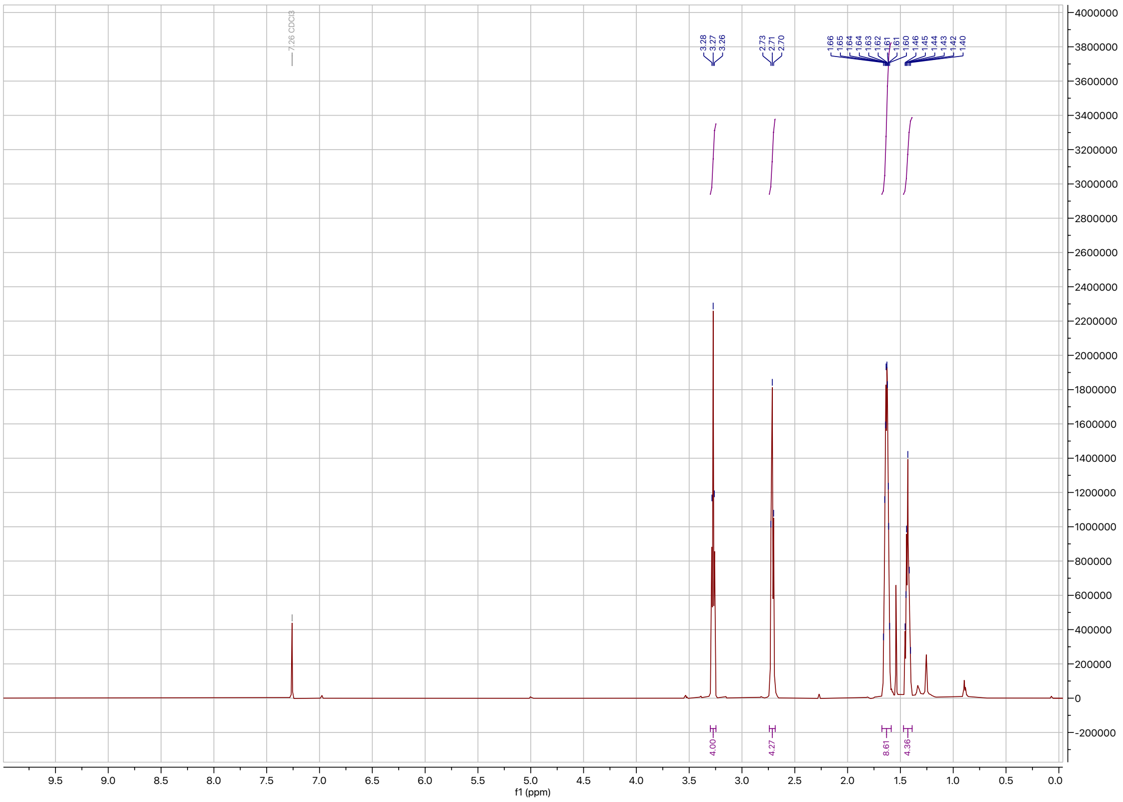

Figure S23. ^{1}H NMR spectrum of compound **19.** ($CDCl_3$, 298K)

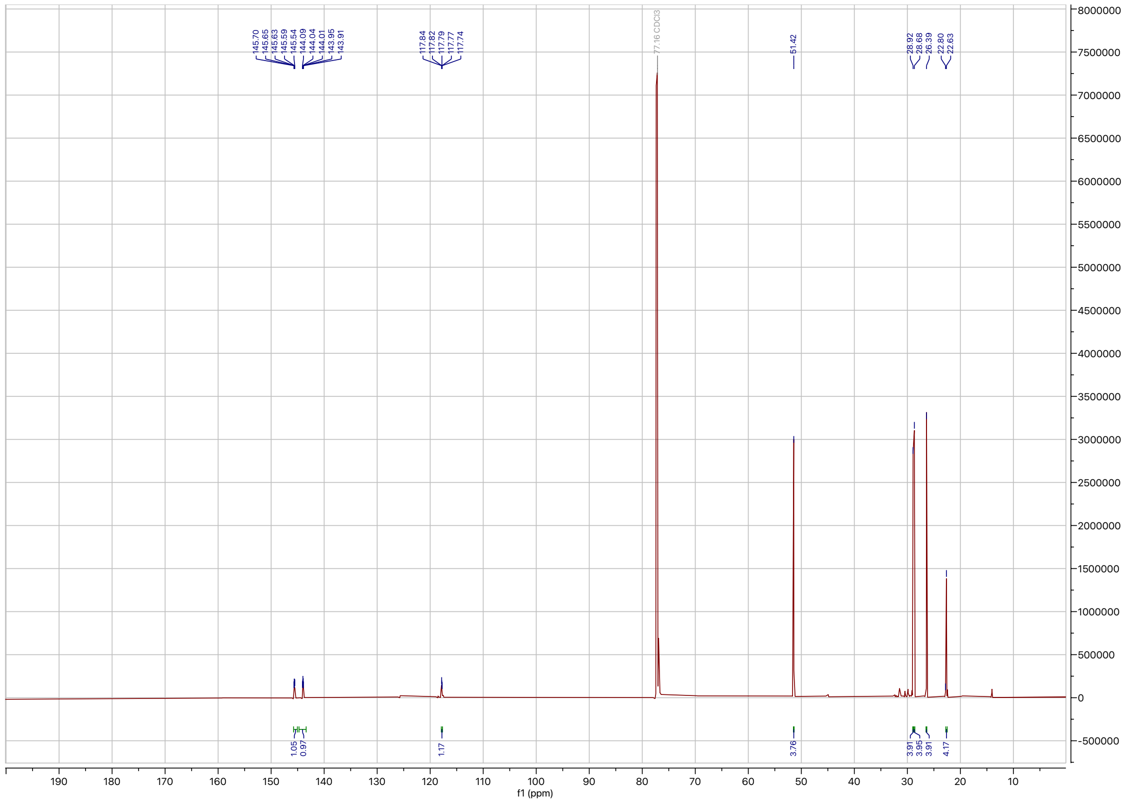

Z

Figure S24. ^{13}C NMR spectrum of compound **19.** ($CDCl_3$, 298K)

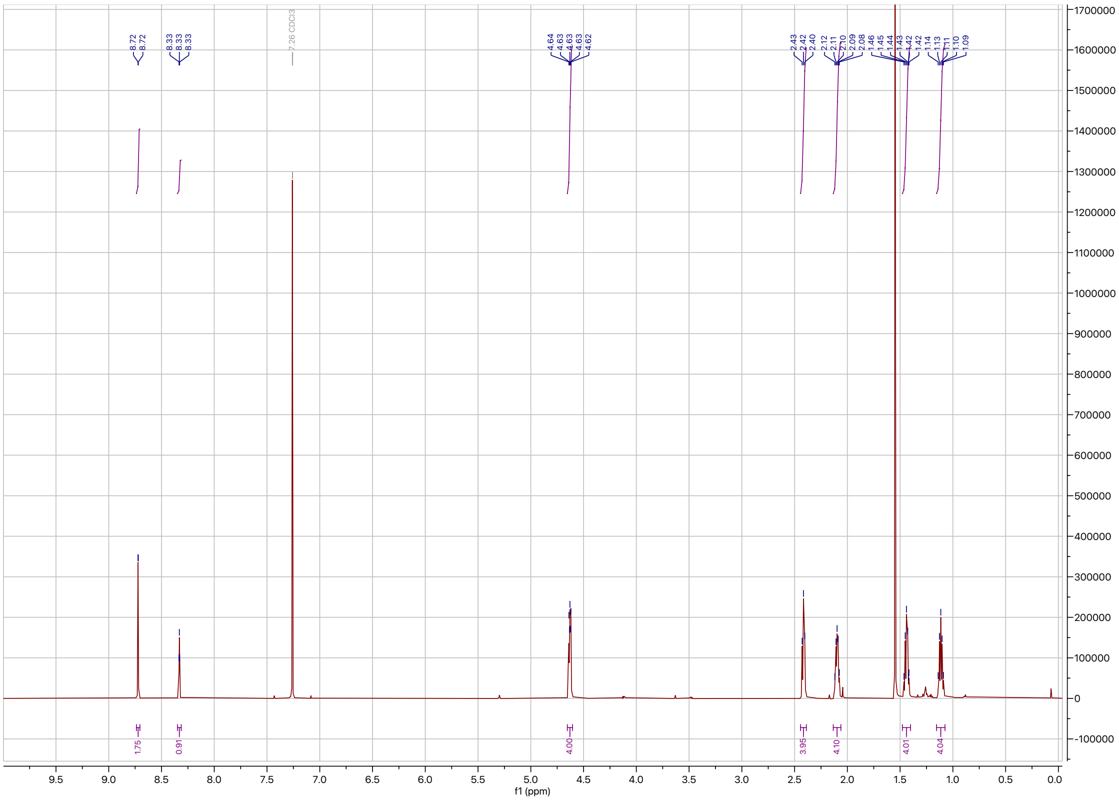

Figure S25. ^{1}H NMR spectrum of compound **4.** ($CDCl_3$, 298K)

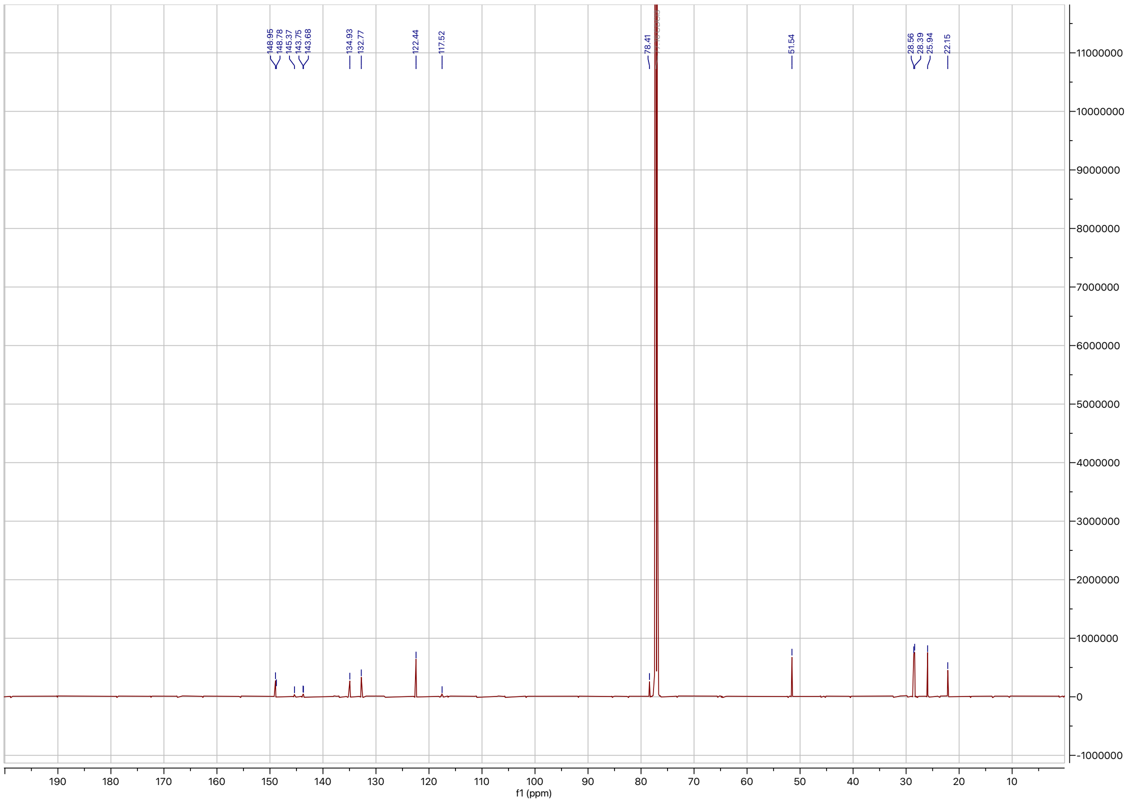

Figure S26. ^{13}C NMR spectrum of compound **4.** ($CDCl_3$, 298K)

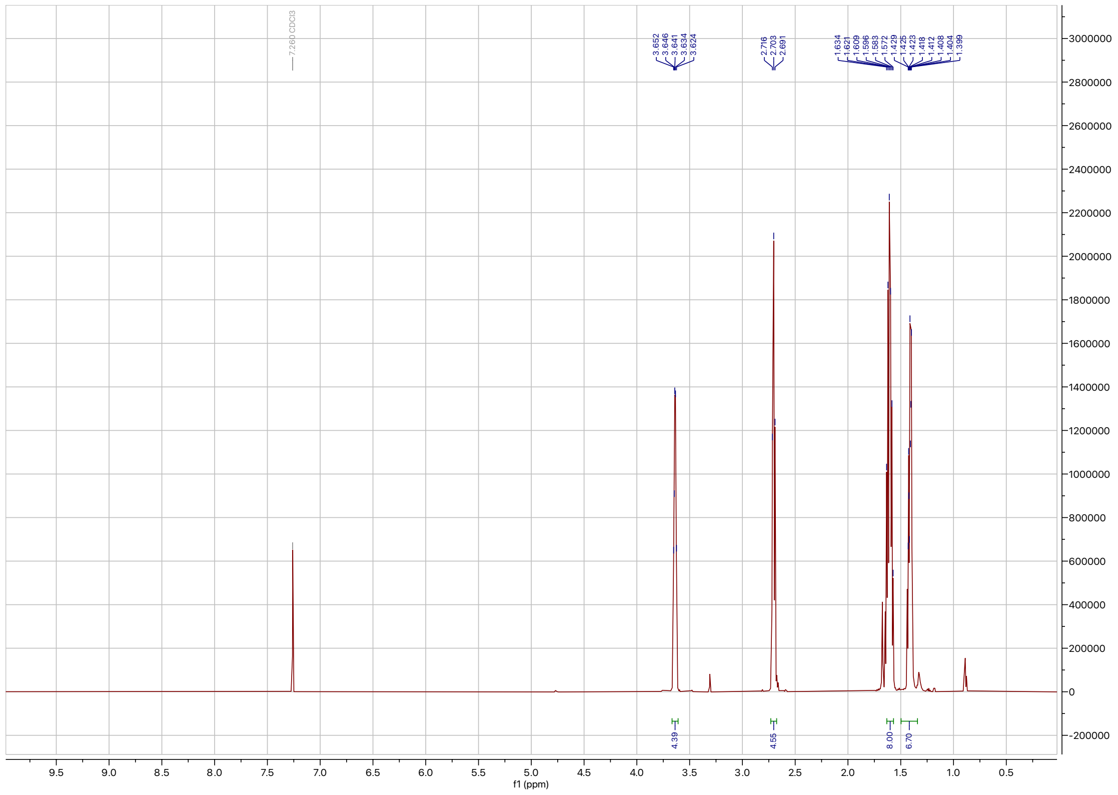

Figure S27. ^{1}H NMR spectrum of compound **20.** ($CDCl_3$, 298K)

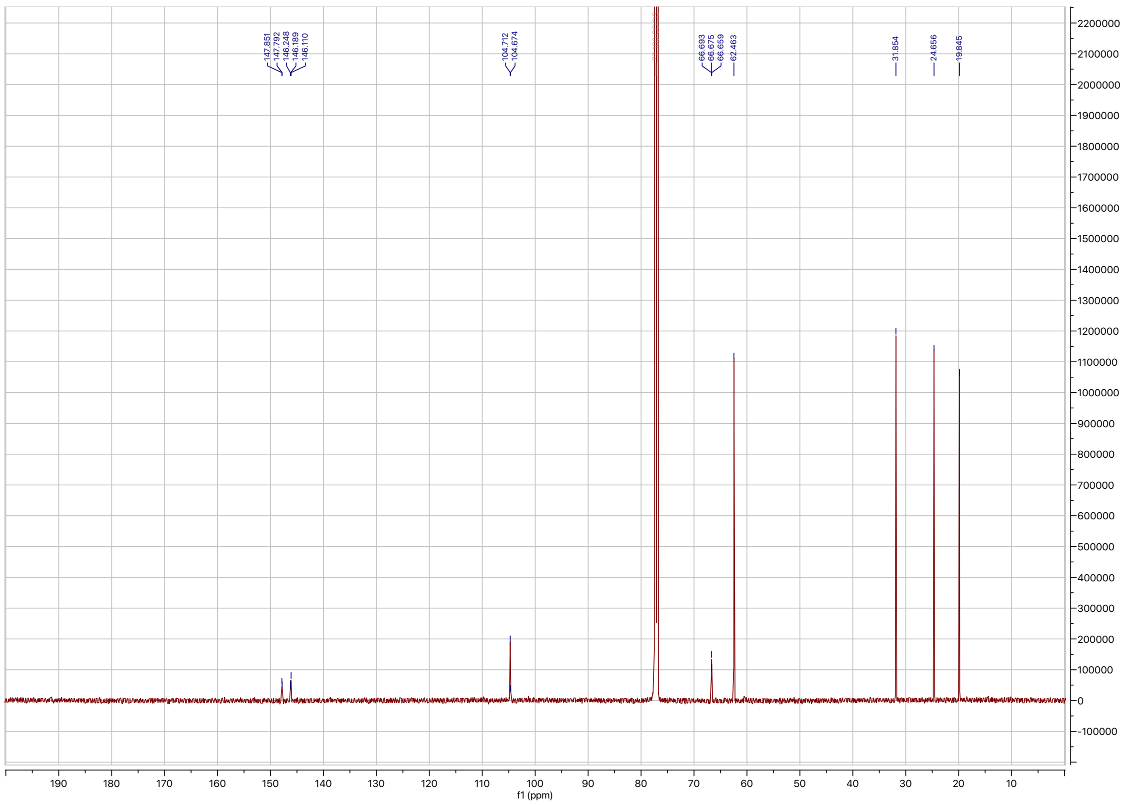

Figure S28. ^{13}C NMR spectrum of compound **20.** ($CDCl_3$, 298K)

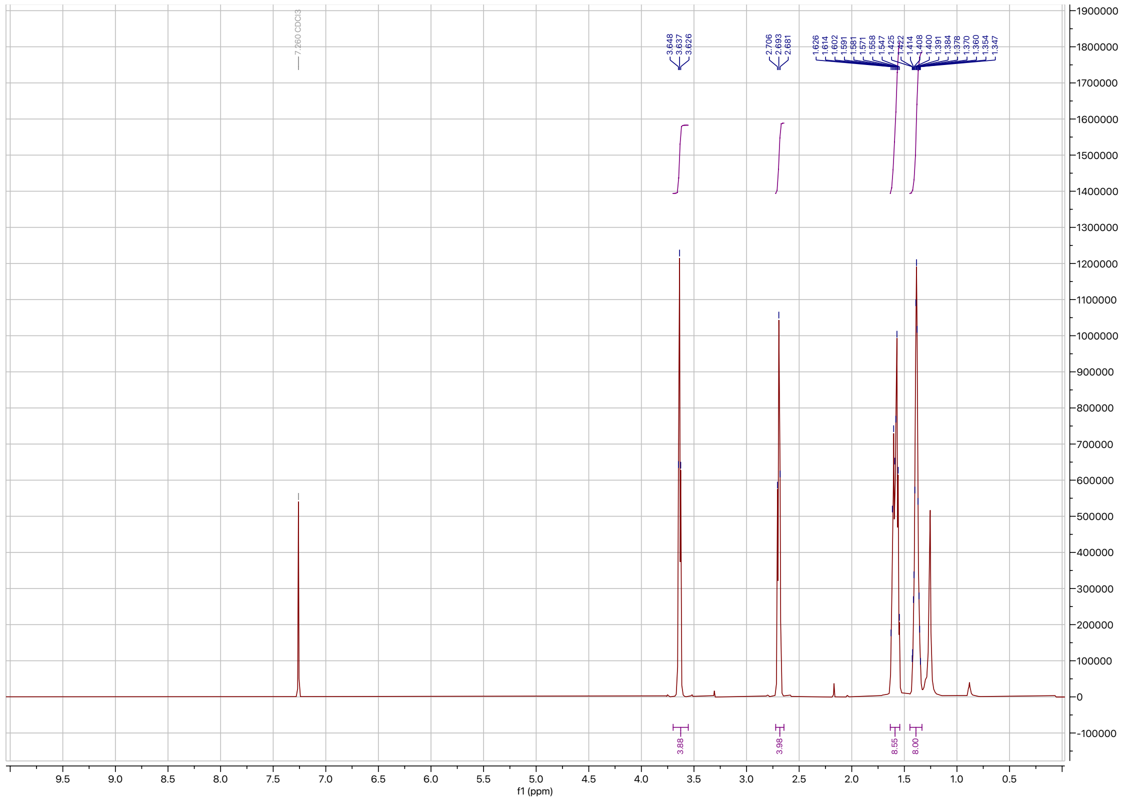

Figure S29. ^{1}H NMR spectrum of compound **21.** ($CDCl_3$, 298K)

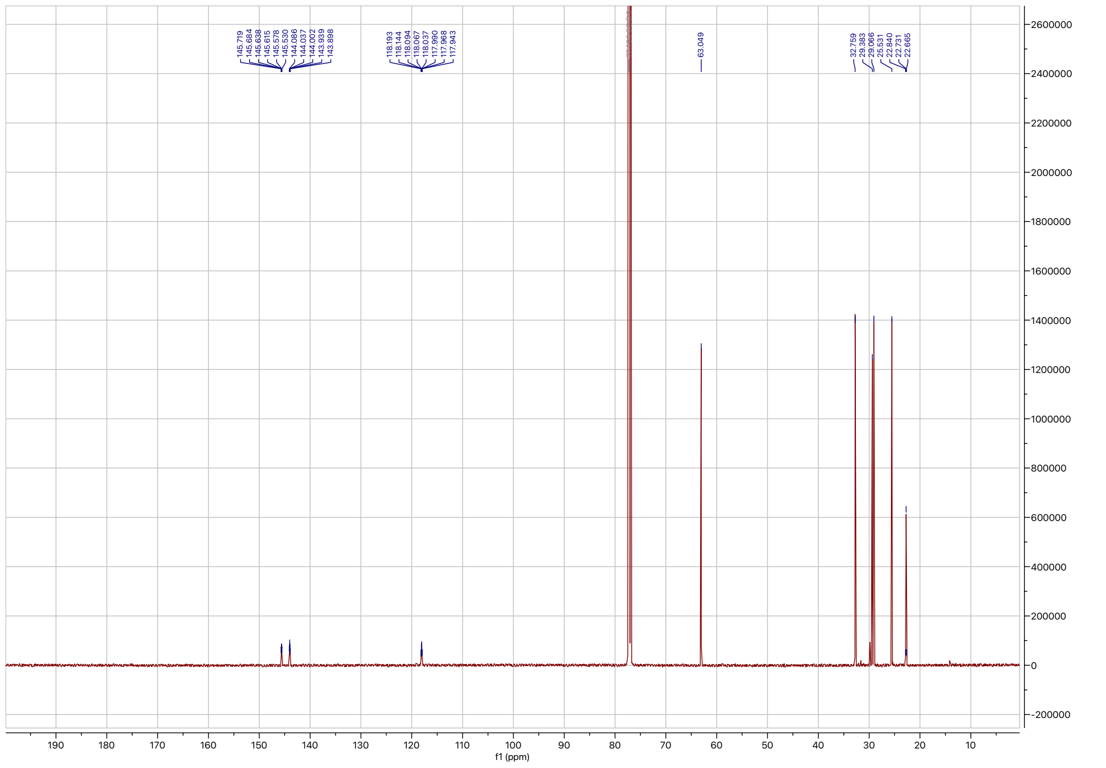

Figure S30. ^{13}C NMR spectrum of compound **21.** ($CDCl_3$, 298K)

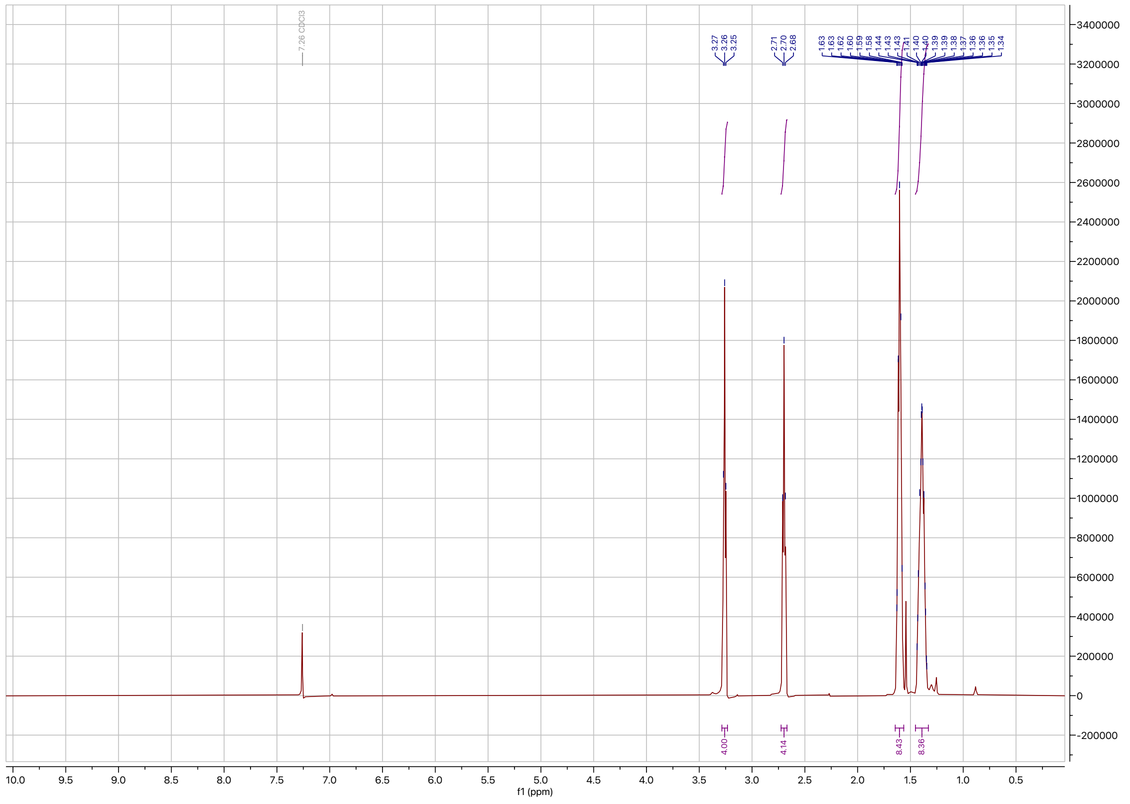

Figure S31. ^{1}H NMR spectrum of compound **22.** ($CDCl_3$, 298K)

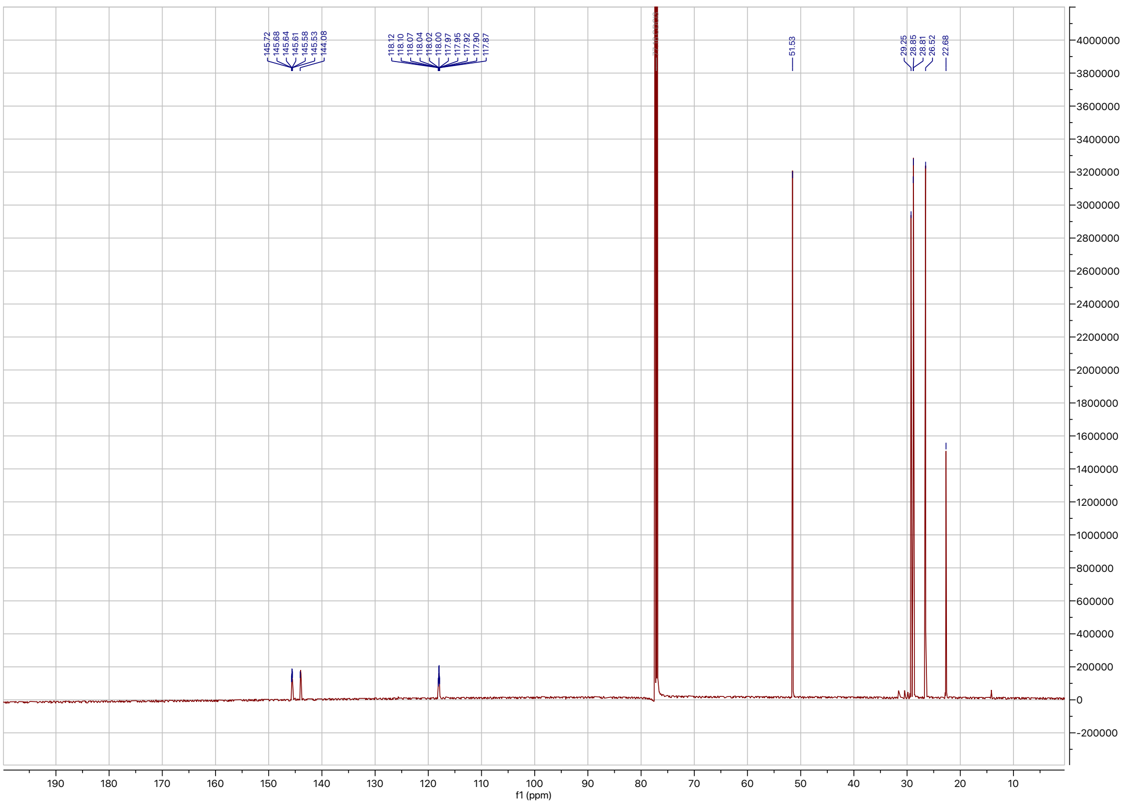

Figure S32. ^{13}C NMR spectrum of compound **22.** ($CDCl_3$, 298K)

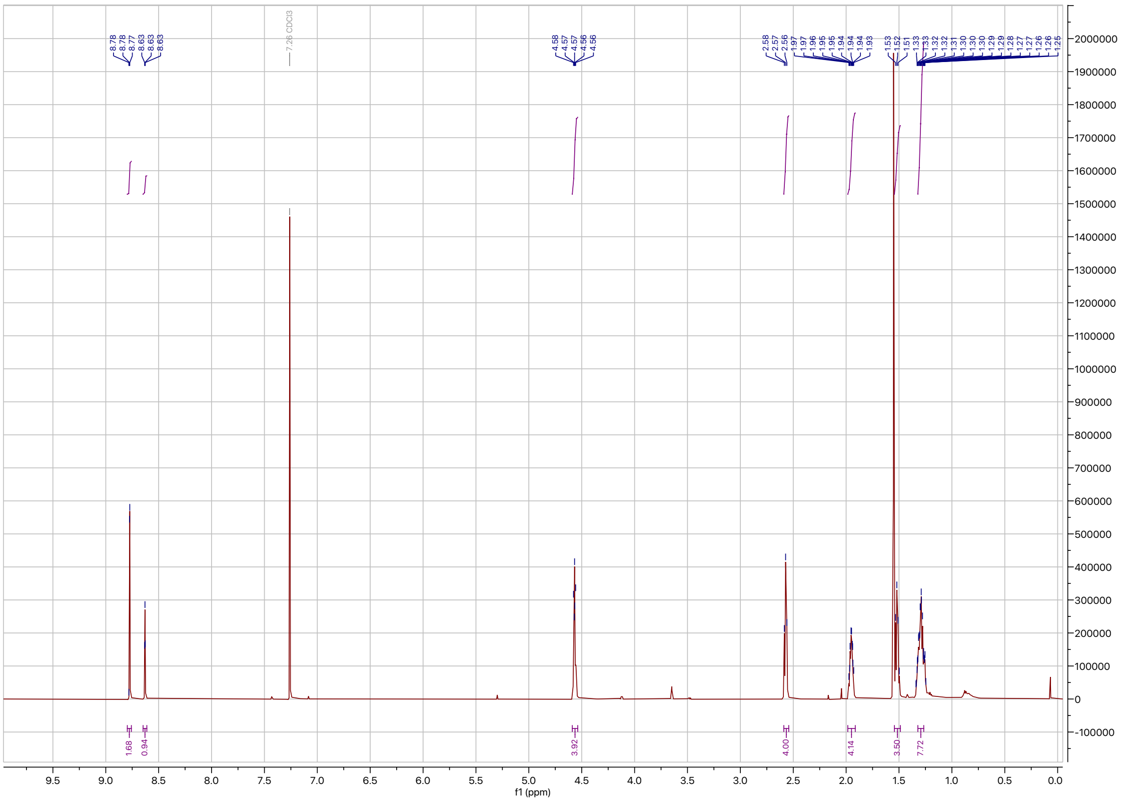

Figure S33. ^{1}H NMR spectrum of compound **5.** ($CDCl_3$, 298K)

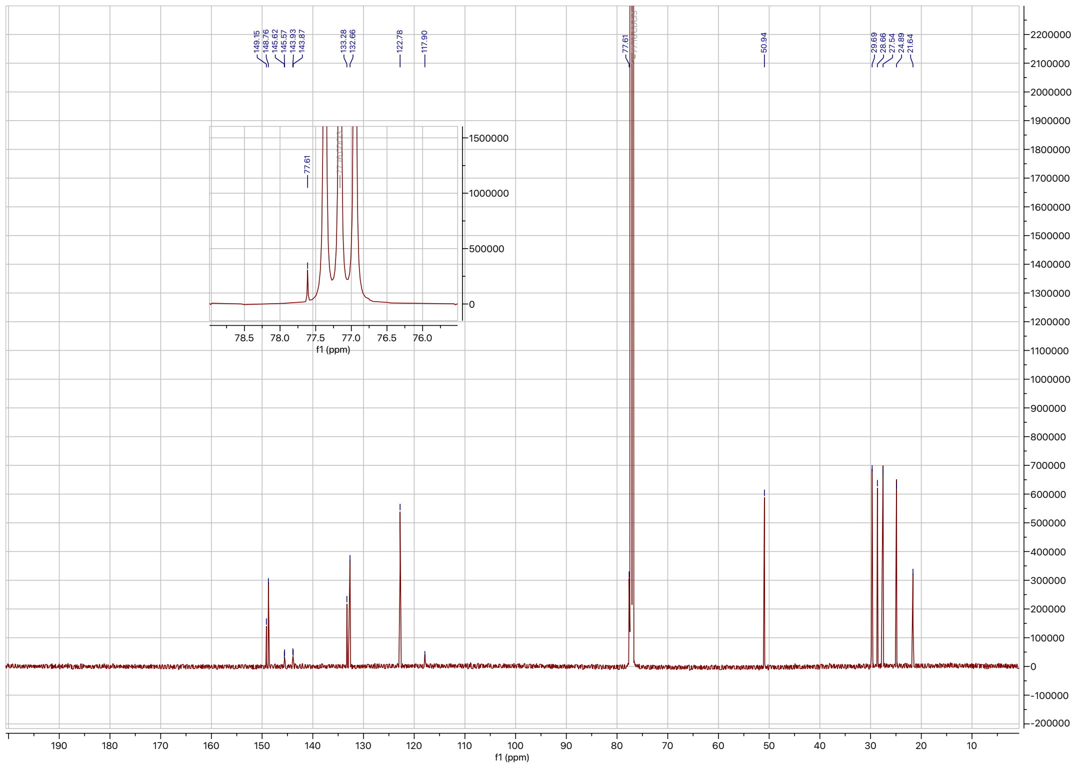

Figure S34. ^{13}C NMR spectrum of compound **5.** ($CDCl_3$, 298K)

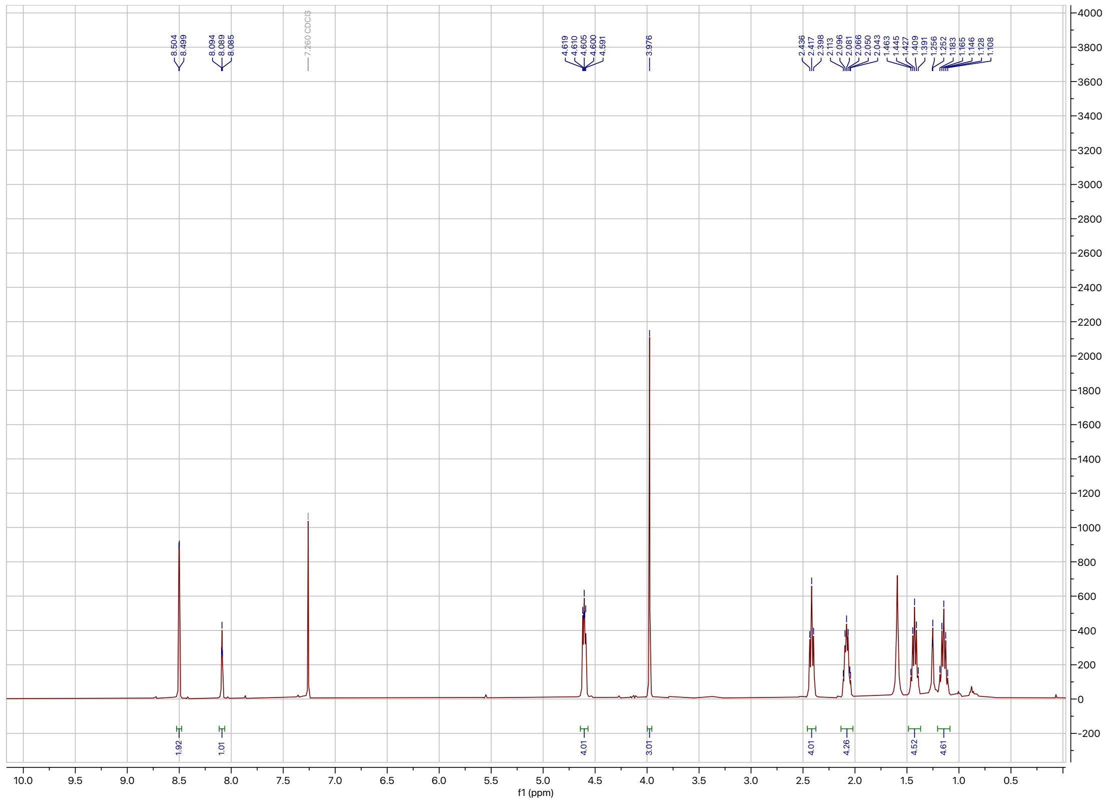

Figure S35. ^{1}H NMR spectrum of compound **6.** ($CDCl_3$, 298K)

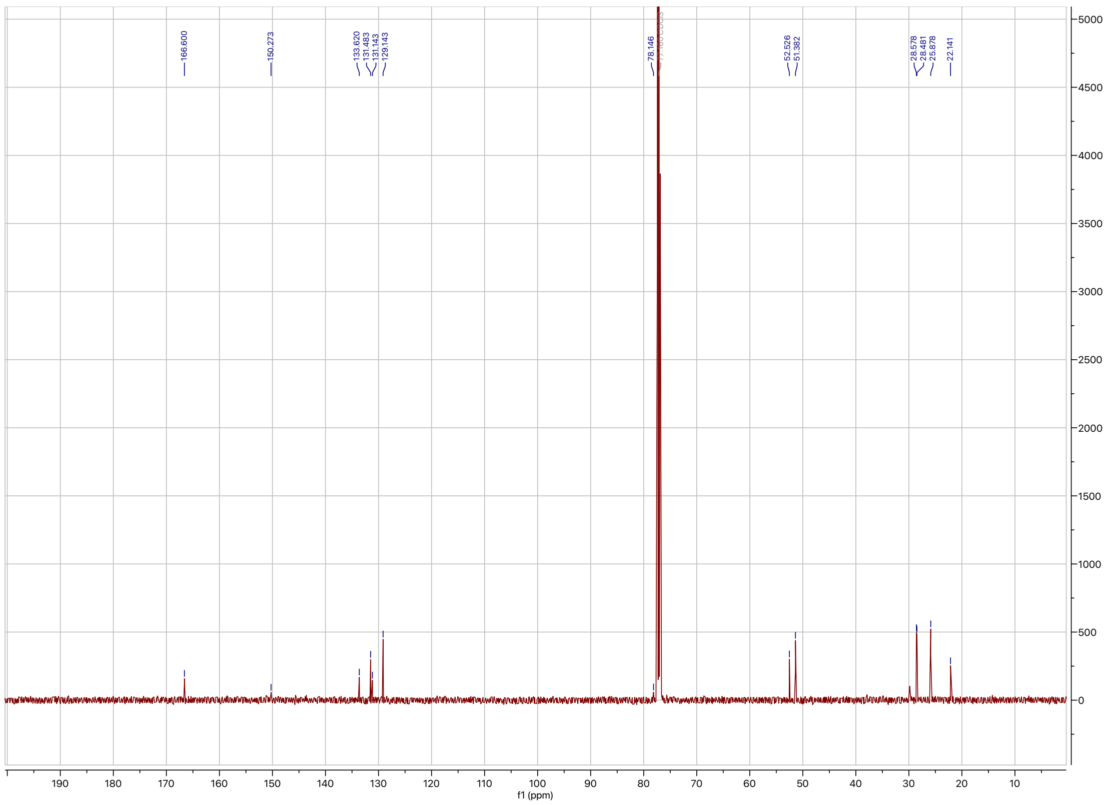

Figure S36. ^{13}C NMR spectrum of compound **6.** ($CDCl_3$, 298K)

3. Anion Transport Experiments

Vesicle Preparation

A thin film of lipid (1-palmitoyl-2-oleoyl-*sn*-3-phosphatidylcholine POPC, egg-yolk phosphatidylglycerol EYPG or dipalmitoyl phosphatidylcholine DPPC) was formed by evaporating a chloroform solution under reduced pressure on a rotary evaporator (40 °C) and then under high vacuum for 6 hours. The lipid film was hydrated by vortexing with the prepared buffer (100 mM NaCl, 10 mM HEPES, 1 mM 8-Hydroxypyrene-1,3,6-trisulfonic acid trisodium salt (HPTS), pH 7.0). The lipid suspension was then subjected to 5 freeze-thaw cycles using liquid nitrogen and a water bath (40°C) followed by extrusion 19 times through a polycarbonate membrane (pore size 200 nm). Extra-vesiclular components were removed by size exclusion chromatography on a Sephadex G-25 column with 100 mM NaCl, 10 mM HEPES, pH 7.0. Final conditions: LUVs (2.5 mM lipid); inside 100 mM NaCl, 10 mM HEPES, 1 mM HPTS, pH 7.0; outside: 100 mM NaCl, 10 mM HEPES, pH 7.0. Vesicles for the sodium gluconate assay were prepared by the same procedure, substituting NaCl for NaGluconate in the buffer solution.

Transport Assays with HPTS[9,10]

In a typical experiment, the LUVs containing HPTS (37.5 µL, final lipid concentration 31.3 µM) were added to buffer (2925 µL of 100 mM NaCl, 10 mM HEPES, pH 7.0) at 25°C under gentle stirring. A pulse of NaOH (30 µL, 0.5 M) was added at 40 secs to initiate the experiment. At 90 s the test transporter (various concentrations, in 7.5 µL DMSO) was added, followed by detergent (37.5 µL of Triton X-100 in 7:1 (v/v) H_2O-DMSO) at 300 secs to calibrate the assay. The fluorescence emission was monitored at λ_{em} = 510 nm (λ_{ex} = 460/405 nm). The fractional fluorescence intensity (I_{rel}) was calculated from equation (S1), where R_t is the fluorescence ratio at time t, R_0 is the fluorescence ratio at time 0, and R_d is the fluorescence ratio after the addition of detergent.

$$I_{rel} = \frac{R_t - R_0}{R_d - R_0} \qquad \text{(S1)}$$

The fractional fluorescence intensity (I_{rel}) at 288 s just prior to lysis, defined as the fractional activity y, was plotted as a function of the ionophore concentration (M). Hill coefficients (n) and EC_{50} values were calculated by fitting to the Hill equation (S2):

$$y = y_0 + (y_{max} - y_0)\,\frac{x^n}{{EC_{50}}^n - x^n} \qquad \text{(S2)}$$

where y_0 is the baseline fractional activity in the absence of transporter, y_{max} is the fractional activity in with excess transporter, x is the transporter concentration in the cuvette. Where full Hill plots were fitted, at least 7 data points spanning the required concentration range were considered, and each individual concentration was repeated at least twice and averaged.

Experiments with DPPC lipids were conducted in the same way. For elevated temperature studies, the buffer was equilibrated at 45°C (using the Peltier temperature controller) for 5 minutes prior to initiating the experiment.

Experiments in the presence of protonophore trifluoromethoxy carbonylcyanide phenylhydrazone (FCCP) were carried out using the above procedure, except that a DMSO solution of FCCP (7.5 µL of 100µM solution, final concentration 0.25 µM/0.8 mol%) was added to the vesicle suspension following the addition of the NaOH pulse (at 60s).

HPTS Assay Data for all Transporters

In the following figures: Left: change in relative fluorescence intensity over time in the HPTS assay (LUVs (31.25 µM lipid); inside 100 mM NaCl, 10 mM HEPES, 1 mM HPTS, pH 7.0; outside: 100 mM NaCl, 10 mM HEPES, pH 7.0). Shaded area indicates two standard deviations. Right: dependence of the fractional transport activity *y* in the HPTS assay on the concentration of transporter (blue circles squares), and fitted to the Hill equation (blue line).

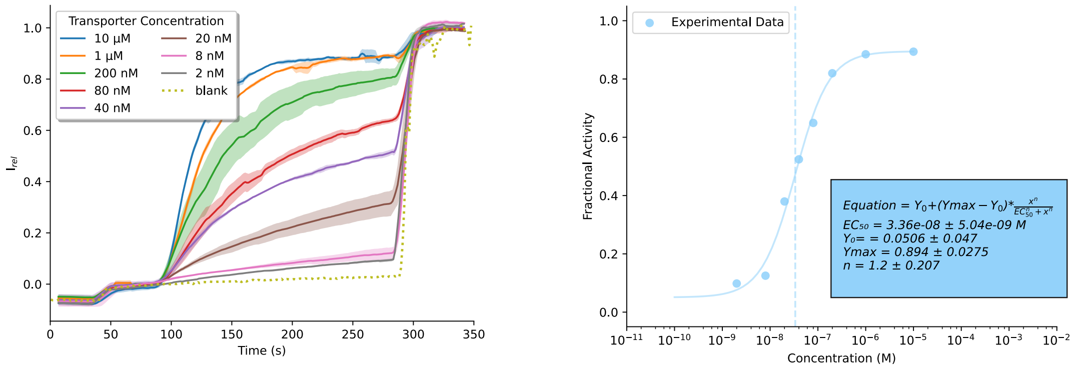

Figure S37. HPTS assay data for carrier **2** with FCCP.

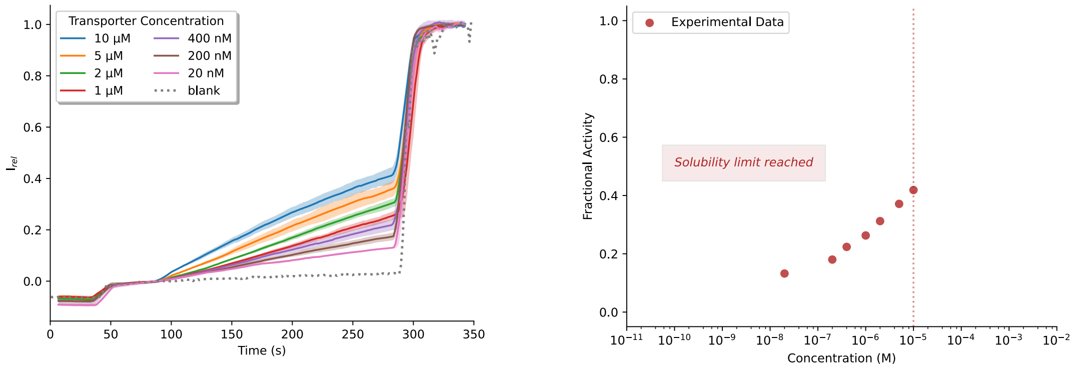

Figure S38. HPTS assay data for carrier **2** without FCCP.

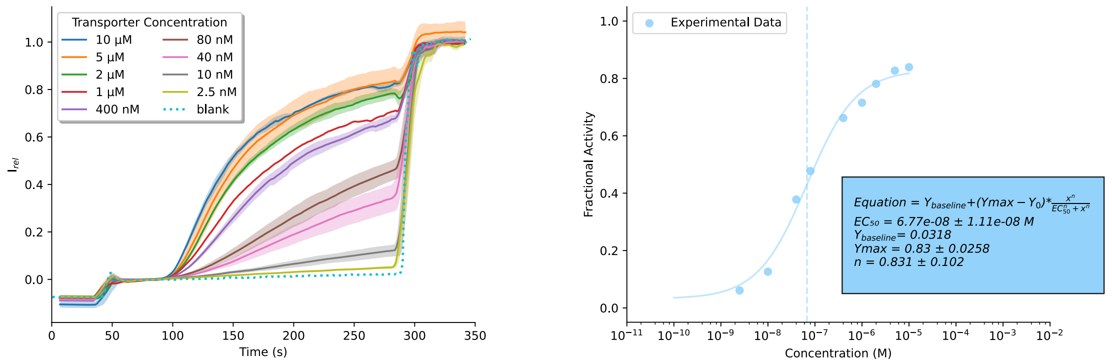

Figure S39. HPTS assay data for carrier **3** with FCCP.

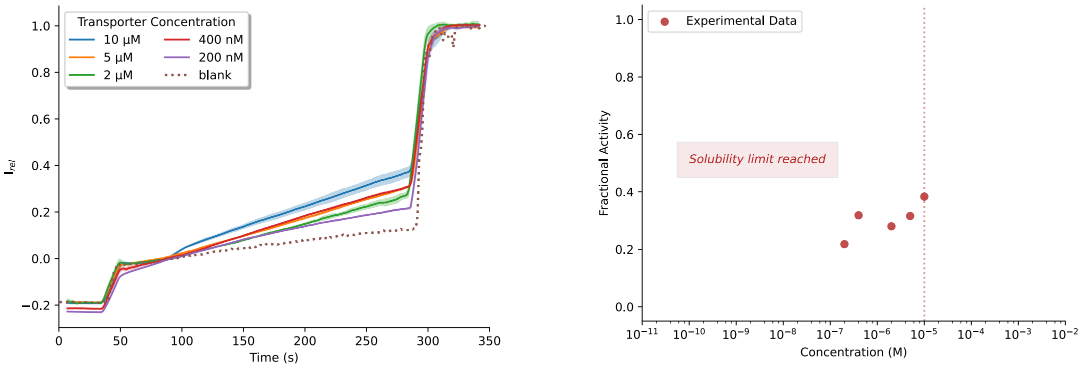

Figure S40. HPTS assay data for carrier **3** without FCCP.

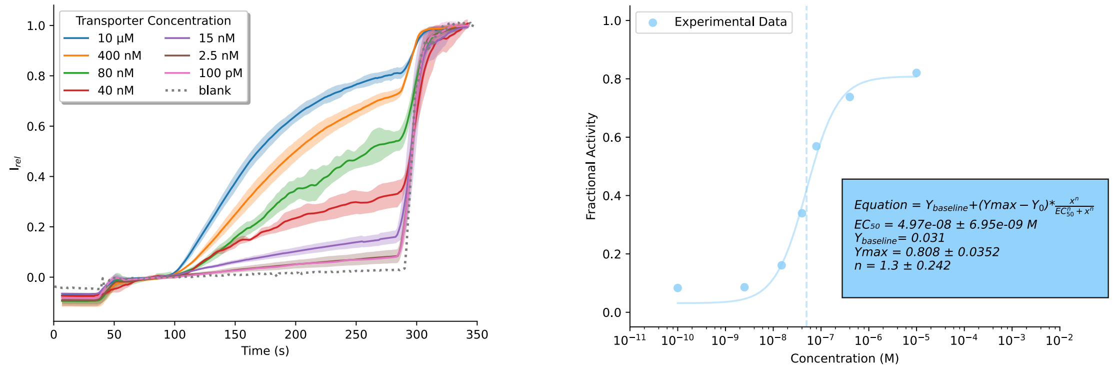

Figure S41. HPTS assay data for carrier **4** with FCCP.

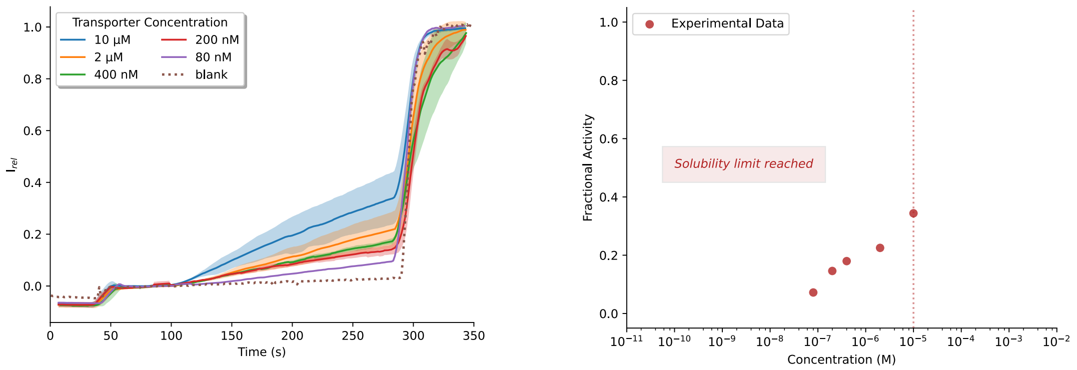

Figure S42. HPTS assay data for carrier **4** without FCCP.

Data for Transporter 2 with BSA

In experiments with BSA, fatty acid-free BSA was dissolved in the vesicle stock suspension to a final BSA concentration of 1 mol% (with respect to lipid). The BSA-containing vesicle stock suspension was stirred for 20 min before being used for membrane transport studies according the procedure described above.

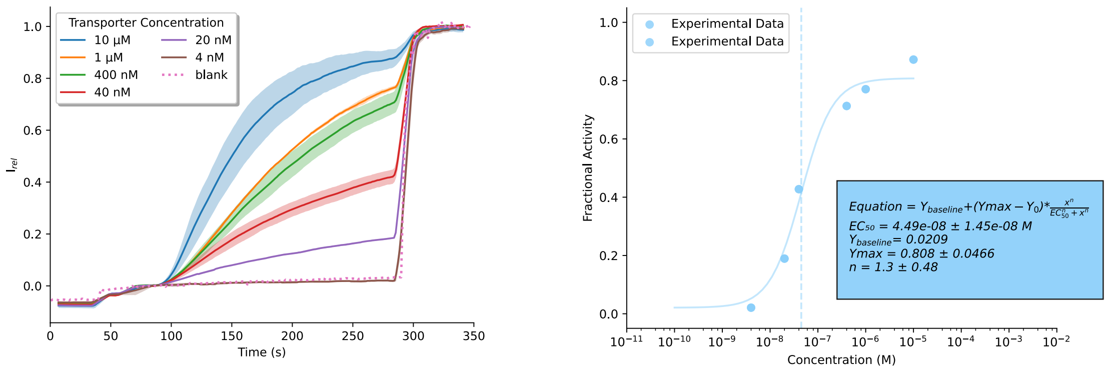

Figure S43. HPTS assay data for carrier **2** with BSA and with FCCP.

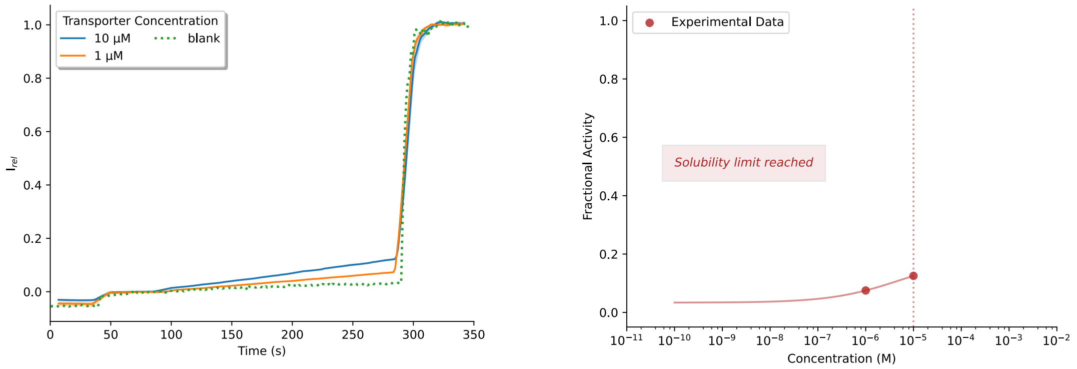

Figure S44. HPTS assay data for carrier **2** with BSA and without FCCP.

Sodium Gluconate Assay Data for all Transporters

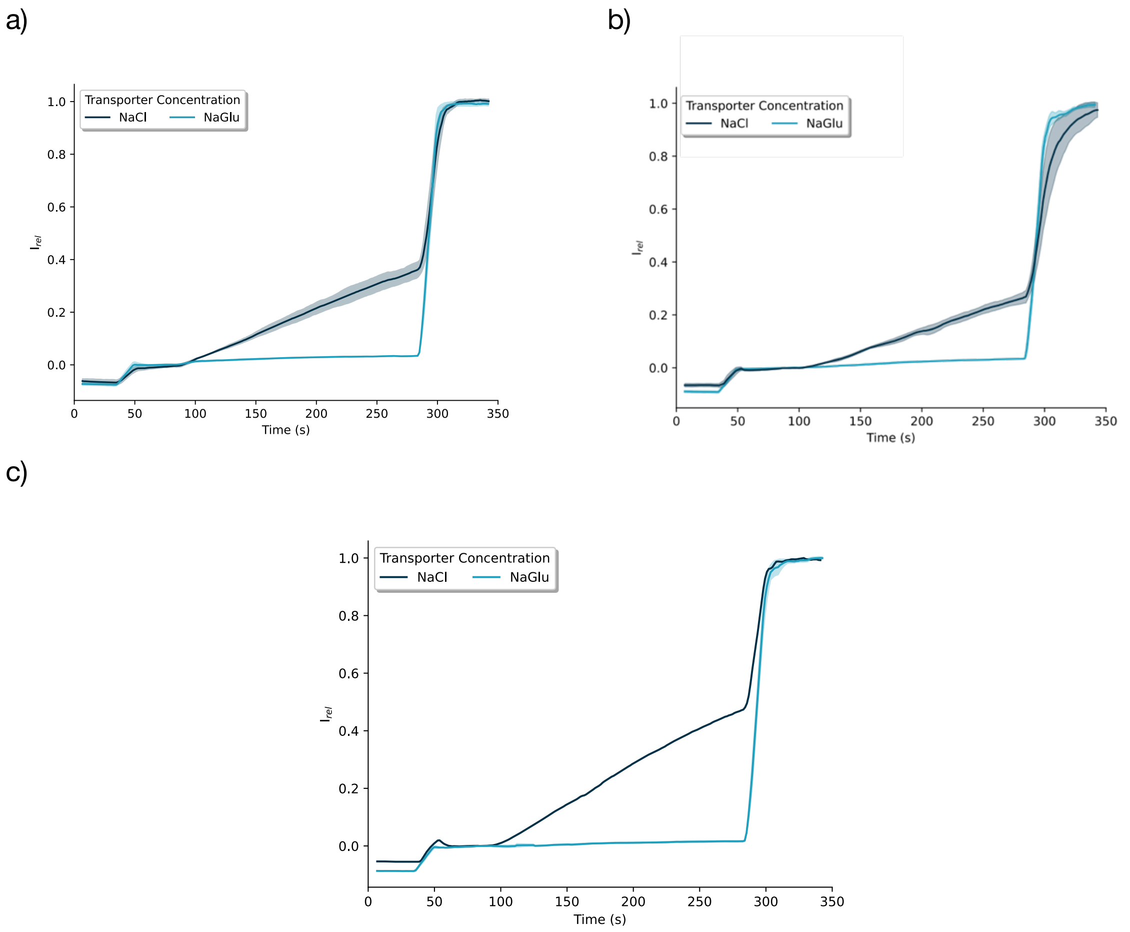

Figure S45. The time-dependent change in fluorescence intensity within the gluconate variation of the HPTS assay. Carriers were administered at 5 uM. (a) **2**, (b) **3** (c) **4**.

The decrease in transport with NaGlu for all carriers indicated they do not facilitate sodium cation transport (via H^+/Na^+ antiport), and must therefore operate via an anion transport mechanism.

Membrane Fluidity Studies

a)

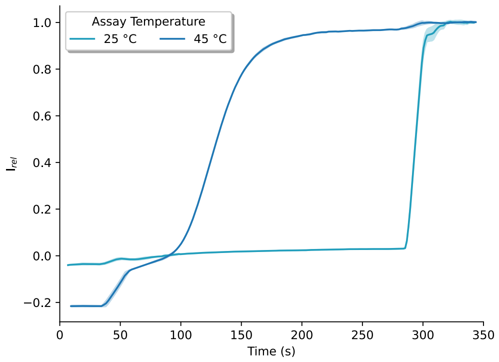

b)

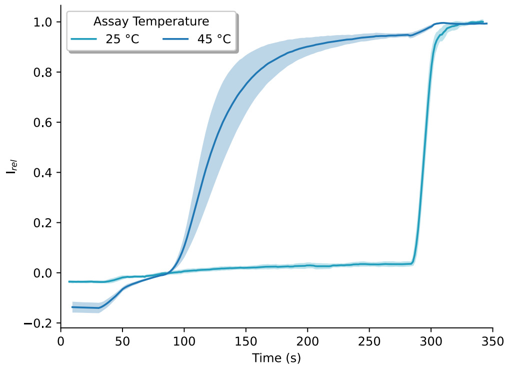

c)

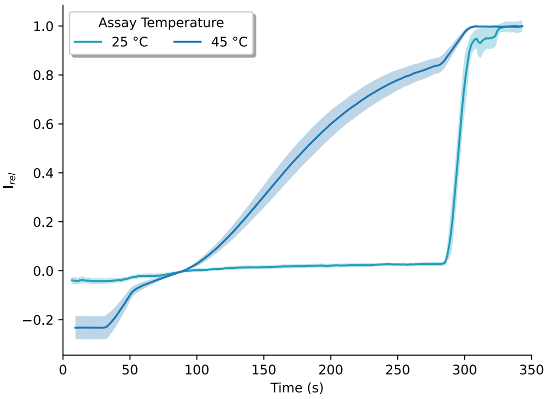

Figure S46. Change in relative fluorescence over time in the HPTS assay utilizing DPPC LUVs at 25°C and 45°C, with the temperature controlled by a Peltier temperature controller. Carriers were administered at 1 µM at 90s following the addition of FCCP at 40s. (a) **2**, (b) **3** (c) **4**.
Lack of transport in the gel phase at 25°C, and restoration above the phase transition temperature (41°C) at 45°C, is consistent with a mobile carrier transport mechanism.

4. NMR Titration Experiments

All binding constants were measured by ^{1}H NMR titrations in a Bruker AVIII 500 spectrometer at 500 MHz and 298 K. The host (anionophores **2**, **3**, **4**, **5** and **6**) was dissolved in acetone-*d*6, or 2.5% D_2O-acetone-*d*6 (v/v) mixtures, at 1 mM concentration and a known volume (0.5 mL) added to the NMR tube. Known volumes of anion guest (added as the TBA salt, 50 mM) in 2.5% D_2O-acetone-*d*6 (v/v) mixtures were added and the spectra were recorded after each addition. The chemical shift perturbations of the host spectra were monitored as a function of guest concentration. In all cases the downfield perturbation of the internal aryl signal of the bis-iodotriazole motif was monitored (Figures S47-51). The data was analysed using a global fit procedure using the Bindfit[11] program, using non-linear least squares analysis to obtain the best fit between observed and calculated chemical shifts for the 1:1 binding stoichiometry. In all experiments the association of guest and host was fast on the NMR timescale.

In the following figures: ^{1}H NMR titration spectra (top) and fitted 1:1 binding isotherms (bottom) fitted to concentration dependence of the downfield perturbation of the internal aryl signal of the bis-iodotriazole motif

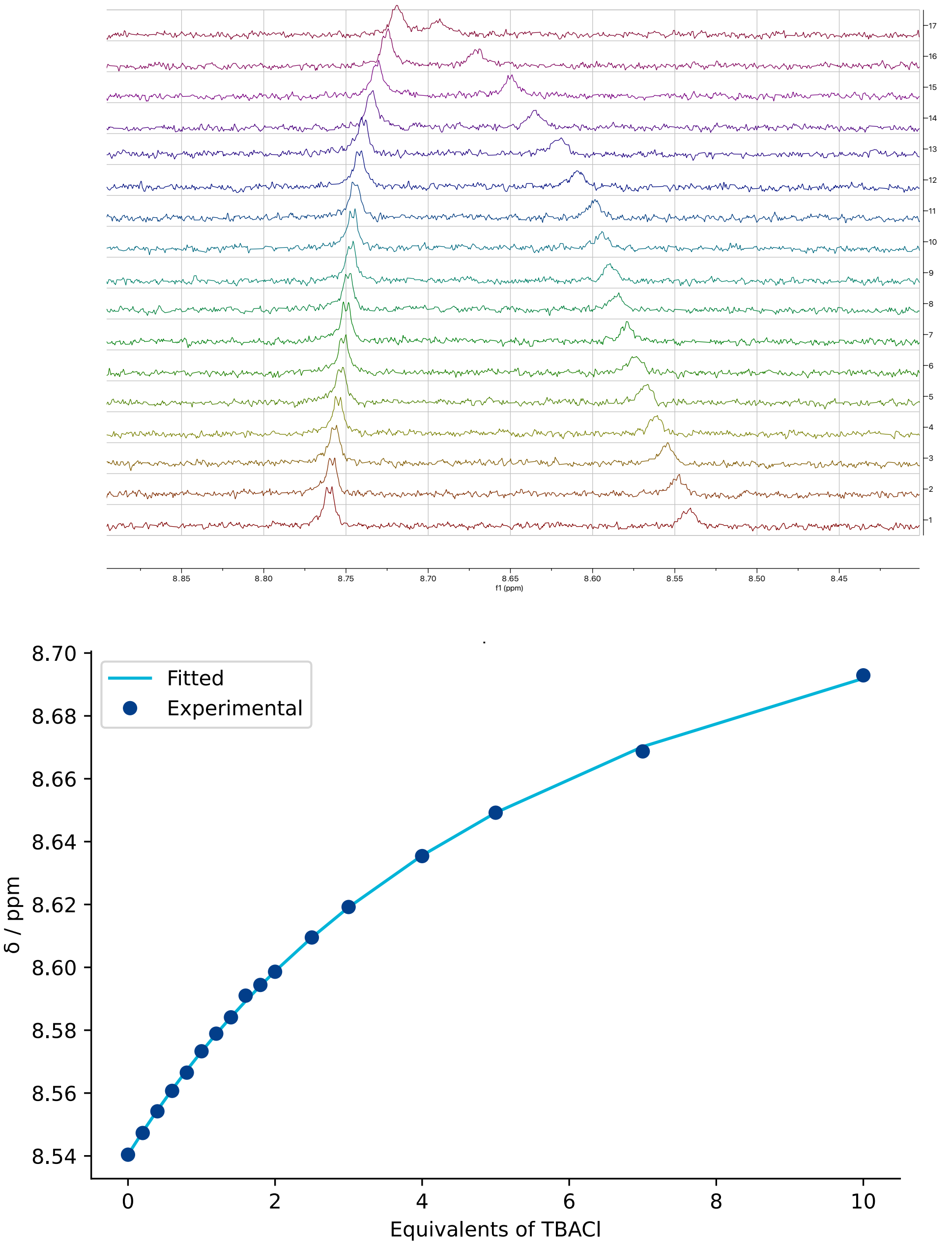

Figure S47. Top: Truncated ^{1}H NMR spectra of **2** upon titration with TBACl in 2.5% D_2O/acetone-d6 (v/v) from 0 to 10 equivalents. Bottom: Corresponding Cl^- binding isotherm. Experimental data shown by ●, fitted 1:1 binding isotherm shown by solid line.

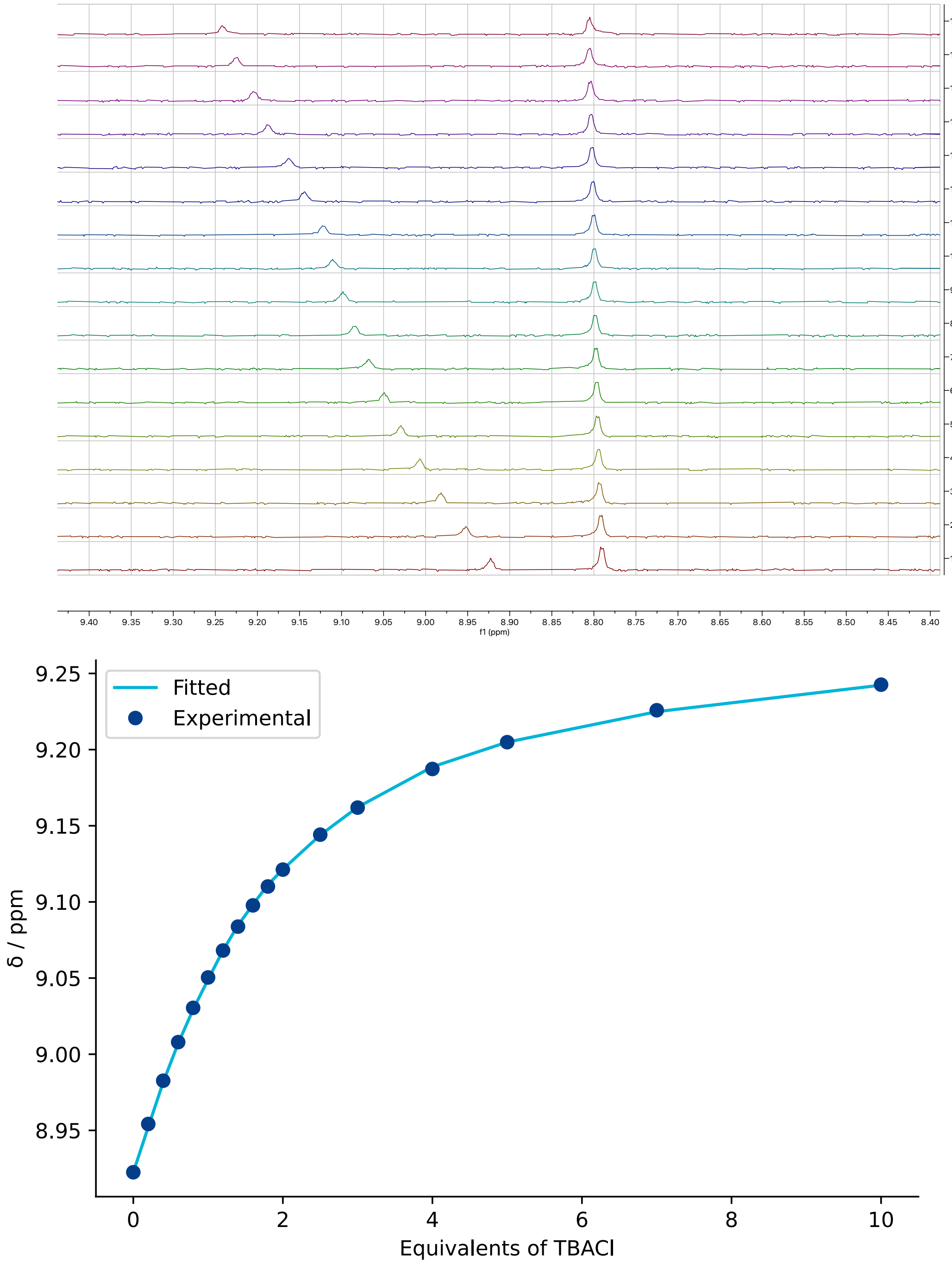

Figure S48. Top: Truncated ^{1}H NMR spectra of **3** upon titration with TBACl in 2.5% D_2O/acetone-d6 (v/v) from 0 to 10 equivalents. Bottom: Corresponding Cl^- binding isotherm. Experimental data shown by ●, fitted 1:1 binding isotherm shown by solid line.

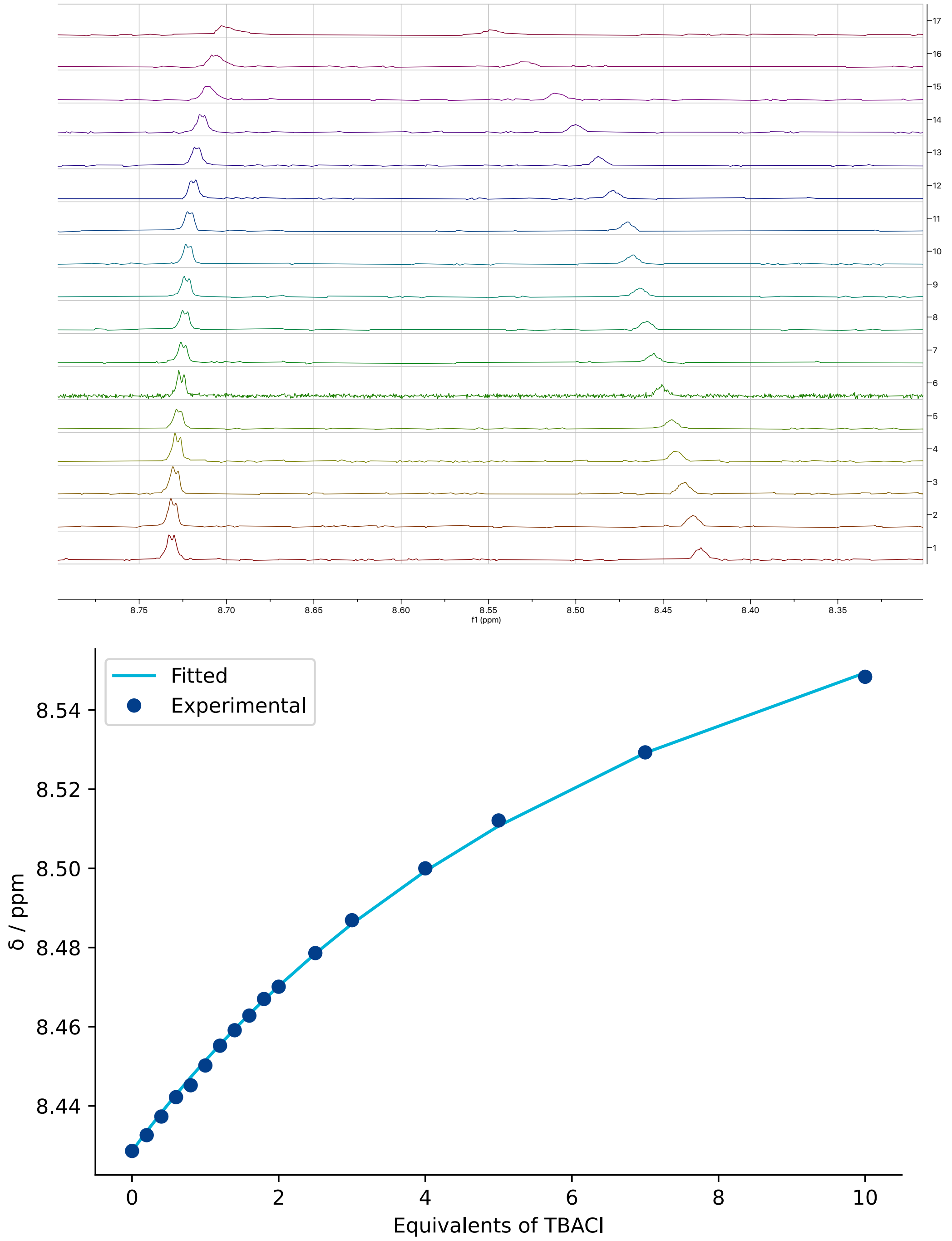

Figure S49. Top: Truncated ^{1}H NMR spectra of **4** upon titration with TBACl in 2.5% D_2O/acetone-d6 (v/v) from 0 to 10 equivalents. Bottom: Corresponding Cl^- binding isotherm. Experimental data shown by ●, fitted 1:1 binding isotherm shown by solid line.

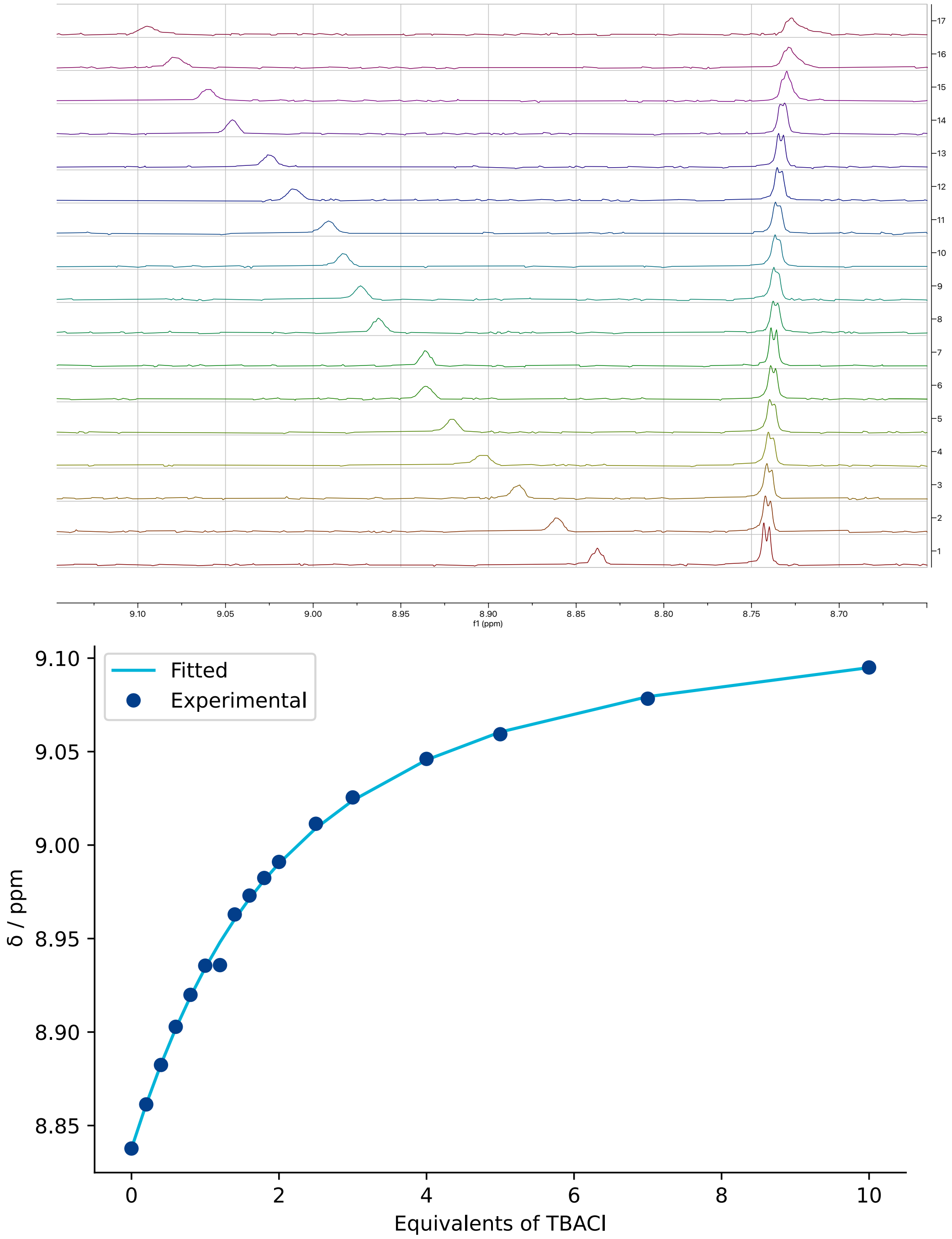

Figure S50. Top: Truncated ^{1}H NMR spectra of **5** upon titration with TBACl in 2.5% D_2O/acetone-d6 (v/v) from 0 to 10 equivalents. Bottom: Corresponding Cl^- binding isotherm. Experimental data shown by ●, fitted 1:1 binding isotherm shown by solid line.

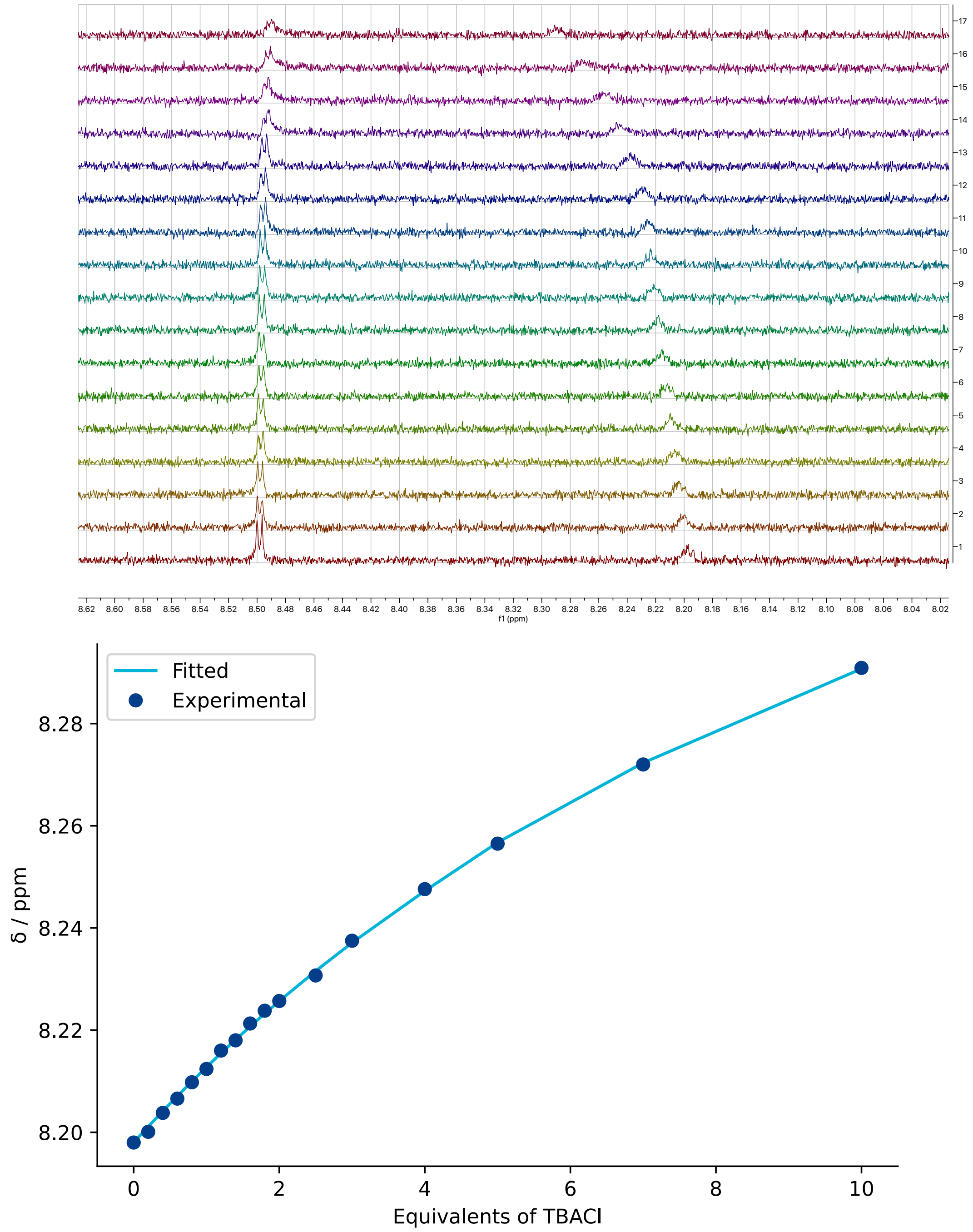

Figure S51. Top: Truncated ^{1}H NMR spectra of **6** upon titration with TBACl in 2.5% D_2O/acetone-d6 (v/v) from 0 to 10 equivalents. Bottom: Corresponding Cl^- binding isotherm. Experimental data shown by ●, fitted 1:1 binding isotherm shown by solid line.

5. Predicted cLogP Values of Transporters

Table S1: Consensus cLogP values from Swiss ADME.[12]

	1	2	3	4	5	6
Consensus cLogP	7.08	4.84	5.42	6.01	6.55	6.62

6. Product Ratios in Macrocyclisation of 4 and 5

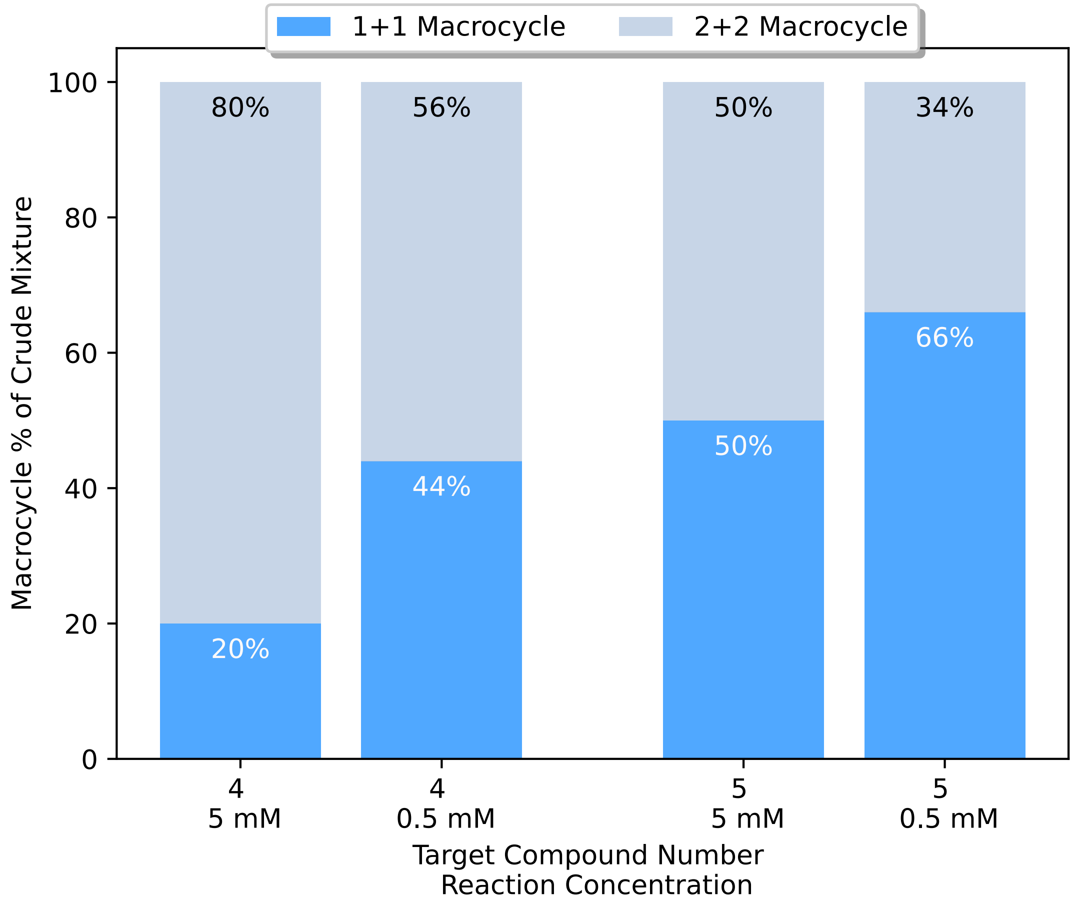

Figure S52. Plot of ratios of desired monomeric [1+1] cyclisation products, where a single bis-azide and bis-iodoalkyne form a macrocycle, and the undesired [2+2] oligomers in the reaction mixture, whose identity was confirmed by high resolution mass spectroscopy. The relative amounts of each product were determined from NMR integration of aromatic protons in the product mixture, prior to reverse phase chromatographic separation.

7. DFT Calculations

Using the crystal structure of **1** as a starting point (CCDC deposition number 2075766)[4], initial structures of **2-6** were generated in Avogadro (version 1.20), and optimised using the built-in General Amber Force-Field, followed by optimisation using XTB (version 6.4.1).[13–15] These structures were used as the starting points for the conformer searching algorithm (scheme **S1**). In the first step, a conformer search was carried out using the CREST program (version 2.11) in NCI mode using the default metadynamics parameters.[16] From the conformers generated, forty conformers of lowest energy were selected for single point energy calculations in ORCA (version 5.0.3) at the ωB97X-D3/def2-TZVP level of theory to improve the reliability of their energy ranking.[17–20] The lowest energy conformers following this re-ranking were used in all subsequent DFT calculations. The coordinates of the final optimised structures can be found in the public GitHub repository https://github.com/martinzola/chloride_selective_non-protonophoric_ion_transport.

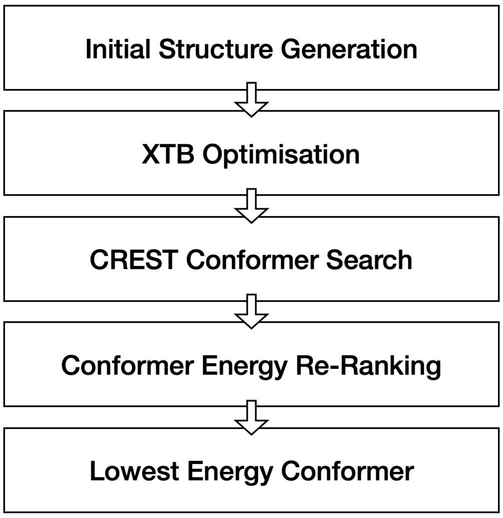

Scheme S1. Conformer searching and sorting workflow: Generation of transporter structure from crystal structure of **1** in Avogadro; Optimisation using XTB; Conformer search using metadynamics in CREST; Re-ranking of 40 lowest-energy conformers at the ωB97X-D3/def2-TZVP in ORCA giving the lowest energy conformer.

Optimisations and energy calculations were carried out with the ORCA suite of programs (version 5.0.3), with the ωB97X-D3 functional chosen to match that used in our previous studies on related systems.[4,6] The choice was previously motivated by benchmarking studies showing that the ωB97X family of functionals outperforms most other functionals in reproducing coupled cluster and MP2-level energies of halogen bonding interactions.[21] Lowest energy conformers of the transporters were optimised at the SMD($CHCl_3$)-ωB97X-D3/def2-SVP level of theory (ma-def2-SVP on underlined atoms: I, Cl^- and OH), referred here to as Low-Level (**LL**), followed by a frequency calculation at the same level.[19,20,22] Very tight convergence criteria were employed (2 ∗ 10−7 Ha for the optimisation step and 10−8 Ha for the SCF energy change). The resolution of identity approximation and chain of sphere integration (RIJCOSX keyword) were used to speed up calculations, using the default auxiliary basis sets.[23] Thermochemistry was calculated using the otherm program, using the default quasi-RRHO approximation ($\omega_0 = 45\ cm^{-1}$, $\alpha = 4$, $T = 298.15$ K).[24] By default, the otherm programme corrects the standard state from 1 atm to 1 M, which adds 1.89 kcal mol^{-1} (equivalent to RT·ln(1 mol dm^{-3}/ (1/24.5 mol dm^{-3}))) e.g. [$G_{\mathbf{HL},1M} = G_{\mathbf{HL},1atm} + RTln(24.5)$]. Single point energies at a higher level (**HL**) were calculated at the CPCM($CHCl_3$)-ωB97X-D3/def2-QZVP (ma-def2-QZVP on underlined atoms: I, Cl^- and OH), level of theory. To approximate the free energies at this level, the thermodynamic contributions from the **LL** level

were added to the single point energies at the **HL** level, i.e. $G_{HL} = E_{HL} + (G_{LL} - E_{LL})$. The binding interaction is then calculated as the energy difference of the complex [**Transporter**·Cl]$^-$ or [**Transporter**·OH]$^-$ and the energy of the separated **Transporter** and Cl^-/OH^-.

Additionally, in order to ascertain the balance between stabilising and destabilising interactions in all of the studied complexes, distortion interaction analysis was carried out by carrying out frequency calculations at the **LL** level and single point calculations at the **HL** level of theory on the geometries of each transporter with the host ion removed.[25] The difference between the energies of the complex in its bound pose and its minimal energy pose is the distortion energy, and quantifies how much energy must be input into the reorganisation of the molecule in order for an interaction to take place. The difference between this distortion energy and the overall binding energy is then the interaction energy, which shows how strongly the molecule is able to interact with its guest in its bound conformation.

We also carried out second order perturbation analysis using NBO 7.0 in ORCA for all [transporter ·ion]$^-$ complexes at the **HL** level of theory to give interaction energies between the ions and the halogen bonding iodines We report the sum of all interaction energies $E^{(2)}$ between lone pairs on the ions and the anti-bonding orbitals of the bond between the halogen bonding iodine and the C4 carbon of the iodotriazole to which it is attached.

Energy details of Calculations

Table S2. Complete details of computational calculations, including electronic energy (E_{el}), the zero point energy correction (ZPE), enthalpy (H), quasi-RRHO entropy contribution at 298.1 K (Tqh-S) and the total correction to the electronic energy giving the quasi-RRHO Gibbs free energy (qh-G) acalculated at the SMD($CHCl_3$)-ωB97X-D3/def2-SVP level of theory (**LL**) or SMD($CHCl_3$)-ωB97X-D3/def2-QZVP// ωB97X-D3/def2-SVP level of theory (**HL**/**LL**). Free energies were calculated at 298.1K and 1M. All values are in units of Eh.

	LL						HL/LL		
**Species**	E_{el}	ZPE	H	Tqh-S	Total Corr	qh-G	E_{el}	qh-G	qh-H
Cl-	-460.22072	0	-460.21694	0.01438387	-0.0106071	-460.231323	-460.38087	-460.39148	-460.37709
OH-	-75.819587	0.00886066	-75.816283	0.0159421	-0.0126387	-75.8322248	-75.910971	-75.92361	-75.907666
**1**	-2965.1694	0.24568217	-2964.8866	0.10176492	0.18096209	-2964.9884	-2968.0331	-2967.8521	-2967.7503
[**1**·Cl]-	-3425.4138	0.24641	-3425.1284	0.1064108	0.1790341	-3425.23479	-3428.4364	-3428.2574	-3428.151
[**1**·OH]-	-3041.0369	0.2577583	-3040.7399	0.1053485	0.1916165	-3040.84526	-3043.9893	-3043.7976	-3043.6923
**2**	-2134.6947	0.51295429	-2134.1469	0.09390235	0.45394509	-2134.24075	-2136.4595	-2136.0056	-2135.9117
[**2**·Cl]-	-2594.931	0.51511877	-2594.3802	0.09869109	0.45211907	-2594.47884	-2596.8578	-2596.4057	-2596.307
[**2**·OH]-	-2210.5512	0.5284973	-2209.9873	0.0963384	0.46751872	-2210.08368	-2212.4078	-2211.9403	-2211.8439
**3**	-2213.2513	0.57280416	-2212.6428	0.09819906	0.51029335	-2212.74097	-2215.1041	-2214.5938	-2214.4956
[**3**·Cl]-	-2673.4918	0.57298782	-2672.8808	0.10336296	0.50767172	-2672.98417	-2675.5037	-2674.996	-2674.8926
[**3**·OH]-	-2289.1127	0.58562528	-2288.4894	0.10137526	0.52196516	-2288.59077	-2291.0546	-2290.5326	-2290.4313
**4**	-2531.22	0.48219671	-2530.7007	0.10082519	0.41854729	-2530.80149	-2533.4921	-2533.0736	-2532.9728
[**4**·Cl]-	-2991.4608	0.48237436	-2990.939	0.10646514	0.41538964	-2991.04544	-2993.8919	-2993.4765	-2993.3701
[**4**·OH]-	-2607.0816	0.49568099	-2606.5467	0.10412265	0.43079311	-2606.65081	-2609.442	-2609.0112	-2608.9071

Table S3. Complete details of computational calculations, including electronic energy (E_{el}), the zero point energy correction (ZPE), enthalpy (H), quasi-RRHO entropy contribution at 298.1 K (Tqh-S) and the total correction to the electronic energy giving the quasi-RRHO Gibbs free energy (qh-G) calculated at the SMD($CHCl_3$)-ωB97X-D3/def2-SVP level of theory (**LL**) or SMD($CHCl_3$)-ωB97X-D3/def2-QZVP// ωB97X-D3/def2-SVP level of theory (**HL**/**LL**). Free energies were calculated at 298.1K and 1M. All values are in kcal mol^{-1}.

	LL						HL/LL		
**Process**	E_{el}	ZPE	H	Tqh-S	Total Corr	qh-G	E_{el}	qh-G	qh-H
**1** + Cl- → [**1**·Cl]-	-14.9	0.5	-15.6	-6.1	5.4	-9.5	-14.1	-8.7	-14.8
**1** + OH- → [**1**·OH]-	-30.1	2.0	-23.2	-7.8	14.6	-15.5	-28.4	-13.8	-21.5
2 + Cl- → [2·Cl]-	-9.8	1.4	-10.3	-6.0	5.5	-4.2	-10.9	-5.4	-11.4
2 + OH- → [2·OH]-	-23.2	4.2	-15.2	-8.5	16.4	-6.7	-23.4	-6.9	-15.4
3 + Cl- → [3·Cl]-	-12.5	0.1	-13.2	-5.8	5.0	-7.4	-11.7	-6.7	-12.5
3 + OH- → [3·OH]-	-26.3	2.5	-19.0	-8.0	15.3	-11.0	-24.8	-9.6	-17.6
4 + Cl- → [4·Cl]-	-12.6	0.1	-13.4	-5.5	4.7	-7.9	-11.9	-7.2	-12.7
4 + OH- → [4·OH]-	-26.3	2.9	-18.7	-7.9	15.6	-10.7	-24.4	-8.8	-16.7

Table S4. Details of single point calculations of transporters distorted to their ion-binding poses with the anion removed, showing the electronic energy (E_{el}) for each transporter in its [transporter·ion]$^-$ binding pose. The energies were calculated at the ωB97X-D3/def2-SVP level of theory (**HL**). All values are in units of Eh.

	**HL**
	E_e
**1** in pose of [**1**·Cl]-	-2968.0309
**1** in pose of [**1**·OH]-	-2968.0235
**2** in pose of [**2**·Cl]-	-2136.4555
**2** in pose of [**2**·OH]-	-2136.4491
**3** in pose of [**3**·Cl]-	-2215.101
**3** in pose of [**3**·OH]-	-2215.0931
**4** in pose of [**4**·Cl]-	-2533.4905
**4** in pose of [**4**·OH]-	-2533.4833

Table S5. Details of distortion-interaction calculations of transporters distorted to their ion-binding poses with the anion removed. The distortion energy or each transporter is the energy required to distort the transporter to its [transporter·ion]$^-$ binding pose, and the interaction energy is the process of the ion binding to the distorted transporter. The energies were calculated at the ωB97X-D3/def2-SVP level of theory (**HL**). All values are in units of kcal mol^{-1}.

	**HL**	
	$E_{distortion}$	$E_{interaction}$
**1** in pose of [**1**·Cl]-	1.3	-15.5
**1** in pose of [**1**·OH]-	6.0	-34.4
**2** in pose of [**2**·Cl]-	2.6	-13.5
**2** in pose of [**2**·OH]-	6.5	-29.8
**3** in pose of [**3**·Cl]-	1.9	-13.7
**3** in pose of [**3**·OH]-	6.9	-31.7
**4** in pose of [**4**·Cl]-	1.0	-12.9
**4** in pose of [**4**·OH]-	5.5	-29.9

Table S6. Partition of vibrational energy between the vibrational, rotational and translational contributions for optimised structures of all species at their energy minima. The frequency calculations were carried out at the SMD($CHCl_3$)-ωB97X-D3/def2-SVP level of theory (**LL**), and the vibrational energies calculated using Grimme's quasi-RRHO approximation in otherm (using parameters ω_0 = 45 cm^{-1}, α = 4, T = 298.15 K).[24,26] Free energies were calculated at 298.1K and 1M. All values are in Eh.

	$S_{vibrational}$	$S_{rotational}$	$S_{translational}$
Cl-	0	0	0.01438342
OH-	0	0.00259858	0.01334352
**1**	0.06385005	0.01904634	0.01886851
[**1**·Cl]-	0.0686549	0.01899347	0.01892698
[**1**·OH]-	0.06750316	0.01894841	0.01889686
**2**	0.05706642	0.01818101	0.01865491
[**2**·Cl]-	0.06166911	0.01829929	0.01872268
[**2**·OH]-	0.05942489	0.01822567	0.01868782
**3**	0.06100278	0.01848747	0.0187088
[**3**·Cl]-	0.06616269	0.01842617	0.01877409
[**3**·OH]-	0.06427021	0.01836454	0.01874049
**4**	0.06344561	0.01859035	0.01878923
[**4**·Cl]-	0.06914003	0.0184741	0.018851
[**4**·OH]-	0.06689737	0.01840606	0.0188192

Table S7. Partition of vibrational energy between the vibrational, rotational and translational for the binding of **1-4** to Cl^- and OH^-. The frequency calculations were carried out at the SMD($CHCl_3$)-ωB97X-D3/def2-SVP level of theory (**LL**), and the vibrational energies calculated using Grimme's quasi-RRHO approximation in otherm (using parameters ω_0 = 45 cm^{-1}, α = 4, T = 298.15 K).[24,26] Free energies were calculated at 298.1K and 1M. All values are in Eh.

**Process**	$\Delta S_{vibrational}$	$\Delta S_{rotational}$	$\Delta S_{translational}$
**1** + Cl- → [**1**·Cl]-	3.0	0.0	-9.0
**1** + OH- → [**1**·OH]-	2.3	-1.7	-8.4
2 + Cl- → [2·Cl]-	2.9	0.1	-9.0
2 + OH- → [2·OH]-	1.5	-1.6	-8.3
3 + Cl- → [3·Cl]-	3.2	0.0	-9.0
3 + OH- → [3·OH]-	2.0	-1.7	-8.3
4 + Cl- → [4·Cl]-	3.6	-0.1	-9.0
4 + OH- → [4·OH]-	2.2	-1.7	-8.3

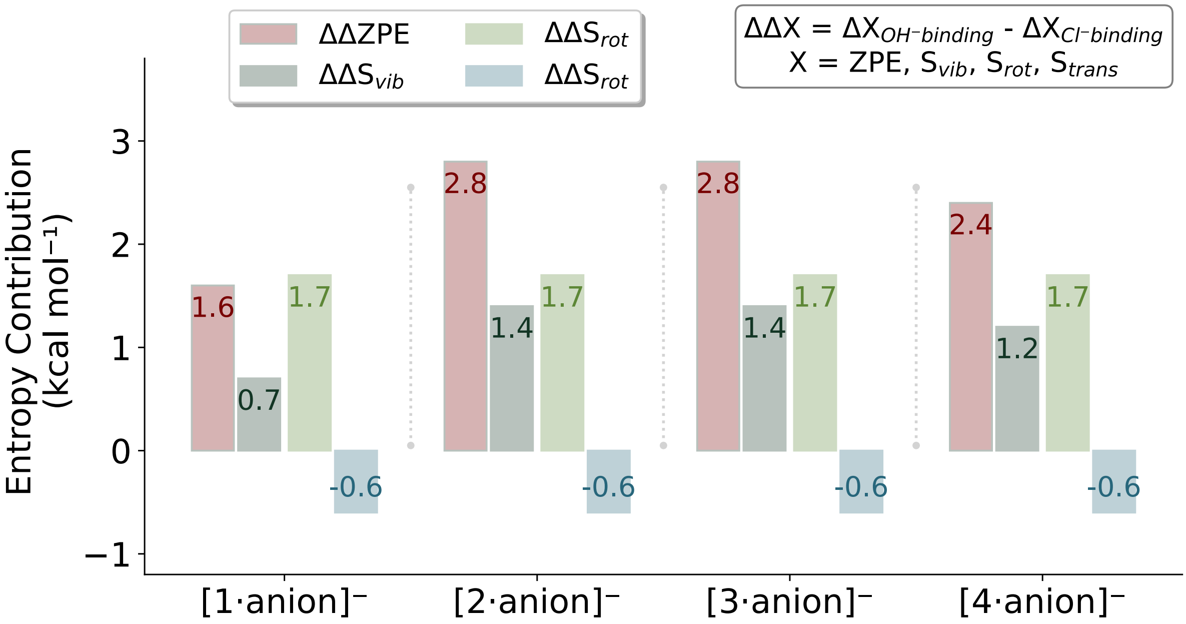

Figure S53. Differences in the zero point vibrational energy and entropic contribution upon ion binding for each transporter, partitioned into differences in vibrational, rotational and translational contributions. Negative values indicate OH^- preference.

Minimum Energy Structures of Transporters and Their Complexes

All structures were visualised using Pymol software, with the following colours being assigned to each element: H (white), C (gray), N (blue), O (red), F (teal), I (purple).[27]

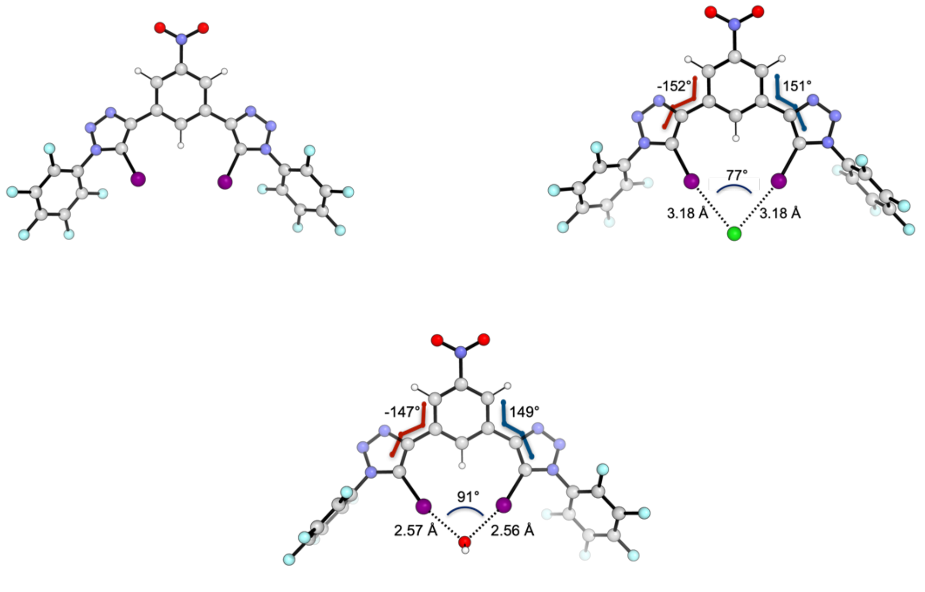

Figure S54. Structure of **1**, [**1**·Cl]⁻ and [**1**·OH]⁻ optimised at the SMD($CHCl_3$)-ωB97X-D3/def2-SVP level of theory (ma-def2-SVP on I), highlighting the distances of the intermolecular interaction.

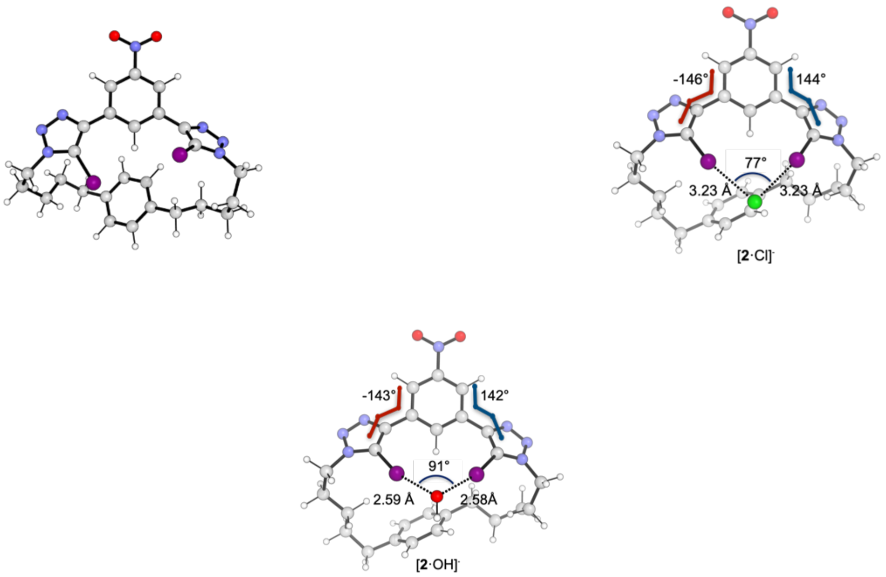

Figure S55. Structure of **2**, [**2**·Cl]$^-$ and [**2**·OH]$^-$ optimised at the SMD($CHCl_3$)-ωB97X-D3/def2-SVP level of theory (ma-def2-SVP on I), highlighting the distances of the intermolecular interaction.

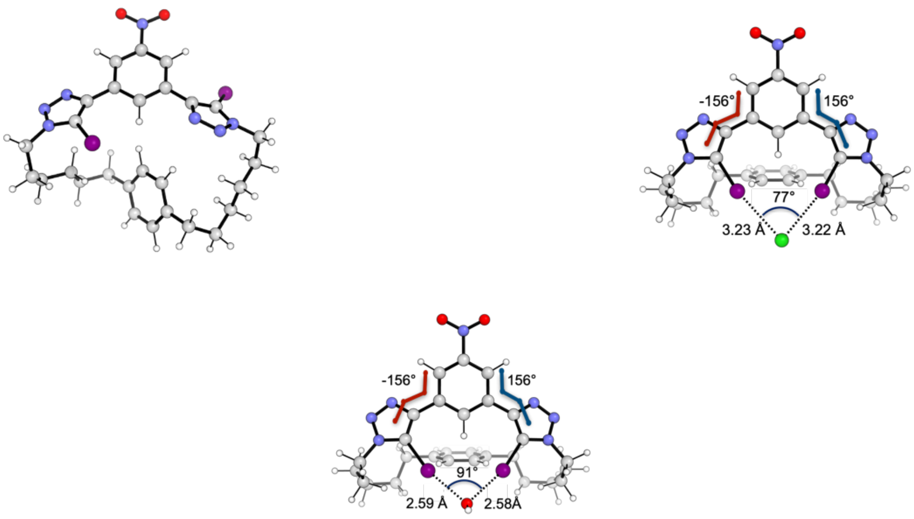

Figure S56. Structure of **3**, [**3**·Cl]$^-$ and [**3**·OH]$^-$ optimised at the SMD($CHCl_3$)-ωB97X-D3/def2-SVP level of theory (ma-def2-SVP on I), highlighting the distances of the intermolecular interaction.

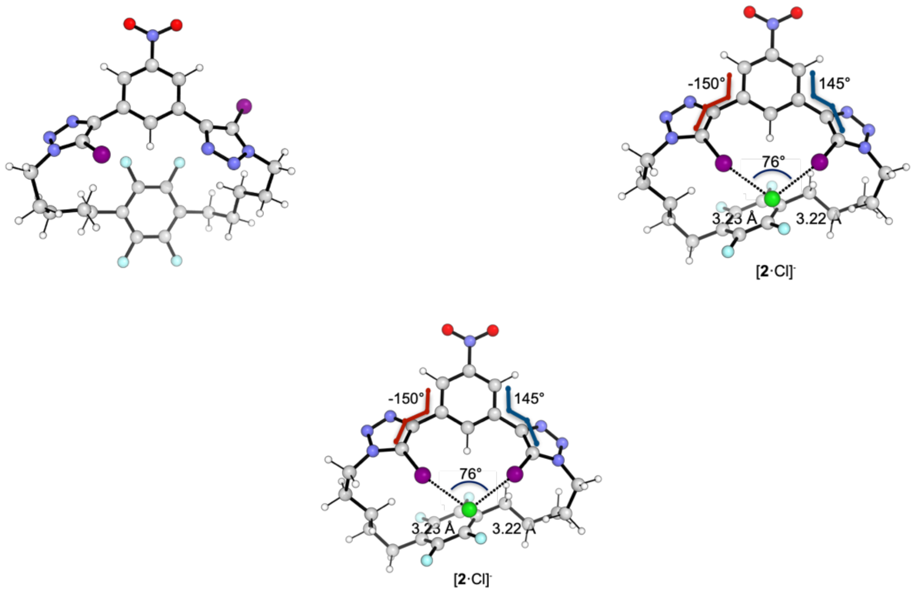

Figure S57. Structure of **4**, [**4**·Cl]$^-$ and [**4**·OH]$^-$ optimised at the SMD($CHCl_3$)-ωB97X-D3/def2-SVP level of theory (ma-def2-SVP on **I**), highlighting the distances of the intermolecular interaction.

8. Molecular Dynamics

To obtain the structures for fitting of the electrostatic parameters for molecular dynamics simulations, the five lowest energy conformers of transporters **1** and **2** found through the conformer searching workflow (cf. **Scheme S1**) were optimised at the **LL** level of theory (cf. DFT Calculations). The electrostatic potential calculations were carried out in Gaussian 09 at the HF/6-31G* level of theory, using the Merz-Singh-Kollman scheme for charge calculation with 6 density points in each layer (IOp(6/33=2, 6/42=6, 6/50=1)).[28,29] RESP fitting was then employed using the antechamber suite with equal weighting of all five conformers, alongside the assignment of GAFF atom types.[14,30,31] The sigma hole was parameterised as a positively charged particle with no mass, angled 180 ° away from the carbon-iodine bond, according to literature precedent.[32,33] The distance of the extra particle from the iodine atom's center used in the RESP fitting and the Lennard-Jones distance parameter σ were then altered until DFT binding distances could be approximated, without compromising the stability of simulations. Molecular mechanics interaction distances between **1** and Cl^-, obtained by the optimising 10,000 steps in the steepest descent algorithm, were compared to those in the **LL** level of theory for the default and optimised sigma hole and Lennard-Jones parameters (**Table S8**). DMSO parameters were taken from Caleman et al, chloride parameters from Sengupta et al, hydroxide parameters from Han et al, and default AMBER parameters were used for sodium.[34–37] For POPC, the parameters used were the Stockholm lipids (Slipids-2020).[38,39] Water was modelled using the tip3p-fb model.[40] GAFF parameters for the simulated compounds, as well as the parameter files and starting GROMACS geometries can be found in the public GitHub repository https://github.com/martinzola/chloride_selective_non-protonophoric_ion_transport.

Table S8. Standard parameters and final optimised parameters of extra point fitting, Iodine Lennard-Jones parameter σ, the corresponding AMBER Lennard-Jones R_{min} in Amber units (conversion factor of $10 \cdot 2^{1/6}$), as well as the corresponding I-Cl⁻ distance in the [**2**·Cl]⁻.

	GROMACS Iodine σ (nm)	AMBER Iodine R_{min} (Å)	EP distance from I (Å)	MM I-Cl⁻ distance (Å)	DFT reference I-Cl⁻ distance (Å)
Initial Parameters	0.383	4.30	2.15	3.87	3.23
Optimised Parameters	0.320	3.60	1.60	3.36	

Throughout all simulations, long-range electrostatics were described with the Particle Mesh Ewald (PME) algorithm.[41] The temperature of the system was maintained at 298 K using the V-rescale thermostat.[42] Pressure was controlled by the C-rescale barostat at 1.0 bar, with an isothermal compressibility of $4.5 \cdot 10\text{-}5$ bar^{-1}.[43] In the case of the membrane simulations, the pressure was controlled in a semi-isotropic manner, where $P_{xy} = P_x = P_y$ is the pressure in the plane of the membrane and P_z is the pressure in the direction of the membrane normal. All bond lengths involving hydrogen atoms were constrained using the LINCS algorithm.[44]I

Simulations were carried out in the GROMACS simulation software package (v.2021.3 in all subsequent mentions).[45] Plumed (v.2.7.2) was used to apply biasing forces in GROMACS for metadynamics simulations.[46]

Metadynamics in DMSO

The structure of **2** was inserted into a cubic box with a 1 nm buffer to the edge of the box and solvated with DMSO. The systems were minimised using a steepest descent algorithm until the maximum force in the system was below 1000 kJ mol-1. This was followed by 500 ps of equilibration in the NPT ensemble (2 fs timestep, 298 K, 1 bar) starting from random velocities sampled from the Maxwell-Boltzmann distribution.

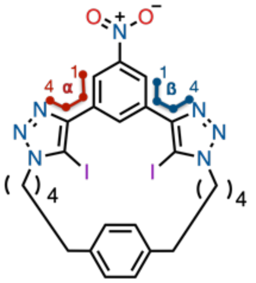

Scheme S2. Structure of macrocyclic transporter **2**, highlighting dihedral angles α and β between the iodotriazole rings and the central nitrobenzene ring (specified in the direction 1 -> 4).

The equilibrated system was then simulated for 1.2 μs, until convergence was found in the free energy profile (Figure S58), employing the well-tempered metadynamics (WTMetaD) method using Plumed. The pace of gaussian hill was set to 500 steps and the initial height of the hills was set to 0.3 kJ mol^{-1}. The sigma values of the gaussian hills were set to 0.3 radians for both α and β angles, respectively, and the bias factor was set to 6. To speed up the calculations, the biases were stored on a grid ranging from -π to π for each angle, with the default grid spacing.

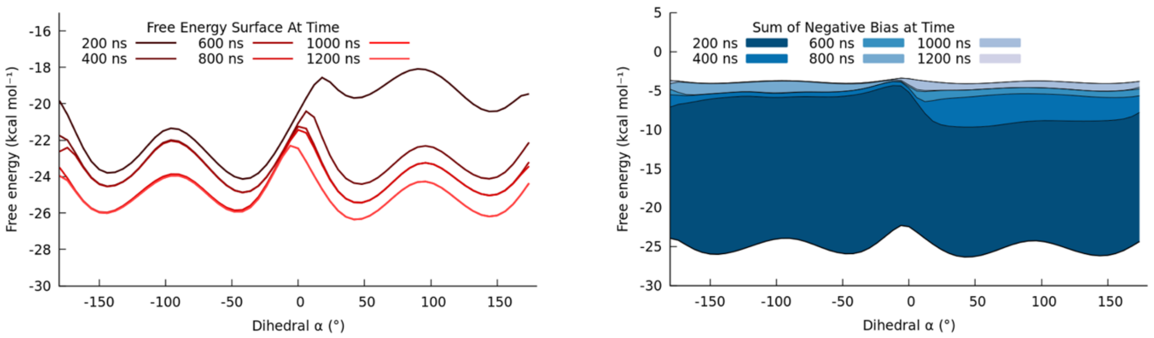

Figure S58. Left: A plot of the convergence of the 2D free energy surface in a single dihedral projection (dihedral angle α) through the course of the 1.2 μs simulation. Right: Plot of the cumulative negative bias deposition over time, along dihedral angle α during the same simulation. Metadynamics parameters: hill deposition pace of 500 steps, hill size of 0.3 kJ mol^{-1}, sigma of 0.3 radians, bias factor 6.

To assess the convergence of the WTMetaD simulations, the evolution of the potential energy surface along a single dimension (dihedral angle α, Figure S58) was monitored during the 1.2 μs. This showed that hysteresis was greatly reduced after a period of 1 μs and no further changes were observed in the profile, justifying that this simulation was converged.

Rate constants were estimated using the Eyring equation (**S3**), where dG was the activation barrier from metadynamics simulations and the transition coefficient κ was taken to be equal to 1, k_b is Boltzmann's constant, T is 298 K and h is Planck's constant and R is the gas constant. The half-life $t_{1/2}$ was then calculated using equation **S4**.

$$k = \kappa \frac{k_b T}{h} e^{\frac{-dG}{RT}} \qquad (\mathbf{S3})$$

$$t_{1/2} = \frac{\ln(2)}{k} \qquad (\mathbf{S4})$$

To verify the free energy surface obtained using metadynamics simulations the relative energies of the four conformational minima were benchmarked against their DFT energies. The starting points for the DFT optimisations were the four lowest energy conformers from the crest conformational search for **2**, which had the representative dihedral angles found in each minimum well (main Figure 2). They were optimised using the **LL** level of theory followed by single point calculations at the **HL** level of theory (Table S9). Relative energies of these conformers found by DFT were then compared to the relative energies of the four conformer minimum wells found by Metadynamics (Table S10).

Table S9. Absolute energies (in Hartrees) for conformers of **2**, including electronic energy (E_{el}), zero point energy correction (ZPE), enthalpy (H), quasi-RRHO entropy contribution at 298.1 K (Tqh-S) and the total correction to the electronic energy giving the quasi-RRHO Gibbs free energy (qh-G) calculated at the SMD(DMSO)-ωB97X-D3/def2-SVP level of theory (**LL**) or SMD(DMSO)-ωB97X-D3/def2-QZVP// ωB97X-D3/def2-SVP level of theory (**HL**/**LL**). Free energies were calculated at 298.1K and 1M.

	**LL**						**HL/LL**		
**Species**	E_{el}	ZPE	H	Tqh-S	Total Corr	qh-G	E_{el}	qh-G	qh-H
trans-A	-2134.682296	0.514045	-2134.134796	0.090460	0.457040	-2134.225256	-2136.450604	-2135.993564	-2135.903104
trans-B	-2134.687361	0.514427	-2134.139421	0.090497	0.457443	-2134.229918	-2136.453524	-2135.996081	-2135.905584
cis-C	-2134.685396	0.515098	-2134.137005	0.089910	0.458481	-2134.226915	-2136.452062	-2135.993582	-2135.903671
cis-D	-2134.684419	0.514252	-2134.136482	0.090541	0.457397	-2134.227022	-2136.452189	-2135.994792	-2135.904251

Table S10. Comparison of relative Gibbs free energies of conformers of **2**, using the **HL/LL** data from **table S5** as the DFT energies, and using the output of the WTMetaD as the MD energies. All values are in units of kcal mol^{-1}.

**Conformer**	dG_{DFT}	dG_{MD}
A	1.6	1.3
B	Minimum (dG = 0)	Minimum (dG = 0)
C	1.6	0.1
D	0.8	1.1
Mean Absolute Error	± 0.5	

Water Simulations

The [**transporter**·ion]$^-$ complexes or the lone ions were placed in a cubic simulation box with a 1 nm buffer to the edge of the box and solvated with water. The same 2000 kj mol^{-1} nm^{-2} harmonic restraint was placed between the iodines and the respective ion of the complex for the duration of all simulations. The systems were minimised using a steepest descent algorithm until the maximum force in the system was below 1000 kJ mol-1. This was followed by 500 ps of equilibration in the NPT ensemble (2 fs timestep, 298 K, 1 bar) starting from random velocities sampled from the Maxwell-Boltzmann distribution. Production simulations were run for 100 ns, saving geometries every 10 ps. Radial distribution functions were calculated from these simulations using the rdf utility of GROMACS, using the transporter-bound ion and all water oxygens as the two reference groups, and a bin width of 0.05 Å.

In all cases, the average number of waters in the ion's first hydration shell was taken as the cumulative radial distribution up to the first minimum. In the case of the hydroxide ions, this was found to be at 3.7 Å, and in the case of the chloride ions, this was at 4.1 Å.

Membrane Simulations

Procedures for membrane simulations were adapted from literature precedent.[47–49] In the simulations of the complexes of **1** and **2** with Cl^- and OH^-, the RESP fitting was conducted with identical parameters as previously, with the only change being that the entire complex, including the ions, was now fitted together. This allowed the some of the charge from the ions to be transferred onto the transporter. A [**transporter**·ion]$^-$ complex consisting of **1** or **2** complexed to either Cl^- or OH^- was placed in a cavity in the centre of a pre-equilibrated POPC membrane containing 138 lipids and 5600 water molecules. Throughout the entirety of the simulations, a harmonic distance restraint was imposed between both iodines and the respective ion, with the distance equal to the interaction distance found in the DFT calculations at the **LL** level of theory (**Figures S50-62**), with a force constant of 2000 kJ mol^{-1} nm^{-2}. The system was neutralised with Na^+ and brought up to a 0.1 M concentration of NaCl. The systems were then minimised using the steepest descent algorithm until the maximum force was below 1000 kj mol^{-1} nm^{-1}, applying a 1000 kj mol^{-1} nm^{-2} restraint on the heavy atoms of the [**transporter**·ion]$^-$ complex. The systems then equilibrated for 500 ps in the NPT ensemble (2 fs timestep, 298 K, 1 bar) starting from random velocities sampled from the Maxwell-Boltzmann distribution, with a position restraint of 1000 kj mol^{-1} nm^{-2} continuing to be applied on the heavy atoms of the [**transporter**·ion]$^-$ complex. The [**transporter**·ion]$^-$ complex was then allowed to move to its equilibrium position at the membrane-water interface in a further 5 ns long NPT equilibration. Three repeats of 100 ns production simulations were then carried out at this equilibrium position in the NPT ensemble, saving geometries every 10 ps. Radial distribution functions were calculated from these simulations using the rdf utility of GROMACS, using the transporter-bound ion and all water oxygens as the two reference groups, and a bin width of 0.05 Å.

RDFs of Water Around Ions In the Solution and in the Membrane

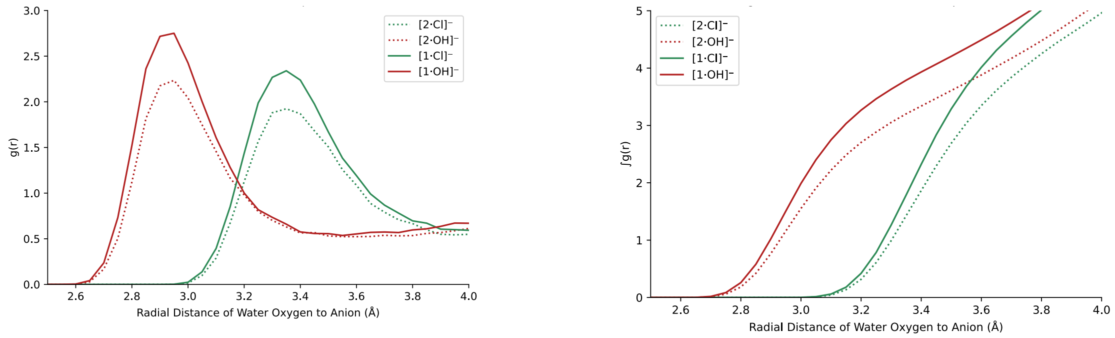

Figure S59. Left: Radial distribution function of water around the anion for the four different [**transporter**·ion]$^{-}$complexes in TIP3P-FB water. Right: Cumulative radial distribution function of water around the anion for the four different [**transporter**·ion]$^{-}$complexes in TIP3P-FB water.

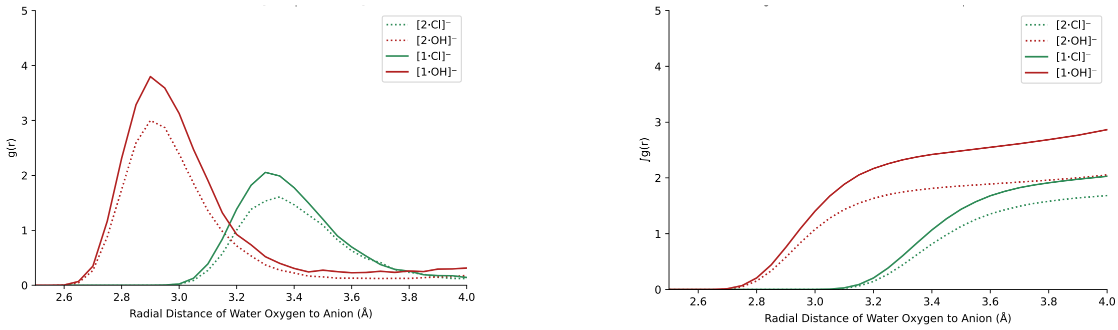

Figure S60. Left: Radial distribution function of water around the anion for the four different [**transporter**·ion]$^{-}$complexes at their membrane-water interface equilibrium position. Right: Cumulative radial distribution function of water around the anion for the four different [**transporter**·ion]$^{-}$complexes at their membrane-water interface equilibrium position.

References

(1) Brassard, C. J.; Zhang, X.; Brewer, C. R.; Liu, P.; Clark, R. J.; Zhu, L. Cu(II)-Catalyzed Oxidative Formation of 5,5′-Bistriazoles. *J. Org. Chem.* **2016**, *81* (24), 12091–12105. https://doi.org/10.1021/acs.joc.6b01907.

(2) Charoenpattarapreeda, J.; Tan, Y. S.; Iegre, J.; Walsh, S. J.; Fowler, E.; Eapen, R. S.; Wu, Y.; Sore, H. F.; Verma, C. S.; Itzhaki, L.; Spring, D. R. Targeted Covalent Inhibitors of MDM2 Using Electrophile-Bearing Stapled Peptides. *Chem. Commun.* **2019**, *55* (55), 7914–7917. https://doi.org/10.1039/C9CC04022F.

(3) Hein, J. E.; Tripp, J. C.; Krasnova, L. B.; Sharpless, K. B.; Fokin, V. V. Copper(I)-Catalyzed Cycloaddition of Organic Azides and 1-Iodoalkynes. *Angewandte Chemie International Edition* **2009**, *48* (43), 8018–8021. https://doi.org/10.1002/anie.200903558.

(4) Bickerton, L. E.; Docker, A.; Sterling, A. J.; Kuhn, H.; Duarte, F.; Beer, P. D.; Langton, M. J. Highly Active Halogen Bonding and Chalcogen Bonding Chloride Transporters with Non-

Protonophoric Activity. *Chemistry – A European Journal* **2021**, *27* (45), 11738–11745. https://doi.org/10.1002/chem.202101681.
(5) Neenan, T. X.; Whitesides, G. M. Synthesis of High Carbon Materials from Acetylenic Precursors. Preparation of Aromatic Monomers Bearing Multiple Ethynyl Groups. *J. Org. Chem.* **1988**, *53* (11), 2489–2496. https://doi.org/10.1021/jo00246a018.
(6) Bickerton, L. E.; Sterling, A. J.; Beer, P. D.; Duarte, F.; Langton, M. J. Transmembrane Anion Transport Mediated by Halogen Bonding and Hydrogen Bonding Triazole Anionophores. *Chem. Sci.* **2020**, *11* (18), 4722–4729. https://doi.org/10.1039/D0SC01467B.
(7) Luca, V.; Tejada, J. J.; Vega, D.; Arrachart, G.; Rey, C. Zirconium(IV)–Benzene Phosphonate Coordination Polymers: Lanthanide and Actinide Extraction and Thermal Properties. *Inorg. Chem.* **2016**, *55* (16), 7928–7943. https://doi.org/10.1021/acs.inorgchem.6b00954.
(8) Sun, Y.; Li, S.; Zhou, Z.; Saha, M. L.; Datta, S.; Zhang, M.; Yan, X.; Tian, D.; Wang, H.; Wang, L.; Li, X.; Liu, M.; Li, H.; Stang, P. J. Alanine-Based Chiral Metallogels via Supramolecular Coordination Complex Platforms: Metallogelation Induced Chirality Transfer. *J. Am. Chem. Soc.* **2018**, *140* (9), 3257–3263. https://doi.org/10.1021/jacs.7b10769.
(9) Busschaert, N.; Elmes, R. B. P.; Czech, D. D.; Wu, X.; Kirby, I. L.; Peck, E. M.; Hendzel, K. D.; Shaw, S. K.; Chan, B.; Smith, B. D.; Jolliffe, K. A.; Gale, P. A. Thiosquaramides: PH Switchable Anion Transporters. *Chem. Sci.* **2014**, *5* (9), 3617–3626. https://doi.org/10.1039/C4SC01629G.
(10) Jentzsch, A. V.; Emery, D.; Mareda, J.; Nayak, S. K.; Metrangolo, P.; Resnati, G.; Sakai, N.; Matile, S. Transmembrane Anion Transport Mediated by Halogen-Bond Donors. *Nat Commun* **2012**, *3* (1), 905. https://doi.org/10.1038/ncomms1902.
(11) *BindFit v0.5 | Supramolecular*. http://app.supramolecular.org/bindfit/ (accessed 2024-09-23).
(12) Daina, A.; Michielin, O.; Zoete, V. SwissADME: A Free Web Tool to Evaluate Pharmacokinetics, Drug-Likeness and Medicinal Chemistry Friendliness of Small Molecules. *Sci Rep* **2017**, *7* (1), 42717. https://doi.org/10.1038/srep42717.
(13) Hanwell, M. D.; Curtis, D. E.; Lonie, D. C.; Vandermeersch, T.; Zurek, E.; Hutchison, G. R. Avogadro: An Advanced Semantic Chemical Editor, Visualization, and Analysis Platform. *J Cheminform* **2012**, *4* (1), 17. https://doi.org/10.1186/1758-2946-4-17.
(14) Wang, J.; Wolf, R. M.; Caldwell, J. W.; Kollman, P. A.; Case, D. A. Development and Testing of a General Amber Force Field. *Journal of Computational Chemistry* **2004**, *25* (9), 1157–1174. https://doi.org/10.1002/jcc.20035.
(15) Bannwarth, C.; Ehlert, S.; Grimme, S. GFN2-XTB—An Accurate and Broadly Parametrized Self-Consistent Tight-Binding Quantum Chemical Method with Multipole Electrostatics and Density-Dependent Dispersion Contributions. *J. Chem. Theory Comput.* **2019**, *15* (3), 1652–1671. https://doi.org/10.1021/acs.jctc.8b01176.
(16) Grimme, S. Exploration of Chemical Compound, Conformer, and Reaction Space with Meta-Dynamics Simulations Based on Tight-Binding Quantum Chemical Calculations. *J. Chem. Theory Comput.* **2019**, *15* (5), 2847–2862. https://doi.org/10.1021/acs.jctc.9b00143.
(17) Neese, F. The ORCA Program System. *WIREs Comput Mol Sci* **2012**, *2* (1), 73–78. https://doi.org/10.1002/wcms.81.
(18) Neese, F. Software Update: The ORCA Program System—Version 5.0. *WIREs Comput Mol Sci* **2022**, *12* (5), e1606. https://doi.org/10.1002/wcms.1606.
(19) Chai, J.-D.; Head-Gordon, M. Long-Range Corrected Hybrid Density Functionals with Damped Atom–Atom Dispersion Corrections. *Phys. Chem. Chem. Phys.* **2008**, *10* (44), 6615. https://doi.org/10.1039/b810189b.
(20) Weigend, F.; Ahlrichs, R. Balanced Basis Sets of Split Valence, Triple Zeta Valence and Quadruple Zeta Valence Quality for H to Rn: Design and Assessment of Accuracy. *Phys. Chem. Chem. Phys.* **2005**, *7* (18), 3297. https://doi.org/10.1039/b508541a.
(21) Kozuch, S.; Martin, J. M. L. Halogen Bonds: Benchmarks and Theoretical Analysis. *J. Chem. Theory Comput.* **2013**, *9* (4), 1918–1931. https://doi.org/10.1021/ct301064t.
(22) Marenich, A. V.; Cramer, C. J.; Truhlar, D. G. Universal Solvation Model Based on Solute Electron Density and on a Continuum Model of the Solvent Defined by the Bulk Dielectric Constant and Atomic Surface Tensions. *J. Phys. Chem. B* **2009**, *113* (18), 6378–6396. https://doi.org/10.1021/jp810292n.

(23) Neese, F.; Wennmohs, F.; Hansen, A.; Becker, U. Efficient, Approximate and Parallel Hartree–Fock and Hybrid DFT Calculations. A 'Chain-of-Spheres' Algorithm for the Hartree–Fock Exchange. *Chemical Physics* **2009**, *356* (1–3), 98–109. https://doi.org/10.1016/j.chemphys.2008.10.036.

(24) Young, T. Duartegroup/Otherm: Major Symmetry Improvements, 2020. https://doi.org/10.5281/ZENODO.3294010.

(25) Bickelhaupt, F. M.; Houk, K. N. Analyzing Reaction Rates with the Distortion/Interaction-Activation Strain Model. *Angew Chem Int Ed* **2017**, *56* (34), 10070–10086. https://doi.org/10.1002/anie.201701486.

(26) Grimme, S. Supramolecular Binding Thermodynamics by Dispersion-Corrected Density Functional Theory. *Chemistry – A European Journal* **2012**, *18* (32), 9955–9964. https://doi.org/10.1002/chem.201200497.

(27) Schrödinger, LLC. The PyMOL Molecular Graphics System, Version 1.8, 2015.

(28) Frisch, M. J.; Trucks, G. W.; Schlegel, H. B.; Scuseria, G. E.; Robb, M. A.; Cheeseman, J. R.; Scalmani, G.; Barone, V.; Mennucci, B.; Petersson, G. A.; Nakatsuji, H.; Caricato, M.; Li, X.; Hratchian, H. P.; Izmaylov, A. F.; Bloino, J.; Zheng, G.; Sonnenberg, J. L.; Hada, M.; Ehara, M.; Toyota, K.; Fukuda, R.; Hasegawa, J.; Ishida, M.; Nakajima, T.; Honda, Y.; Kitao, O.; Nakai, H.; Vreven, T.; Montgomery, J. A.; Peralta, J. E.; Ogliaro, F.; Bearpark, M.; Heyd, J. J.; Brothers, E.; Kudin, K. N.; Staroverov, V. N.; Kobayashi, R.; Normand, J.; Raghavachari, K.; Rendell, A.; Burant, J. C.; Iyengar, S. S.; Tomasi, J.; Cossi, M.; Rega, N.; Millam, J. M.; Klene, M.; Knox, J. E.; Cross, J. B.; Bakken, V.; Adamo, C.; Jaramillo, J.; Gomperts, R.; Stratmann, R. E.; Yazyev, O.; Austin, A. J.; Cammi, R.; Pomelli, C.; Ochterski, J. W.; Martin, R. L.; Morokuma, K.; Zakrzewski, V. G.; Voth, G. A.; Salvador, P.; Dannenberg, J. J.; Dapprich, S.; Daniels, A. D.; Farkas, Ö.; Foresman, J. B.; Ortiz, J. V.; Cioslowski, J.; Fox, D. J. Gaussian 09 Revision D.01, 2009.

(29) Singh, U. C.; Kollman, P. A. An Approach to Computing Electrostatic Charges for Molecules. *Journal of Computational Chemistry* **1984**, *5* (2), 129–145. https://doi.org/10.1002/jcc.540050204.

(30) Cieplak, P.; Cornell, W. D.; Bayly, C.; Kollman, P. A. Application of the Multimolecule and Multiconformational RESP Methodology to Biopolymers: Charge Derivation for DNA, RNA, and Proteins. *Journal of Computational Chemistry* **1995**, *16* (11), 1357–1377. https://doi.org/10.1002/jcc.540161106.

(31) Case, D. A.; Aktulga, H. M.; Belfon, K.; Cerutti, D. S.; Cisneros, G. A.; Cruzeiro, V. W. D.; Forouzesh, N.; Giese, T. J.; Götz, A. W.; Gohlke, H.; Izadi, S.; Kasavajhala, K.; Kaymak, M. C.; King, E.; Kurtzman, T.; Lee, T.-S.; Li, P.; Liu, J.; Luchko, T.; Luo, R.; Manathunga, M.; Machado, M. R.; Nguyen, H. M.; O'Hearn, K. A.; Onufriev, A. V.; Pan, F.; Pantano, S.; Qi, R.; Rahnamoun, A.; Risheh, A.; Schott-Verdugo, S.; Shajan, A.; Swails, J.; Wang, J.; Wei, H.; Wu, X.; Wu, Y.; Zhang, S.; Zhao, S.; Zhu, Q.; Cheatham, T. E. I.; Roe, D. R.; Roitberg, A.; Simmerling, C.; York, D. M.; Nagan, M. C.; Merz, K. M. Jr. AmberTools. *Journal of Chemical Information and Modeling* **2023**, *63* (20), 6183–6191. https://doi.org/10.1021/acs.jcim.3c01153.

(32) Kolář, M.; Hobza, P. On Extension of the Current Biomolecular Empirical Force Field for the Description of Halogen Bonds. *J. Chem. Theory Comput.* **2012**, *8* (4), 1325–1333. https://doi.org/10.1021/ct2008389.

(33) Ibrahim, M. A. A.; Telb, E. M. Z. σ-Hole and Lone-Pair Hole Interactions in Chalcogen-Containing Complexes: A Comparative Study. *ACS Omega* **2020**, *5* (34), 21631–21640. https://doi.org/10.1021/acsomega.0c02362.

(34) Caleman, C.; van Maaren, P. J.; Hong, M.; Hub, J. S.; Costa, L. T.; van der Spoel, D. Force Field Benchmark of Organic Liquids: Density, Enthalpy of Vaporization, Heat Capacities, Surface Tension, Isothermal Compressibility, Volumetric Expansion Coefficient, and Dielectric Constant. *J. Chem. Theory Comput.* **2012**, *8* (1), 61–74. https://doi.org/10.1021/ct200731v.

(35) Sengupta, A.; Li, Z.; Song, L. F.; Li, P.; Merz, K. M. Jr. Parameterization of Monovalent Ions for the OPC3, OPC, TIP3P-FB, and TIP4P-FB Water Models. *J. Chem. Inf. Model.* **2021**, *61* (2), 869–880. https://doi.org/10.1021/acs.jcim.0c01390.

(36) Kim, Y. C.; Chaloux, B. L.; Rolison, D. R.; Johannes, M. D.; Sassin, M. B. Molecular Dynamics Study of Hydroxide Ion Diffusion in Polymer Electrolytes. *Electrochemistry Communications* **2022**, *140*, 107334. https://doi.org/10.1016/j.elecom.2022.107334.
(37) Åqvist, J. Ion-Water Interaction Potentials Derived from Free Energy Perturbation Simulations. *J. Phys. Chem.* **1990**, *94* (21), 8021–8024. https://doi.org/10.1021/j100384a009.
(38) Grote, F.; Lyubartsev, A. P. Optimization of Slipids Force Field Parameters Describing Headgroups of Phospholipids. *J. Phys. Chem. B* **2020**, *124* (40), 8784–8793. https://doi.org/10.1021/acs.jpcb.0c06386.
(39) Jämbeck, J. P. M.; Lyubartsev, A. P. An Extension and Further Validation of an All-Atomistic Force Field for Biological Membranes. *J. Chem. Theory Comput.* **2012**, *8* (8), 2938–2948. https://doi.org/10.1021/ct300342n.
(40) Wang, L.-P.; Martinez, T. J.; Pande, V. S. Building Force Fields: An Automatic, Systematic, and Reproducible Approach. *J. Phys. Chem. Lett.* **2014**, *5* (11), 1885–1891. https://doi.org/10.1021/jz500737m.
(41) Darden, T.; York, D.; Pedersen, L. Particle Mesh Ewald: An N□log(N) Method for Ewald Sums in Large Systems. *The Journal of Chemical Physics* **1993**, *98* (12), 10089–10092. https://doi.org/10.1063/1.464397.
(42) Bussi, G.; Donadio, D.; Parrinello, M. Canonical Sampling through Velocity Rescaling. *The Journal of Chemical Physics* **2007**, *126* (1), 014101. https://doi.org/10.1063/1.2408420.
(43) Bernetti, M.; Bussi, G. Pressure Control Using Stochastic Cell Rescaling. *The Journal of Chemical Physics* **2020**, *153* (11), 114107. https://doi.org/10.1063/5.0020514.
(44) Hess, B.; Bekker, H.; Berendsen, H. J. C.; Fraaije, J. G. E. M. LINCS: A Linear Constraint Solver for Molecular Simulations. *Journal of Computational Chemistry* **1997**, *18* (12), 1463–1472. https://doi.org/10.1002/(SICI)1096-987X(199709)18:12<1463::AID-JCC4>3.0.CO;2-H.
(45) Lindahl; Abraham; Hess; Spoel, van der. GROMACS 2021.3 Source Code, 2021. https://doi.org/10.5281/zenodo.5053201.
(46) Bonomi, M.; Branduardi, D.; Bussi, G.; Camilloni, C.; Provasi, D.; Raiteri, P.; Donadio, D.; Marinelli, F.; Pietrucci, F.; Broglia, R. A.; Parrinello, M. PLUMED: A Portable Plugin for Free-Energy Calculations with Molecular Dynamics. *Computer Physics Communications* **2009**, *180* (10), 1961–1972. https://doi.org/10.1016/j.cpc.2009.05.011.
(47) Marques, I.; Costa, P. M. R.; Q. Miranda, M.; Busschaert, N.; Howe, E. N. W.; Clarke, H. J.; Haynes, C. J. E.; Kirby, I. L.; Rodilla, A. M.; Pérez-Tomás, R.; Gale, P. A.; Félix, V. Full Elucidation of the Transmembrane Anion Transport Mechanism of Squaramides Using *in Silico* Investigations. *Phys. Chem. Chem. Phys.* **2018**, *20* (32), 20796–20811. https://doi.org/10.1039/C8CP02576B.
(48) Spooner, M. J.; Li, H.; Marques, I.; Costa, P. M. R.; Wu, X.; Howe, E. N. W.; Busschaert, N.; Moore, S. J.; Light, M. E.; Sheppard, D. N.; Félix, V.; Gale, P. A. Fluorinated Synthetic Anion Carriers: Experimental and Computational Insights into Transmembrane Chloride Transport. *Chem. Sci.* **2019**, *10* (7), 1976–1985. https://doi.org/10.1039/C8SC05155K.
(49) Edwards, S. J.; Marques, I.; Dias, C. M.; Tromans, R. A.; Lees, N. R.; Félix, V.; Valkenier, H.; Davis, A. P. Tilting and Tumbling in Transmembrane Anion Carriers: Activity Tuning through n-Alkyl Substitution. *Chem. Eur. J.* **2016**, *22* (6), 2004–2011. https://doi.org/10.1002/chem.201504057.
